# Indole–Pyrazole Hybrids: Synthesis, Structure, and Assessment of Their Hemolytic and Cytoprotective Properties

**DOI:** 10.3390/ijms26189018

**Published:** 2025-09-16

**Authors:** Karolina Babijczuk, Klaudia Wawrzyniak, Beata Warżajtis, Urszula Rychlewska, Damian Nowak, Yunna da Victoria Banda, Lucyna Mrówczyńska, Beata Jasiewicz

**Affiliations:** 1 Department of Bioactive Products, Faculty of Chemistry, Adam Mickiewicz University, Uniwersytetu Poznańskiego 8, 61-614 Poznan, Poland; karolina.babijczuk@amu.edu.pl (K.B.); klawaw6@st.amu.edu.pl (K.W.); 2 Department of Crystallography, Faculty of Chemistry, Adam Mickiewicz University, Uniwersytetu Poznańskiego 8, 61-614 Poznan, Poland; beataw@amu.edu.pl (B.W.); urszular@amu.edu.pl (U.R.); 3 Department of Quantum Chemistry, Faculty of Chemistry, Adam Mickiewicz University in Poznań, Uniwersytetu Poznańskiego 8, 61-614 Poznan, Poland; damian.nowak@amu.edu.pl; 4 Department of Biological Science, Faculty of Science, Eduardo Mondlane University, Praça 25 de Junho, P.O. Box 257, Maputo 257, Mozambique; yunna.band@gmail.com; 5 Department of Cell Biology, Faculty of Biology, Adam Mickiewicz University, Uniwersytetu Poznańskiego 6, 61-614 Poznan, Poland

**Keywords:** gramine, indole derivatives, human erythrocytes, cytoprotective properties, tautomer-selective reaction

## Abstract

In recent years, we have observed a growing interest in molecular hybridization, which involves combining chemically and pharmacologically diverse fragments into a single molecule. In this study, we designed and synthesized a series of indole–pyrazole hybrids, variously substituted at the pyrazole ring. The compounds were characterized by spectroscopic methods, and the structures of most of them were confirmed by X-ray analysis. Reactions of 3-(dimethylaminomethyl)indole with bromo-methyl-pyrazole derivatives proceeded in a tautomer-selective mode: the 4-bromo-3(5)-methyl-((1*H*-pyrazol-1-yl)methyl)-1*H*-indole tautomers, obtained from the 4-bromo-3-methyl-1*H*-pyrazole, could be isolated by column chromatography. In contrast, the 3-bromo-5-methyl-1*H*-pyrazole yielded the ((5-bromo-3-methyl-1H-pyrazol-1-yl)methyl)-1H-indole as the dominant reaction product. The 3-bromo-5-methyl tautomer could not be isolated nor could its presence be identified in solution. However, traces of it were recognized in the crystal of 5-bromo-3-methyl tautomer as a binary solid solution. In silico studies provided the physicochemical parameters of all compounds, enabling the estimation of some derivatives affinity to certain enzymes. In vitro evaluation of the hemolytic and cytoprotective properties of all derivatives showed that most of the compounds exhibited no hemolytic activity, while all demonstrated significant cytoprotective effects on human erythrocytes under oxidative stress.

## 1. Introduction

One strategy for obtaining new bioactive compounds is to create hybrid molecules from biologically active substrates, resulting in conjugates with enhanced bioactivity. A common approach is to combine five-membered nitrogen-containing heterocycles, known as azoles, with 3-substituted indole derivatives. Pyrazoles and imidazoles form a broad class of azole compounds with diverse structures and various biological effects [1,2,3,4]. The presence of N-N bonds makes pyrazole less stable than imidazole [5] and less prevalent in nature [6]. However, pyrazole derivatives exhibit various biological properties, including antioxidant [7], antidiabetic [7], and anticancer [8] effects. They are also widely used in many commercially available drugs [2,9].

Indoles are known for their wide range of biological activities, such as antioxidant, anticancer, antibacterial, and antifungal effects [10,11]. Additionally, indoles are used to develop new anti-inflammatory drugs and treatments for conditions such as Alzheimer’s disease, Parkinson’s disease, diabetes, HIV, and SARS-CoV-2 [12,13,14,15,16,17].

Hybrids containing indole and pyrazole groups have been widely studied [18,19]. These hybrids are known for their anticancer [20,21] and antioxidant [22] activities. They may also exhibit anticonvulsant properties [23]. Another common medicinal chemistry practice aimed at increasing bioactivity is the modification of substituents. For example, replacing a hydrogen atom with a functional group or substituent (such as an alkyl or halogen) can significantly alter a molecule’s biological activity. Therefore, we considered it worthwhile to validate the synergistic integration of C3-substituted indole and variously substituted azole derivatives and to compare the biological activity of the resulting hybrid compounds. Our previous study on indole-imidazole hybrids demonstrated that the type of substituent in the imidazole ring significantly affects the hemolytic and cytoprotective properties of its derivatives [24]. Specifically, alkyl substituents enhance cytoprotective effects by delocalizing the resulting radicals, while halogens increase hemolytic activity. Given this finding and our ongoing research on bioactive indole derivatives [24,25,26], we have extended our investigations to indole–pyrazole hybrids, focusing on their hemolytic and cytoprotective properties.

**Figure 1:**
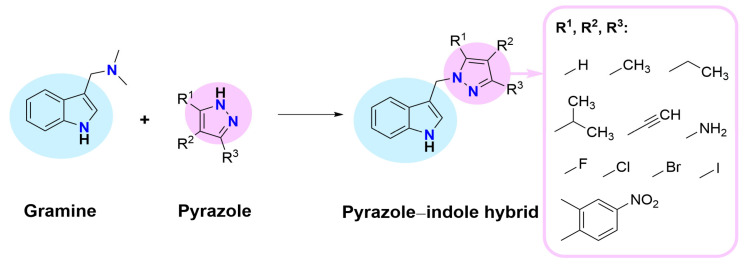
Design of pyrazole–indole hybrids.

Figure 1 shows the structural heterocyclic scaffolds used to synthesize the target compounds.

Oxidative stress, defined as an imbalance between pro- and antioxidants at the cellular level that favors the former, is a key factor in the development of many chronic diseases and aging processes [27]. Therefore, identifying new compounds that prevent oxidative stress is a crucial part of drug discovery. Red blood cells (RBCs) are particularly susceptible to oxidative damage due to their role in oxygen transport and their membrane structure, which contains high levels of polyunsaturated fatty acids in the lipid bilayer [28]. Exposure of RBCs to reactive oxygen species (ROS) can damage the molecular structure of their membrane, increasing permeability to ions and leading to oxidative stress-induced hemolysis. Hemolysis may also result from alteration the molecular structure of the cell membrane caused by the incorporation of biologically active molecules into its lipid bilayer [29]. These molecules can either disrupt the membrane’s integrity and cause hemolysis or stabilize it, thereby protecting the cell against the membrane damage. The effects of bioactive compounds depend on (1) their chemical structure and (2) the concentration to which the cells are exposed [30]. Compounds that do not cause hemolysis are considered hemocompatible [29].

Our research aimed to develop novel indole–pyrazole hybrids and assess their ability to protect red blood cells from oxidative stress and membrane damage, thereby preventing hemolysis. This protection is crucial for the development of therapeutic agents and the assessment of the biocompatibility of new compounds. Consequently, sixteen compounds were synthesized using gramine as the starting compound. The structures of the new hybrids were confirmed, and their hemolytic and cytoprotective properties were evaluated.

Comparison of the analogous indole–pyrazole and indole-imidazole conjugates allowed us to determine how the position of nitrogen atoms within the azole ring influences the hemolytic and cytoprotective properties of the compounds. A molecular mechanism underlying the hemolytic and cytoprotective activity was proposed. A molecular docking studies were performed to estimate the affinity of the selected derivatives for three enzymes (myeloperoxidase, MPO; xanthine dehydrogenase; and cyclooxygenase-2, COX-2) involved in oxidative stress [31,32,33]. Additionally, in silico analysis were conducted to evaluate the physicochemical properties of all compounds.

## 2. Results and Discussion

### 2.1. Synthesis and Spectroscopic Characterization of Indole Hybrids

**Scheme 1:**
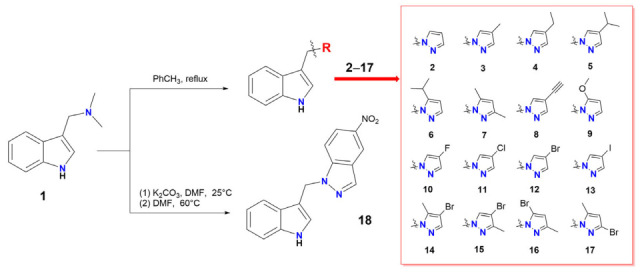
Synthesis of indole–pyrazole hybrids **2**–**18**.

Gramine was used as the starting compound in the synthesis. This indole alkaloid is commonly used to produce indole-based compounds [34]. The dimethylamine group at the C3 position of the indole ring in gramine acts as an effective leaving group. The resulting intermediate immediately reacts with a nucleophile [24]. We synthesized indole–pyrazole hybrids using the same methodology applied to the synthesis of similar imidazole derivatives [24]. However, these reactions required an excess of the pyrazole substrate and longer heating times. This resulted in lower efficiency compared to the synthesis of indole-imidazole bioconjugates. The lowest yield was observed for derivative **8** (12%), which is due to the presence of an ethynyl substituent. All products were obtained with a twofold excess of pyrazole relative to gramine (see Scheme 1). The reactions involved heating the substrates in toluene for 9 to 20 h. Based on our previous findings with imidazole and benzimidazole derivatives, we expanded our research to include an indazole (benzopyrazole) derivative (compound **18**). The synthesis of compound **18** required basic conditions, a different solvent (DMF), and a lower temperature (60 °C for 15 h). The yield was 14% due to the presence of an NO_2_ group on the indazole ring. Compounds **2** and **7** were previously prepared from a gramine substrate by Cravotto et al. [34]. However, the synthetic procedure was not sufficiently described, and the derivatives were not tested for biological activity. The reactions with 5-isopropylpyrazole and 5-methoxypyrazole produce only one product, which can be attributed to substituent preferences, intramolecular interactions, or the influence of reaction conditions [35,36]. In contrast, the reaction with 4-bromo-3-methylpyrazole yields two distinct products (**14** and **15**), which were isolated using a chromatographic column. The ratio of the product featuring a methyl group at the C5 position to the product with a methyl substituent at the C3 position was 0.36:0.64, consistent with the various syntheses involving 4-bromo-3-methylpyrazole described in the literature [37,38]. Quite unexpectedly, the reaction with 3-bromo-5-methylpyrazole appeared to yield only the 5-bromo-3-methyl tautomer **16**, with no other products identified in the solution. However, X-ray analysis of **16** revealed the crystal contains a mixture of tautomers **16** and **17**. The compounds crystallized as a solid solution, with the two components disordered over the same crystallographic site in a ratio of 0.92:0.08. In substitutional solid solutions, the two components are randomly inserted into the crystal lattice. Therefore, their ratio is not necessarily an integer.

The ^1^H NMR spectra of all newly synthesized compounds (**2**–**16**, **18**) show diagnostic signals for the aromatic rings of the indole system in the range of 6.94–7.61 ppm. In addition, signals from pyrazole (5.61–8.02 ppm; for **2**–**16**) and indazole (8.03–8.80 ppm; for **18**) substituents are observed in the aromatic region. The characteristic hydrogen singlet at about 11 ppm is assigned to the N-H proton of the indole moiety. Signals from C10 protons (methylene bridge) resonate as singlets in the 5.18–5.87 ppm range. The protons of the methyl group (**2**–**7**, **9**, **14**–**16**) are observed at 1.07–3.86 ppm, while those of the CH_2_ and CH groups are observed at 2.35 (**4**), 2.72 (**5**), 3.16 (**6**), and 3.93 (**8**) ppm, respectively.

In the ^13^C NMR spectra, signals from the carbon atoms of the indole ring are observed in the range of 109.2–138.1 ppm, the pyrazole ring (**2**–**16**) in the range of 84.4–154.5 ppm, and the indazole ring (**18**) in the range of 109.8–141.5 ppm. Signals from C10 are observed in the range of 41.9 to 48.1 ppm. The methyl group in **3**–**7** and **14**–**16** gives signals in the 8.7–23.8 ppm range, whereas the signal from the -CH_3_ in the methoxy group (**9**) is observed at 58.8 ppm. At 16.9 ppm, a signal from -CH_2_ in the ethyl group in **4** was observed. In compounds **5** and **6**, containing the isopropyl group, signals from -CH are found at 23.9 and 24.5 ppm. Two signals from the ethynyl group (**8**) are observed at 75.8 and 80.8 ppm. The carbon atom connected to the nitro group in **17** gives a signal at 141.5 ppm. In halogen derivatives, the carbon atom attached to the Cl atom is at 107.8 ppm (**11**), and the Br atom is at 91.5–118.7 ppm (**12**, **14**–**16**). In the case of compounds with fluorine (**10**) and iodine (**13**), signals from carbons attached to halogens are split at 147.7 and 150.1ppm for **10**, and 56.5 and 56.5 ppm for **13**.

In FT-IR spectra, vibrations of the unsubstituted N-H group in the indole moiety are observed at about 3400 cm^−1^. Absorption bands at 2800–3050 cm^−1^ and near 1600 cm^−1^ correspond to the C-H bonds of aromatic rings for all compounds. Bands with maximum absorption between 2800 and 3000 cm^−1^ correlate with C-H bonds of aliphatic chains (**3**–**7**, **14**–**16**). In contrast, bands corresponding to C-H bonds of ethynyl and methoxy groups are observed at 3283 and 2828 cm^−1^, respectively. The characteristic band at 2117 cm^−1^ in the spectrum of **8** results from vibrations of the C≡C group. Bands originating from C=C and C=N are observed in the same region at 1500–1660 cm^−1^. The NO_2_ group exhibits three bands: asymmetric stretching vibrations at 1516 cm^−1^, symmetric stretching vibrations at 1340 cm^−1^, and scissoring vibrations at 857 cm^−1^. The characteristic bands of the indole ring are present at 580–645 cm^−1^.

In the EI–MS spectra of compounds **2**–**16** and **18**, molecular ions were observed with relative abundances ranging from 19 to 100%. Furthermore, the indole ion (*m*/*z* = 129 or 130) was identified in all spectra, with relative abundances ranging from 19 to 100%.

The NMR (1H and 13C), EI-MS, and FT-IR spectra of all indole–pyrazole hybrids are included in the Supplementary Materials (Figures S1–S16).

### 2.2. X-Ray Analysis

**Figure 2:**
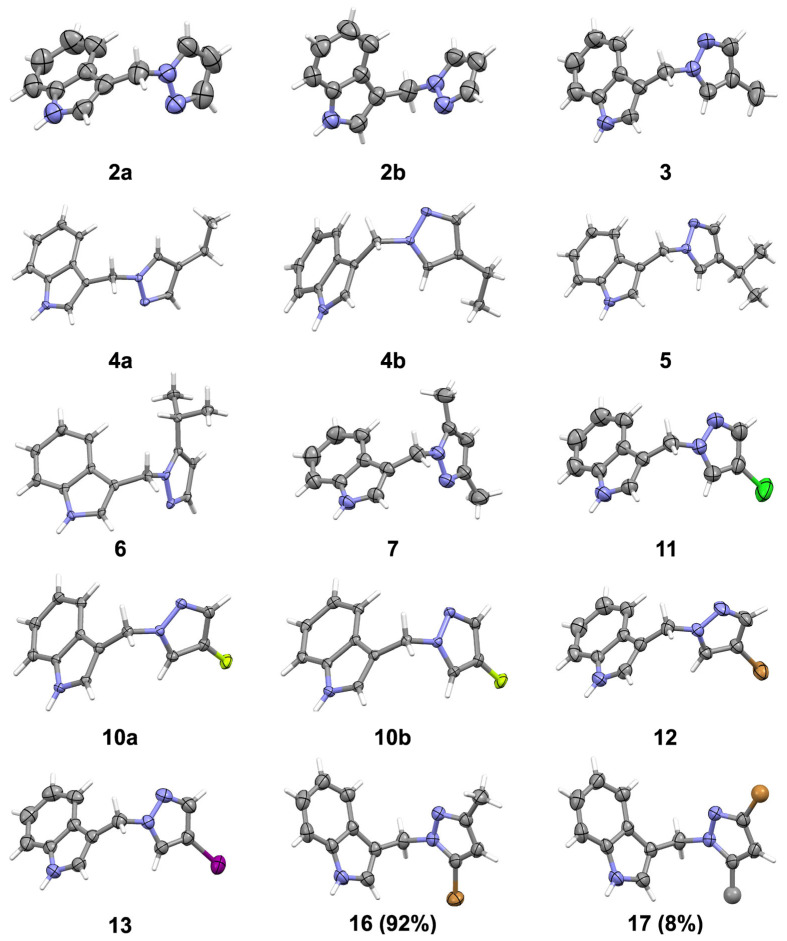
Perspective views of fifteen molecular conformers present in crystals of **2** (two independent molecules), **3**, **4** (two independent molecules), **5**–**7**, **10** (two independent molecules), **11**–**13,** and **16** with thermal ellipsoids drawn at the 50% probability level. Structures **4**–**6** and **10** were determined at 100 K, and the remaining structures were determined at room temperature. Crystal of **16** exists as a binary solid solution of constitutional tautomers **16** and **17**. Methyl H-atoms in **17** were not localized.

Fifteen molecular structures present in eleven crystals studied by X-ray diffraction are shown in Figure 2. Compounds **16** and **17** crystallize together as a substitutional solid solution of 3-((5-bromo-3-methyl-1*H*-pyrazol-1-yl)methyl)-1*H*-indole and 3-((3-bromo-5-methyl-1*H*-pyrazol-1-yl)methyl)-1*H*-indole in a 0.92:0.08 ratio, respectively.

The molecular conformation is described by a pair of torsion angles (φ_1_ and φ_2_) listed in Table 1, measured along the C-C-C-N and C-C-N-C methylene bonds connecting the indole and pyrazole rings. To enable comparison, Table 1 also provides chemical diagrams and a capped stick representation of the molecules, all shown in the same orientation, i.e., along the indole plane with the N-H(indole) group directed toward the reader. The absolute values of φ_1_ vary from 53.5(4) to 97.7(3)° while those of φ_2_ cover a much wider range from 24.5(4) to 124.5(3)°. In nine out of fifteen molecules, the φ_1_ and φ_2_ torsion angles are paired in sign, and differences in their absolute values vary from 0.0 to 46.3°. On average, in this type of conformer, the two nitrogen atoms are separated by 5.601 Å (the contribution of the minor component in a crystal of **16** was not counted). A similar molecular architecture has been observed in alkyl-substituted indole-imidazole hybrids [24]. We ascribe these conformers to type I. The conformation of the remaining four molecules (**2a** and **2b**, **4a**, **6**, and **7**) is significantly different: the φ_1_ and φ_2_ torsion angles differ in sign and display high φ_2_ values. In these molecules, the differences between φ_1_ and φ_2_ absolute values reach up to 71°, and the average N∙∙∙N separation amounts to 4.644 Å. We ascribe these conformers to type II. Both types of conformers are concomitantly present in crystals of **4.**

When viewed along the indole plane with the N-H group pointing toward the observer, the type I molecules have the basic pyrazole nitrogen atom oriented away from the reader. In contrast, the type II conformers have this nitrogen pointing toward the reader (Figure 2 and Table 1). The two types of molecular conformation demonstrate the molecules’ adaptation to the crystal packing requirements, which are dominated by hydrogen bonds.

The species investigated are both hydrogen-bond donor and acceptor molecules, owing to their N-H (indole) and N (pyrazole) centers. The mode of association of these hybrid molecules resembles that characteristic of 1-*H* pyrazole and its C-substituted derivatives.

**Figure 3:**
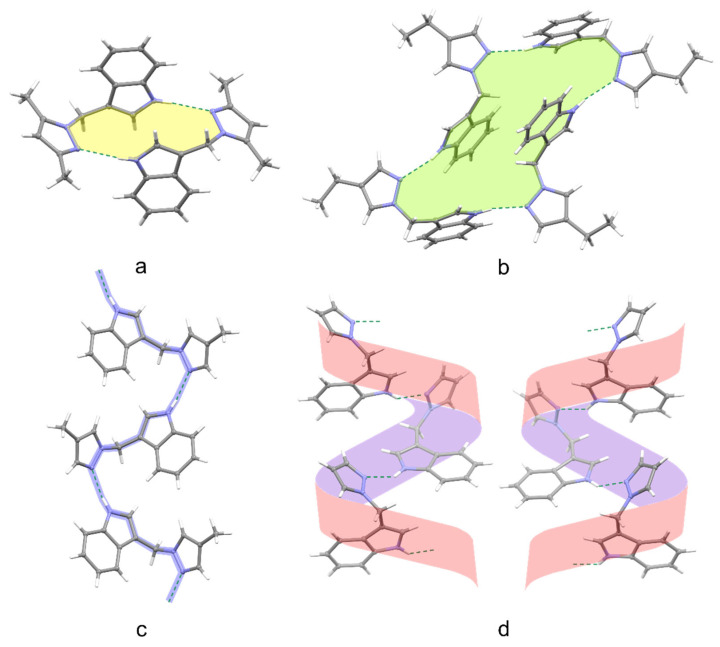
Representation of supramolecular assemblies formed from self-association of the investigated indole–pyrazole hybrids with the N–H(indole)∙∙∙N(pyrazole) hydrogen bonds, represented with dashed lines: (**a**) dimers in crystals of **7**, (**b**) tetramers in crystals of **4**, (**c**) chains in crystals of **3**, (**d**) two opposing helices M and P in crystals of **2**.

Both types of structures contain one H-bond donor and one H-bond acceptor center. However, the 1-*H* pyrazole derivatives have both an H-bond donor and an acceptor in the closest proximity. At the same time, in the investigated hybrid molecules, the two centers are roughly 5 Å apart, as they belong to two different aromatic moieties. The indole N—H proton donates an N—H∙∙∙N hydrogen bond to a neighboring pyrazole N atom (Figure 3). Pyrazoles are known to crystallize in a variety of hydrogen-bonded motifs—dimers, trimers, tetramers, and chains (sometimes called catemers) being a function of the size of the substituents [39]. Similarly, the molecules studied also form dimers (**7**) or tetramers (**4**) (Figure 3). Still, they mainly consist of chains formed by molecules related by a glide plane (**3**, **5**, **6**, **11**, **12**, **13**) or, exceptionally, built of two separate molecules with opposite helicities (**10**). Helices around a two-fold screw axis are observed in crystals **2**, **6**, and **16**, which display solely a rotational symmetry, described by one of the Sohncke space groups. Crystals of **2** are particularly interesting in that they contain two symmetry-independent molecules that are conformational quasi-enantiomers (Figure 2, Table 1). The molecules are linked by intermolecular N–H∙∙∙N hydrogen bonds into helical chains (Figure 3d), each composed of only one of the two independent molecules. Hence, although the crystal possesses the Sohncke symmetry, the unit cell contains two enantiomeric helices, the M and the P.

There is a clear correspondence between a type of molecular conformer and a type of hydrogen bond association in a crystal. The most common motif, the chains, is formed between molecules, which adopt the type I conformation (characterized by a set of ϕ1 and ϕ2 torsion angles of the same sign and roughly similar magnitude, approximately 62° on average). On the other hand, molecules taking the type II conformation (where ϕ_1_ and ϕ_2_ differ in sign and the magnitude of ϕ_2_ is about 120°) either form helices (**2**, **6**) or associate into discreet dimers (**7**) or tetramers (**4**). Exceptional to this generalization is the solid-solution of tautomers **16** and **17**, in which the helical assembly is combined with the conformation typified by ϕ_1_ and ϕ_2_ equal both in sign and magnitude (nearly 98°). Although association via NH∙∙∙N hydrogen bonds dominates the packing, in crystals of **12,** we observe the presence of additional pairwise π∙∙∙π stacking interactions, which are unique to the investigated series. The distance between pyrazole∙∙∙pyrole and pyrole∙∙∙pyrazole mean planes equals 3.331 and 3.384 Å, respectively; the dihedral angle between pyrazole∙∙∙pyrole mean planes amounts to 8.95°, and the centroid∙∙∙centroid distance is 3.658 Å. Also worth noting is the isostructurality of crystals **3** and **11**, which contain, respectively, methyl and chlorine substituents at the C4 position of the pyrazole. The isomorphism resulting from the Me/Cl substitution is not uncommon and is interpreted as based on the size of the substituent [40].

### 2.3. Hemolytic Properties and Cytoprotective Activity Against Free Radical-Induced Hemolysis

**Figure 4:**
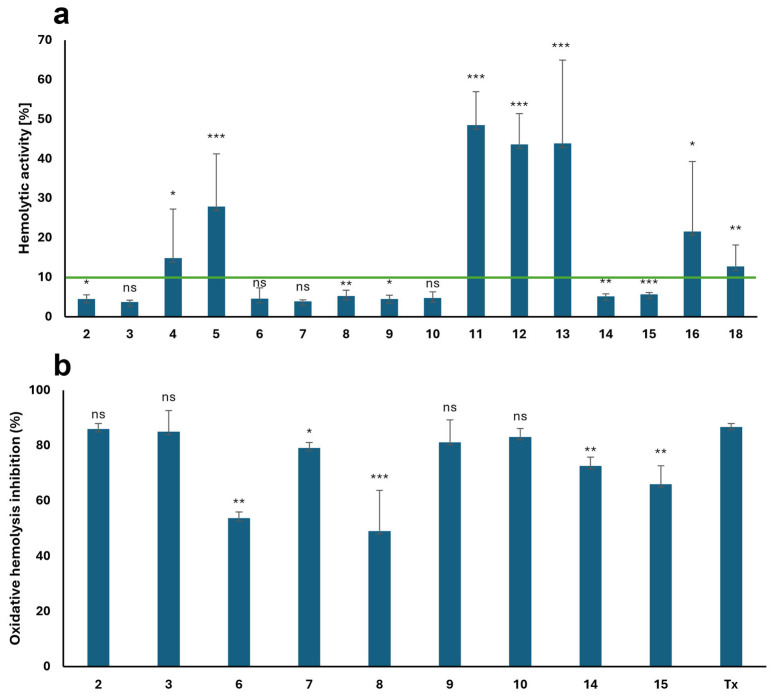
(**a**). Hemolytic activity of compounds **2**–**18** at a concentration of 0.1 mg/mL. Results (*n* = 9) are presented as the mean value ± standard deviation (* *p* < 0.05, ** *p* < 0.01, and *** *p* < 0.001) compared to hemolysis in PBS. Non statistically significant differences (*p* > 0.05) are indicated as ns. The green line represents the hemolysis threshold of 10%. (**b**) Cytoprotective activity of compounds **2**–**3**, **6**–**10**, **14**, **15**, and the reference antioxidant Trolox (Tx) against oxidative stress-induced hemolysis. Results (*n* = 9) are presented as the mean values ± standard deviation (* *p* < 0.05, ** *p* < 0.01, and *** *p* < 0.001) compared to Tx. Non statistically significant differences (*p* > 0.05) are indicated as ns.

The hemocompatibility of sixteen indole–pyrazole hybrids (Figure 4a) with different substituents was assessed in vitro using human RBCs as a cell model. A concentration of 0.1 mg/mL was previously used to evaluate the properties of indole-imidazole hybrids [24]. Figure 4a shows that most indole–pyrazole hybrids are hemocompatible, with low hemolytic activity ranging from 3.67 ± 0.47% to 5.64 ± 0.49%. These results show a correlation between the position of the substituents on the pyrazole ring and hemolytic activity. This is exemplified by comparing the hemolytic activity of compounds **5** and **6**. Derivative **5**, which features an isopropyl group at the C4 position, exhibits hemolytic activity that is more than five times higher (27.90 ± 13.33%) than that of derivative **6**, which has an isopropyl group at the C5 position and exhibits hemolytic activity of 4.54 ± 2.72%. Another example is derivatives **14**–**16**, which have a pyrazole ring substituted with a methyl group and a bromine atom. Compound **14**, featuring a methyl group at the C5 position and a bromine atom at the C4 position, exhibits a hemolytic activity of 5.13 ± 0.64%. Its tautomer, compound **15**, exhibits a slightly higher activity at 5.64 ± 0.49%. In contrast, compound **16**, which has a methyl group at the C3 position and a bromine atom at the C5 position, exhibits significantly higher hemolytic activity at 21.57 ± 17.74%. Most hemolytic compounds are hybrids with electron-withdrawing substituents at the C4 position, such as chlorine (compound **11**; 48.46 ± 8.49%), bromine (compound **12**; 43.64 ± 7.74%), and iodine (compound **13**; 43.85 ± 21.08%). Conversely, the derivative with a fluorine atom (compound **10**) shows significantly lower hemolytic activity at 4.71 ± 1.56%.

The impact of substituents on the hemolytic activity of the indole–pyrazole hybrids differs from that observed for the indole-imidazole derivatives. Previous studies [24] have demonstrated that indole-imidazole derivatives with alkyl substituents (methyl, ethyl, or isopropyl) in the imidazole ring or an unsubstituted imidazole ring exhibit hemolytic activities below 5%, ranging from 1.26 ± 0.27% to 3.61 ± 1.19%. In comparison, the lowest observed hemolytic activity for the indole–pyrazole derivatives with an alkyl substituent is exhibited by compound **3**, which contains a methyl group at the C4 position. Compounds with ethyl or isopropyl substituents at the C4 position (compounds **4** and **5**) exhibited hemolytic activities of 14.82 ± 12.43% and 27.90 ± 13.33%, respectively.

Compounds **2**, **3**, **6**-**10**, **14**, and **15** exhibited less than 10% hemolytic activity and were further evaluated in vitro for their cytoprotective effects under oxidative stress conditions. Figure 4b shows that all tested compounds exhibited significant cytoprotective properties (over 50%) under oxidative stress conditions. The efficacy of compounds **2** (85.99 ± 2.00%) and **3** (84.94 ± 7.73%) in protecting erythrocytes against oxidative hemolysis was comparable to that of the standard antioxidant Trolox (86.67 ± 1.28%). Furthermore, compounds **7**, **9**, and **10** exhibited notable cytoprotective efficacy, with activities of 79.12 ± 2.02%, 81.21 ± 8.09%, and 83.12 ± 3.08%, respectively. The remaining hybrids showed cytoprotective activity ranging from 49.06 ± 14.69% to 72.56 ± 3.24%. The increase in cytoprotective activity among these compounds followed the order of substituents: ethynyl (**8**) < 5-isopropyl (**6**) < 4-bromo-3-methyl (**15**) < 3-bromo-4-methyl (**14**).

Analysis of Figure 4b indicates that either unsubstituted pyrazole derivatives or those with small substituents at C-4 exhibit the highest cytoprotective effects, comparable to Trolox. This contrasts with the significant positive effect of alkyl group substitution observed in the analogous indole-imidazole hybrids previously studied by our group [24].

Indole–pyrazole hybrids can interact with peroxyl radicals generated from AAPH via both single-electron transfer (SET) and hydrogen atom transfer (HAT) mechanisms. It has been established that an unsubstituted NH group in the indole ring is crucial for the HAT mechanism of indoles [41]. In this mechanism, a hydrogen atom is transferred directly from the antioxidant to the active radical, resulting in the formation of a nitrogen-centered indolyl radical. In contrast, the SET mechanism involves aromatic rings acting as electron donors, leading to the formation of a stabilized radical cation following electron loss [42,43]. Introducing an electron-withdrawing substituent at C3 of the indole ring can enhance the tendency to form an indolyl radical anion by accepting an electron.

Our findings demonstrate that various functional groups attached to the C3 position of indole via a methylene linker can significantly modify the cytoprotective properties of the parent compound. Specifically, replacing an imidazole with a pyrazole has an effect. Moreover, the introduction of an alkyl substituent(s) to either of the two rings has an opposite impact on the cytoprotective activity: enhancing the activity of imidazole derivatives and weakening that of pyrazole derivatives.

### 2.4. Molecular Docking

Compounds **2**, **3**, and **10** were selected for molecular docking based on their high cytoprotective activity, which is comparable to that of the standard antioxidant Trolox. The selection of protein domains (1DNU—Myeloperoxidase (MPO), 1N5X—Xanthine dehydrogenase, 4COX—COX-2) was guided by their specific biological activities [31,32,33]. Melatonin, febuxostat, and indomethacin were used as endogenous ligands.

**Similarities and Differences between Novel and Endogenous Ligands**

To visualize the differences between native ligands and new ligands, the Lipinski and Veber parameters for melatonin, febuxostat, and indomethacin were calculated and are presented in Table 2. The reference ligands have higher molecular weight and polar surface area than the investigated compounds. They also have lipophilicity similar to that of indole–pyrazole hybrids. This suggests that the newly synthesized indole derivatives may have comparable solubility and absorption profiles to those of the reference ligands [44]. The reference ligands have more rotational bonds, indicating that the new derivatives are more flexible.

The molecular docking data revealed that the newly acquired derivatives exhibit affinity for the investigated protein domains (see Table 3). Their affinity to the 1DNU protein domain is comparable to that of the reference ligand, melatonin. However, the ProteinsPlus algorithms—specifically PoseView [45,46] and PoseEdit [45,47]—failed to produce 2D interaction maps. The error messages “No interactions found by the PoseView interaction model” and “The ligand was not found in a GeoMine binding site, or the ligand does not form any interactions” indicate that some of the ligands lack 2D depictions of their interactions with the protein domain. Additionally, the native NAG ligand forms a covalent bond with the ASN 189 C protein residue. This bonding causes discrepancies in the redocking procedure when compared to the original position of the native NAG ligand. Regarding the 1N5X protein domain, the docked compounds demonstrate higher affinity than the reference ligand, febuxostat. Conversely, for the 4COX protein domain, all the compounds exhibit slightly lower affinities than the native ligand, indomethacin. These findings suggest that the binding strengths of these protein domains differ from those of the reference ligands.

To normalize for ligand size and facilitate direct comparison across molecules of different structures, ligand efficiencies were computed as the ratio of binding energy to the number of heavy (non-hydrogen) atoms. The numbers of atoms are as follows for the following structures: melatonin-17; structure 2–15; structure 3–16; structure 10–16; febuxostat-22; indomethacin-25. The ligand efficiencies are given in Table 3. Notably, ligand efficiencies for the novel derivatives surpass those of the reference ligands in all protein domains. This finding highlights the favorable ratio of binding strength to ligand size for the new molecules, an important criterion in rational drug design.

**Figure 5:**
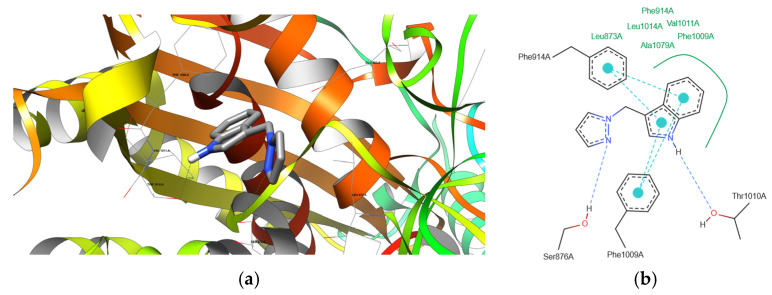

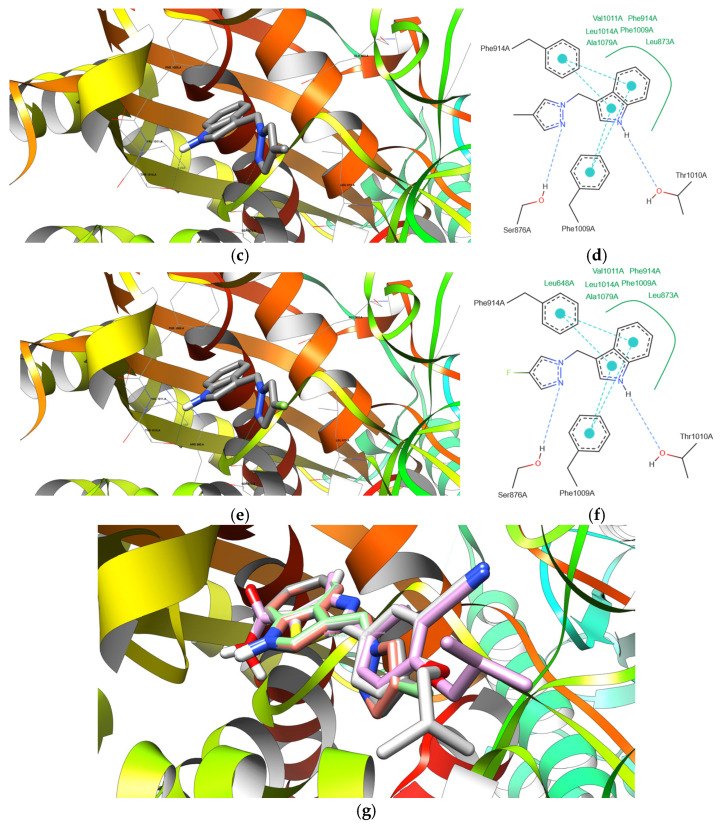
The depiction of possible hydrogen bond formation between the 1N5X protein domain and compounds **2** (**a**), **3** (**c**), and **10** (**e**). The 2D depiction of interactions between the 1N5X protein domain and compounds **2** (**b**), **3** (**d**), and **10** (**f**). Blue dashed lines—hydrogen bonds, cyan lines with big dots—pi-pi interactions, green solid lines—hydrophobic contacts (right). Native ligands (TEI), reference ligands (febuxostat), and new ligands at once in the binding site of a 1N5X protein domain (**g**).

Visual representations (Figure S17, supplementary part) illustrate the interactions between docked derivatives and the 1DNU protein domain (PDB ID). Figure 5a–g depict interactions with the 1N5X protein domain (PDB ID). Notably, the recreation of the native ligand’s initial pose (TEI) in the latter case achieves very good accuracy, with a Root Mean Square Deviation (RMSD) of 0.949 Å and a binding energy of −7.5 kcal/mol [32]. Figure S18 (supplementary part) shows interactions with the 4COX protein domain (PDB ID), where the recreation of the native ligand’s initial pose also exhibits similar accuracy, with an RMSD of 0.956 Å and a binding energy of −9.8 kcal/mol [48]. These visualizations offer insights into the intricate molecular interactions that underlie the binding of derivatives to protein domains, reinforcing their potential as candidates for further exploration and development.

The studies reveal that the analyzed ligands exhibit affinity profiles comparable to or higher than the reference ligands (melatonin for 1DNU and febuxostat for 1N5X). In the case of the 4COX protein domain, the affinities are lower than those of the reference ligand indomethacin. This suggests that these ligands may possess better antioxidant properties than the reference ones.

Table 4 summarizes all the predicted hydrogen bonds formed between the protein domains and the ligands. This table includes only the most probable hydrogen bonds, specifically highlighting those with the highest likelihood when multiple possibilities exist between certain atoms of a ligand and a protein domain. Hydrogen bonds with lengths greater than 3.4 Å are excluded from this table, as their likelihood of formation is very low [49].

### 2.5. Physicochemical Properties of Indole Derivatives—In Silico Study

The Lipinski and Veber rules provide criteria for assessing the drugability of compounds. According to Lipinski’s rule, a compound should have a molecular weight (MW) of less than 500 g/mol, an octanol/water partition coefficient (logP) of less than 5, no more than five hydrogen bond donors (HBD), and 10 hydrogen bond acceptors (HBA) [50]. Veber’s rule considers rotatable bonds (RTB) to be less than 10, and the total polar surface area (TPSA) should not exceed 140 Å^2^ [51]. Other important drug parameters include gastrointestinal (GI) absorption, the blood–brain barrier (BBB) permeability, and solubility in water. Upon oral administration, the drug is primarily absorbed in the gastrointestinal tract and enters the bloodstream, where it may cross the blood–brain barrier by passive diffusion [52]. The physicochemical properties of the derivatives were evaluated using the SwissADME website [53], and the results are presented in Table 5. Table 5 demonstrates that the newly synthesized compounds, with log P values ranging from 1.97 to 3.02, exhibit good lipophilicity, enabling them to traverse the lipid bilayer of the cell membrane. All derivatives met Lipinski’s and Veber’s rules and showed high GI absorption. Except for compound **18**, all compounds were able to penetrate the BBB, suggesting their potential activity in the central nervous system. Furthermore, all new indole–pyrazole hybrids exhibited moderate to good solubility in water.

To explain the observed hemolytic activity of new derivatives (Figure 4a), the molecular descriptors presented in Table 2 and Table 5 were considered. Compounds **4**, **5**, **11**, **12**, **13**, **16**, and **18** were hemolytic, with the strongest activity for **11** and **13**, whereas all other derivatives were non-hemolytic. Compounds with hemolytic activity were generally characterized by moderate molecular weights, logP values between 2.5 and 3.0, low TPSA (~33 Å^2^), and limited solubility (LogS below −4.0 in several cases), favoring their accumulation in the lipid bilayer of RBCs membranes. Compound **18**, despite its higher polarity (TPSA 79.43 Å^2^), also caused hemolysis, which may be related to the increased number of hydrogen bond acceptors (HBA 3). In contrast, non-hemolytic derivatives, such as **2**, **3**, **7**, **8**, **9**, and **10**, displayed balanced physicochemical parameters, with moderate lipophilicity (logP ≈ 2.1–2.7) and good solubility (LogS > −4.0), which likely limited their interaction with the lipid bilayer and explains their low hemolytic activity.

For comparison, the reference ligands presented in Table 2 (melatonin, febuxostat, and indomethacin), whose hemolytic activity was not experimentally tested, exhibit molecular descriptors consistent with their known pharmacological properties. Melatonin, with low molecular weight, moderate lipophilicity, and appreciable TPSA, is unlikely to disrupt the molecular structure of RBCs membranes. In contrast, febuxostat, with higher lipophilicity and low TPSA (11.45 Å^2^), may interact more readily with erythrocyte membrane components, suggesting higher hemolytic potential. Indomethacin, due to its combination of high lipophilicity and moderate polarity, exhibits potentially low hemolytic activity.

In summary, our results indicate that lipophilicity, polarity, and solubility are key factors determining the hemolytic activity of new derivatives. Compounds with higher logP values and moderate solubility are more likely to disrupt the molecular structure of RBCs membranes, whereas derivatives with balanced physicochemical profiles remain non-hemolytic. Combining in silico calculated physicochemical parameters with experimentally obtained hemolysis data allows prediction and interpretation of the cell membrane activity of a newly synthesized compound.

## 3. Materials and Methods

### 3.1. Instrumentation and Chemicals

All starting materials were obtained from Sigma-Aldrich and were used without purification. The ^1^H and ^13^C NMR spectra were obtained using a Varian spectrometer (Palo Alto, CA, USA), VNMR-S 400 MHz (DMSO-*d_6_* as the solvent and TMS as the internal standard). FTIR spectra were recorded on a Nicolet iS5 Spectrometer (KBr pellets) (Thermo Fisher Scientific, Warszawa, Poland). EI mass spectra were measured on a 320-MS/450 GC mass spectrometer (Bruker, Poznan, Poland). The nitrogen, carbon, and hydrogen content was determined through elemental analysis using the Elemental Analyzer Vario EL III apparatus (Shimadzu, Kyoto, Japan). Melting points were determined with an SMP-20 apparatus (BŰCHI Labortechnik AG, Essen, Germany). Analytical thin-layer chromatography (TLC) was carried out on silica gel plates 60 F254 (Merck, Darmstadt, Germany). The detection of TLC was performed using UV light.

### 3.2. Synthesis of Indole Derivatives

The general synthesis procedure for **2**-**16**

One mmol of gramine and 2 mmol of the following pyrazole and its derivatives [pyrazole (**2**), 4-methylpyrazole (**3**), 4-ethylpyrazole (**4**), 4-isopropylpyrazole (**5**), 5-isopropylpyrazole (**6**), 3,5-dimethylpyrazole (**7**), 4-ethynylpyrazole (**8**) 5-methoxypyrazole (**9**) 4-fluoropyrazole (**10**), 4-chloropyrazole (**11**), 4-bromopyrazole (**12**), 4-iodopyrazole (**13**), 4-bromo-3-methylpyrazole (**14**, **15**), 3-bromo-5-methyl-1H-pyrazole (**16**)] were diluted in 12 mL of toluene and heated under reflux for 9–20 h. The reaction was monitored by thin-layer chromatography (chloroform: methanol, 5:1), and when complete, the solution was poured into a small beaker to evaporate in the air. The obtained crystals or solids were filtered, and compounds **8**, **10**, **11**, **15**, and **16** required column chromatography to purify (ethyl acetate—8; toluene/acetone 50:1—10, 11) or separate (toluene/acetone 50:1—15, 16) the products. Crystals **2**–**7**, **10**–**13**, **16**, and **17** were obtained from toluene after slow evaporation in the air.

3-((1H-pyrazol-1-yl)methyl)-1H-indole (**2**)

Orange crystals (135 mg, 68%); m.p. 113–115 °C; ^1^H NMR (400 MHz, DMSO-*d_6_*): δ 11.09 (s, 1H), 7.68 (dd, *J* = 2.3, 0.7 Hz, 1H), 7.52 (ddt, *J* = 8.0, 1.3, 0.7 Hz, 1H), 7.44—7.39 (m, 2H), 7.37 (dt, *J* = 8.1, 0.9 Hz, 1H), 7.08 (ddd, *J* = 8.1, 7.0, 1.2 Hz, 1H), 6.97 (ddd, *J* = 8.0, 7.0, 1.0 Hz, 1H), 6.18 (t, *J* = 2.0 Hz, 1H), 5.44 (d, *J* = 0.7 Hz, 2H); ^13^C NMR (101 MHz, DMSO-*d_6_*): δ 138.1, 136.3, 129.1, 126.4, 124.9, 121.3, 118.9, 118.6, 111.6, 110.5, 105.0, 46.8; IR (KBr): 3422, 3183, 1621, 1513, 1443, 1235, 1090, 756, 617, 583 cm^−1^; EI-MS (*m*/*z*, % int.): 130 (indole, 100), 197 (M^+^, 71). Analysis calculated for C_12_H_11_N_3_: C, 73.07; H, 5.62; N, 21.30; found: C, 73.31; H, 6.01; N, 20.81.

3-((4-methyl-1H-pyrazol-1-yl)methyl)-1H-indole (**3**)

Colorless crystals, (153 mg, 72%); mp 114–116 °C; ^1^H NMR (400 MHz, DMSO-*d_6_*): δ 11.07 (s, 1H), 7.52 (ddt, *J* = 7.9, 1.4, 0.7 Hz, 1H), 7.41—7.39 (m, 2H), 7.37 (dt, *J* = 8.1, 1.0 Hz, 1H), 7.19 (t, *J* = 0.7 Hz, 1H), 7.08 (ddd, *J* = 8.2, 7.0, 1.2 Hz, 1H), 6.97 (ddd, *J* = 8.0, 7.0, 1.0 Hz, 1H), 5.35 (d, *J* = 0.7 Hz, 2H), 2.02—1.91 (m, 3H); ^13^C NMR (101 MHz, DMSO-*d_6_*): δ 138.3, 136.3, 127.7, 126.4, 124.9, 121.3, 118.9, 118.6, 114.7, 111.5, 110.6, 46.8, 8.7; IR (KBr): 3414, 3177, 2933, 1617, 1444, 1343, 1242, 1006, 744, 642, 610, 590 cm^−1^; EI-MS (*m*/*z*, % int.): 130 (indole, 100), 211 (M^+^, 35). Analysis calculated for C_13_H_13_N_3_: C, 73.91; H, 6.20; N, 19.89; found: C, 73.41; H, 6.69; N, 19.62.

3-((4-ethyl-1H-pyrazol-1-yl)methyl)-1H-indole (**4**)

White crystals, (192 mg, 85%); mp 118–120 °C; ^1^H NMR (400 MHz, DMSO-*d_6_*): δ 11.08 (s, 1H), 7.53 (ddt, *J* = 7.9, 1.4, 0.7 Hz, 1H), 7.43 (q, *J* = 0.8 Hz, 1H), 7.41 (d, *J* = 2.4 Hz, 1H), 7.37 (dt, *J* = 8.1, 0.9 Hz, 1H), 7.23 (d, *J* = 0.8 Hz, 1H), 7.08 (ddd, *J* = 8.2, 7.0, 1.2 Hz, 1H), 6.97 (ddd, *J* = 8.0, 7.0, 1.1 Hz, 1H), 5.36 (d, *J* = 0.7 Hz, 2H), 2.42—2.31 (m, 2H), 1.07 (t, *J* = 7.6 Hz, 3H); ^13^C NMR (101 MHz, DMSO-*d_6_*): δ 137.0, 136.3, 126.6, 126.4, 124.9, 122.3, 121.3, 118.8, 118.6, 111.5, 110.6, 46.8, 16.9, 15.3; IR (KBr): 3166, 2926, 1621, 1459, 1343, 1168, 1006, 744, 614, 592, 578, cm^−1^; EI-MS (*m*/*z*, % int.): 130 (indole, 100), 225 (M^+^, 19). Analysis calculated for C_14_H_15_N_3_: C, 74.64; H, 6.71; N, 18.65; found: C, 74.56; H, 6.54; N, 18.45.

3-((4-isopropyl-1H-pyrazol-1-yl)methyl)-1H-indole (**5**)

White crystals, (178 mg, 74%); mp 104–106 °C; ^1^H NMR (400 MHz, DMSO-*d_6_*): δ 11.08 (s, 1H), 7.55 (ddt, *J* = 7.8, 1.2, 0.7 Hz, 1H), 7.45 (d, *J* = 0.9 Hz, 1H), 7.41 (d, *J* = 2.5 Hz, 1H), 7.37 (dt, *J* = 8.1, 0.9 Hz, 1H), 7.25 (t, *J* = 0.7 Hz, 1H), 7.08 (ddd, *J* = 8.1, 7.0, 1.2 Hz, 1H), 6.98 (ddd, *J* = 8.0, 7.0, 1.0 Hz, 1H), 5.36 (d, *J* = 0.7 Hz, 2H), 2.72 (hept, *J* = 6.8 Hz, 1H), 1.10 (d, *J* = 6.8 Hz, 6H); ^13^C NMR (101 MHz, DMSO-*d_6_*): δ 136.2, 135.9, 128.0, 126.5, 125.7, 124.9, 121.3, 118.9, 118.6, 111.5, 110.7, 46.8, 23.9, 23.8; IR (KBr): 3174, 1622, 1457, 1290, 1072, 746, 624, 586 cm^−1^; EI-MS (*m*/*z*, % int.): 130 (indole, 100), 239 (M^+^, 37). Analysis calculated for C_15_H_17_N_3_: C, 75.28; H, 7.16; N, 17.56; found: C, 75.55; H, 7.07; N, 17.84.

3-((5-isopropyl-1H-pyrazol-1-yl)methyl)-1H-indole (**6**)

White crystals, (92 mg, 38%); mp 150–153 °C; ^1^H NMR (400 MHz, DMSO-*d_6_*): δ 11.01 (s, 1H), 7.55 (d, *J* = 7.9 Hz, 1H), 7.34 (dt, *J* = 8.3, 1.0 Hz, 1H), 7.32 (d, *J* = 1.9 Hz, 1H), 7.27 (d, *J* = 2.4 Hz, 1H), 7.06 (ddd, *J* = 8.1, 7.0, 1.2 Hz, 1H), 6.95 (ddd, *J* = 8.0, 7.0, 1.0 Hz, 1H), 6.02 (d, *J* = 1.8 Hz, 1H), 5.43 (d, *J* = 0.8 Hz, 2H), 3.16 (hept, *J* = 6.7 Hz, 1H), 1.08 (d, *J* = 6.8 Hz, 6H); ^13^C NMR (101 MHz, DMSO-*d_6_*): δ 148.6, 137.2, 136.2, 126.3, 124.0, 121.2, 118.98, 118.7, 111.4, 111.1, 101.5, 101.5, 44.9, 24.5, 22.8; IR (KBr): 3421, 3145, 2972, 2929, 1620, 155, 1455, 1409, 1086, 744, 634, 604, 580 cm^−1^; EI-MS (*m*/*z*, % int.): 130 (indole, 100), 239 (M^+^, 20). Analysis calculated for C_15_H_17_N_3_: C, 75.28; H, 7.16; N, 17.56; found: C, 75.23; H, 7.17; N, 17.92.

3-((3,5-dimethyl-1H-pyrazol-1-yl)methyl)-1H-indole (**7**)

White crystals, (177 mg, 79%); mp 135–136 °C; ^1^H NMR (400 MHz, DMSO-*d_6_*): δ 11.01 (s, 1H), 7.59—7.52 (m, 1H), 7.35 (dt, *J* = 8.2, 0.9 Hz, 1H), 7.27 (d, *J* = 2.4 Hz, 1H), 7.07 (ddd, *J* = 8.2, 7.0, 1.2 Hz, 1H), 6.96 (ddd, *J* = 8.0, 7.0, 1.0 Hz, 1H), 5.75 (s, 1H), 5.27 (d, *J* = 0.8 Hz, 2H), 2.19 (d, *J* = 0.8 Hz, 3H), 2.08 (s, 3H); ^13^C NMR (101 MHz, DMSO-*d_6_*): δ 145.0, 138.1, 136.2, 126.3, 124.1, 121.2, 118.8, 118.7, 111.4, 110.9, 104.7, 44.3, 13.4, 10.9; IR (KBr): 3421, 3166, 2922, 1624, 1546, 1459, 1277, 1029, 738, 605, 583 cm^−1^; EI-MS (*m*/*z*, % int.): 130 (indole, 100), 225 (M^+^, 16). Analysis calculated for C_14_H_15_N_3_: C, 74.64; H, 6.71; N, 18.55; found: C, 74.61; H, 6.68; N, 18.93.

3-((4-ethynyl-1H-pyrazol-1-yl)methyl)-1H-indole (**8**)

Light brown solid, (27 mg, 12%); mp 87–90 °C; ^1^H NMR (400 MHz, DMSO-*d_6_*): δ 11.13 (s, 1H), 8.02 (d, *J* = 0.8 Hz, 1H), 7.59 (d, *J* = 0.7 Hz, 1H), 7.54 (ddt, *J* = 7.9, 1.4, 0.7 Hz, 1H), 7.44 (d, *J* = 2.5 Hz, 1H), 7.38 (dt, *J* = 8.1, 1.0 Hz, 1H), 7.04 (s, 0H), 6.99 (ddd, *J* = 8.0, 7.0, 1.0 Hz, 1H), 5.43 (d, *J* = 0.8 Hz, 2H), 3.93 (s, 1H); ^13^C NMR (101 MHz, DMSO-*d_6_*): δ 141.1, 136.2, 132.7, 126.2, 125.2, 121.4, 118.9, 118.4, 111.6, 109.7, 100.9, 80.8, 75.8, 47.2; IR (KBr): 3283, 2117, 1621, 1437, 1374, 1349, 1110, 742, 630, 605, 586 cm^−1^; EI-MS (*m*/*z*, % int.): 129 (indole, 67), 221 (M^+^, 97). Analysis calculated for C_14_H_11_N_3_: C, 76.00; H, 5.01; N, 18.99; found: C, 75.76; H, 5.24; N, 19.17.

3-((5-methoxy-1H-pyrazol-1-yl)methyl)-1H-indole (**9**)

Orange crystals, (114 mg, 50%); mp 163–165 °C; ^1^H NMR (400 MHz, DMSO-*d_6_*): δ 11.00 (s, 1H), 7.58 (ddt, *J* = 7.9, 1.3, 0.7 Hz, 1H), 7.34 (dt, *J* = 8.1, 1.0 Hz, 1H), 7.27 (d, *J* = 2.5 Hz, 1H), 7.20 (d, *J* = 2.0 Hz, 1H), 7.07 (ddd, *J* = 8.2, 7.0, 1.2 Hz, 1H), 6.97 (ddd, *J* = 8.0, 7.0, 1.1 Hz, 1H), 5.61 (d, *J* = 2.0 Hz, 1H), 5.18 (d, *J* = 0.8 Hz, 2H), 3.86 (s, 3H); ^13^C NMR (101 MHz, DMSO-*d_6_*): δ 154.5, 137.1, 136.1, 126.3, 124.5, 121.2, 118.7, 118.6, 111.5, 110.7, 84.4, 58.8, 41.9; IR (KBr): 3424, 2828, 1621, 1553, 1417, 1238, 1114, 736, 631, 610, 582 cm^−1^; EI-MS (*m*/*z*, % int.): 130 (indole, 100), 227 (M^+^, 32). Analysis calculated for C_14_H_11_N_3_: C, 68.70; H, 5.77; N, 18.49; found: C, 68.84; H, 5.84; N, 18.45.

3-((4-fluoro-1H-pyrazol-1-yl)methyl)-1H-indole (**10**)

Orange crystals, (50 mg, 23%); mp 102–104 °C; ^1^H NMR (400 MHz, DMSO-*d_6_*): δ 11.12 (s, 1H), 7.81 (dd, *J* = 4.7, 0.9 Hz, 1H), 7.52 (ddt, *J* = 7.9, 1.3, 0.7 Hz, 1H), 7.44 (d, *J* = 2.5 Hz, 1H), 7.42 (dd, *J* = 4.4, 0.9 Hz, 1H), 7.38 (dt, *J* = 8.1, 0.9 Hz, 1H), 7.09 (ddd, *J* = 8.1, 7.0, 1.2 Hz, 1H), 6.99 (ddd, *J* = 8.0, 7.0, 1.1 Hz, 1H), 5.36 (d, *J* = 0.8 Hz, 2H); ^13^C NMR (101 MHz, DMSO-*d_6_*): δ 150.1, 147.7, 136.3, 126.3, 125.2, 124.9, 124.8, 121.4, 118.9, 118.5, 115.8, 115.5, 111.6, 109.9, 48.1; IR (KBr): 3416, 3228, 3127, 1622, 1573, 1418, 1353, 1107, 744, 637, 609, 581 cm^−1^; EI-MS (*m*/*z*, % int.): 130 (indole, 100), 215 (M^+^, 16). Analysis calculated for C_12_H_10_FN_3_: C, 66.97; H, 4.68; N, 19.52; found: C, 66.87; H, 4.81; N, 19.88.

3-((4-chloro-1H-pyrazol-1-yl)methyl)-1H-indole (**11**)

Light yellow crystals, (77 mg, 33%); mp 118–120 °C; ^1^H NMR (400 MHz, DMSO-*d_6_*): δ 11.14 (s, 1H), 7.93 (d, *J* = 0.8 Hz, 1H), 7.54 (d, *J* = 7.8 Hz, 1H), 7.49 (d, *J* = 0.8 Hz, 1H), 7.45 (d, *J* = 2.4 Hz, 1H), 7.38 (dt, *J* = 8.2, 0.9 Hz, 1H), 7.10 (ddd, *J* = 8.2, 7.0, 1.2 Hz, 1H), 7.00 (ddd, *J* = 8.0, 7.0, 1.1 Hz, 1H), 5.42 (s, 2H); ^13^C NMR (101 MHz, DMSO-*d_6_*): δ 136.5, 136.3, 127.5, 126.3, 125.3, 121.4, 119.0, 118.4, 111.6, 109.8, 107.8, 47.8; IR (KBr): 3411, 1620, 1457, 1295, 1097, 973, 744, 647, 607 cm^−1^; EI-MS (*m*/*z*, % int.): 130 (indole, 56), 231 (M^+^, 100). Analysis calculated for C_12_H_10_ClN_3_: C, 62.21; H, 4.45; N, 18.14; found: C, 62.16; H, 4.65; N, 17.91.

3-((4-bromo-1H-pyrazol-1-yl)methyl)-1H-indole (**12**)

Yellow crystals, (110 mg, 40%); mp 122–125 °C; ^1^H NMR (400 MHz, DMSO-*d_6_*): δ 11.14 (s, 1H), 7.93 (d, *J* = 0.8 Hz, 1H), 7.54 (dq, *J* = 7.9, 1.0 Hz, 1H), 7.50 (d, *J* = 0.8 Hz, 1H), 7.45 (d, *J* = 2.5 Hz, 1H), 7.38 (dt, *J* = 8.2, 0.9 Hz, 1H), 7.10 (ddd, *J* = 8.2, 7.0, 1.2 Hz, 1H), 7.00 (ddd, *J* = 8.0, 7.0, 1.0 Hz, 1H), 5.46—5.41 (m, 2H); ^13^C NMR (101 MHz, DMSO-*d_6_*): δ 138.6, 136.3, 129.5, 126.3, 125.4, 121.4, 119.0, 118.4, 111.6, 109.8, 91.5, 47.7; IR (KBr): 3278, 3136, 1454, 1297, 1102, 743, 645, 607 cm^−1^; EI-MS (*m*/*z*, % int.): 130 (indole, 63), 275 (M^+^, 100). Analysis calculated for C_12_H_10_BrN_3_: C, 52.20; H, 3.65; N, 15.11; found: C, 52.39; H, 4.02; N, 15.36.

3-((4-iodo-1H-pyrazol-1-yl)methyl)-1H-indole (**13**)

Yellow crystals, (126 mg, 39%); mp 102–104 °C; ^1^H NMR (400 MHz, DMSO-*d_6_*): δ 11.13 (s, 1H), 7.87 (d, *J* = 0.7 Hz, 1H), 7.56—7.52 (m, 1H), 7.49 (d, *J* = 0.7 Hz, 1H), 7.45 (d, *J* = 2.5 Hz, 1H), 7.37 (dt, *J* = 8.1, 0.9 Hz, 1H), 7.09 (ddd, *J* = 8.2, 7.0, 1.2 Hz, 1H), 6.99 (ddd, *J* = 8.0, 7.0, 1.0 Hz, 1H), 5.45 (s, 2H); ^13^C NMR (101 MHz, DMSO-*d_6_*): δ 143.0, 136.2, 133.5, 126.2, 125.3, 121.4, 118.9, 118.4, 111.6, 109.9, 56.5, 56.5, 47.3; IR (KBr): 3421, 3220, 1622, 1456, 1356, 1239, 997, 939, 746, 615, 602, 567 cm^−1^; EI-MS (*m*/*z*, % int.): 38 (C_2_N^+^, 100), 129 (indole, 19), 323 (M^+^, 16). Analysis calculated for C_12_H_10_IN_3_: C, 44.60; H, 3.12; N, 13.00; found: C, 44.80; H, 3.27; N, 13.03.

3-((4-bromo-5-methyl-1H-pyrazol-1-yl)methyl)-1H-indole (**14**)

White crystals, (56 mg, 19%); mp 148–150 °C; ^1^H NMR (400 MHz, DMSO-*d_6_*): δ 11.09 (s, 1H), 7.56 (d, *J* = 7.9 Hz, 1H), 7.48 (s, 1H), 7.39 (d, *J* = 2.5 Hz, 1H), 7.38—7.34 (m, 1H), 7.11—7.05 (m, 1H), 7.00—6.94 (m, 1H), 5.45 (s, 2H), 2.23 (s, 3H); ^13^C NMR (101 MHz, DMSO-*d_6_*): δ 137.1, 136.2, 136.1, 126.2, 124.5, 121.3, 118.8, 118.6, 111.4, 109.8, 92.1, 46.3, 9.4; IR (KBr): 3174, 1621, 1441, 1236, 944, 736, 635, 613, 582 cm^−1^ EI-MS (*m*/*z*, % int.): 130 (indole, 100), 289 (M^+^, 17). Analysis calculated for C_13_H_12_BrN_3_: C, 53.81; H, 4.17; N, 14.48; found: C, 53.90; H, 4.61; N, 14.20.

3-((4-bromo-3-methyl-1H-pyrazol-1-yl)methyl)-1H-indole (**15**)

White crystals, (100 mg, 35%); mp 149–151 °C; ^1^H NMR (400 MHz, DMSO-*d_6_*): δ 11.12 (s, 1H), 7.84 (s, 1H), 7.54 (ddt, *J* = 7.9, 1.3, 0.7 Hz, 1H), 7.44 (d, *J* = 2.5 Hz, 1H), 7.38 (dt, *J* = 8.1, 0.9 Hz, 1H), 7.09 (ddd, *J* = 8.2, 7.0, 1.2 Hz, 1H), 7.02—6.97 (m, 1H), 5.35 (s, 2H), 2.08 (s, 3H) ^13^C NMR (101 MHz, DMSO-*d_6_*): δ 145.1, 136.2, 129.7, 126.2, 125.3, 121.3, 118.9, 118.4, 111.6, 109.8, 91.8, 47.3, 11.5; IR (KBr): 3222, 1621, 1327, 1144, 747, 603, 584 cm^−1^; EI-MS (*m*/*z*, % int.): 129 (indole, 100), 289 (M^+^, 66). Analysis calculated for C_13_H_12_BrN_3_: C, 53.81; H, 4.17; N, 14.48; found: C, 54.01; H, 4.67; N, 14.20.

3-((3-bromo-5-methyl-1H-pyrazol-1-yl)methyl)-1H-indole (**16**)

Brown crystals, (71 mg, 25%); mp 144–147 °C; ^1^H NMR (400 MHz, DMSO-*d_6_*): 11.09 (s, 1H), 7.61 (ddt, *J* = 7.9, 1.3, 0.7 Hz, 1H), 7.36 (dt, *J* = 8.1, 1.0 Hz, 1H), 7.34 (d, *J* = 2.5 Hz, 1H), 7.08 (ddd, *J* = 8.1, 7.0, 1.2 Hz, 1H), 6.99 (ddd, *J* = 8.0, 7.0, 1.1 Hz, 1H), 6.19 (d, *J* = 0.6 Hz, 1H), 5.38 (d, *J* = 0.7 Hz, 2H), 2.12 (d, *J* = 0.5 Hz, 3H); ^13^C NMR (101 MHz, DMSO-*d_6_*): δ 148.0, 136.2, 126.2, 124.9, 121.3, 118.9, 118.7, 111.6, 110.0, 107.6, 107.6, 45.4, 13.7; IR (KBr): 3398, 1707, 1624, 1474, 1191, 751, 594 cm^−1^; EI-MS (*m*/*z*, % int.): 130 (indole, 100), 289 (M^+^, 28). Analysis calculated for C_13_H_12_BrN_3_: C, 53.81; H, 4.17; N, 14.48; found: C, 54.11; H, 4.65; N, 14.13.

Synthesis of **18**:

5-Nitroindazole (2 mmol) was dissolved in 12 mL of DMF, and K_2_CO_3_ (1.5 mmol) was added, followed by stirring for 1 h. Subsequently, a gramine solution (1 mmol) in 8 mL of toluene was added, and the mixture was heated under reflux for 15 h. The reaction was monitored by thin-layer chromatography (toluene:acetone 5:1). Then, 10 mL of water was added, and the mixture was extracted with ethyl acetate, washed with water and brine, dried over Na_2_SO_4_, and evaporated. The resulting product was purified using column chromatography (toluene:acetone 50:1).

1-((1H-indol-3-yl)methyl)-5-nitro-1H-indazole (**18**)

Light yellow solid (44 mg, 14%); m.p. 225–227 °C; ^1^H NMR (400 MHz, DMSO-*d_6_*): δ 11.13 (s, 1H), 8.79 (t, *J* = 2.6 Hz, 1H), 8.42—8.37 (m, 1H), 8.20 (dt, *J* = 9.2, 2.5 Hz, 1H), 8.07—7.99 (m, 1H), 7.60 (t, *J* = 2.7 Hz, 1H), 7.54 (d, *J* = 8.0 Hz, 1H), 7.38—7.31 (m, 1H), 7.10—7.01 (m, 1H), 6.94 (t, *J* = 7.5 Hz, 1H), 5.87 (d, *J* = 2.7 Hz, 2H); ^13^C NMR (101 MHz, DMSO-*d_6_*): δ 141.5, 140.5, 136.3, 135.7, 126.2, 125.2, 122.9, 121.4, 120.9, 119.1, 119.1, 118.9, 118.6, 111.6, 110.8, 109.8, 45.0; IR (KBr): 3289, 1616, 1516, 1490, 1340, 857, 754, 632, 598, 579 cm^−1^; EI-MS (*m*/*z*, % int.): 130 (indole, 100), 292 (M^+^, 75). Analysis calculated for C_12_H_12_N_4_: C, 65.75; H,4.14; N, 19.17; found: C, 65.61; H, 4.26; N, 19.47.

### 3.3. X-Ray Data Collection and Refinement of the Structures

Suitable single crystals of compounds **2**–**7**, **10**–**13**, and **16** were chosen for X-ray diffraction measurements carried out at various temperatures on a Bruker D8 QUEST KAPPA diffractometer with a microfocus sealed tube, using a multilayer mirror as a monochromator and a Bruker PHOTON III CPAD detector and on Xcalibur kappa-geometry diffractometer equipped with Eos CCD detector (see Table S1 for details of experimental conditions). The obtained intensity data were processed with the SAINT V8.41 [54] and CrysAlis PRO [55] programs, respectively. The structures were solved using SHELXT [56] and refined by the full-matrix least-squares method against F^2^ by SHELXL [57]. Non-hydrogen atoms were refined with anisotropic displacement parameters except for the bromine and the methyl carbon in the structure of **16**, which were refined isotropically due to their very low site occupancy factor of only 0.08. Methyl hydrogens with the site occupancy of only 8% were not located. All other hydrogen atoms were placed at idealized positions and refined as riding on their carrier atoms. Isotropic displacement parameters for H-atoms were given a value 20% higher than the isotropic equivalent for the atom to which the H atom was bonded, except for the methyl hydrogens, for which this value was 50%. Residual electron densities around the C3-methyl substituent in **16** have been modeled as originating from the bromide substituent, representing the 3-Br, 5-Me tautomer 17, which occupies the same crystallographic site as the dominant 5-Br, 3-Me isomer. The 5(3) and 3(5) molecules are disordered as a solid solution with occupancies 0.92:0.08. Graphical images and data presented in this article were generated by Mercury [58]. CCDC contains the supplementary crystallographic data for **2** (Deposition Number 2425151), **3** (Deposition Number 2425152), **4** (Deposition Number 2425153), **5** (Deposition Number 2425154), **6** (Deposition Number 2425155), **7** (Deposition Number 2425156), **10** (Deposition Number 2425157), **11** (Deposition Number 2425158), **12** (Deposition Number 2425159), **13** (Deposition Number 2425160), and **16** (Deposition Number 2425161). These data can be obtained free of charge via https://www.ccdc.cam.ac.uk/ (accessed on 19 February 2025) (or from the CCDC, 12 Union Road, Cambridge CB2 1EZ, UK; Fax: +44-1223-336033; email: deposit@ccdc.cam.ac.uk).

### 3.4. Biological Study

#### 3.4.1. Human Red Blood Cells Preparation

All methods were carried out in accordance with the relevant guidelines and regulations, and the Bioethics Committee approved all experimental protocols for scientific research at the Medical University of Poznań (agreement no. ZP/2867/D/21). Human red blood cells (RBCs) concentrates were purchased from the Blood Bank in Poznań without any contact with blood donors. Informed consent was obtained from all blood donors.

Freshly human RBCs suspensions (hematocrit 65%) were washed three times (3000 rpm, 10 min, +4 °C) in 7.4 pH phosphate-buffered saline (PBS—137 mM NaCl, 2.7 mM KCl, 10 mM Na_2_HPO_4_, 1.76 mM KH_2_PO_4_) supplemented with 10 mM glucose. After washing, the cells were suspended in PBS buffer at a concentration of 1.65 × 10^9^ cells/mL (hematocrit ~15%), stored at 4 °C, and used within 5 h.

#### 3.4.2. Hemolytic Assay

Hemolytic activity was evaluated as previously described [59]. Briefly, RBCs (1.65 x 10^8^ cells/mL, hematocrit 1.5%) were incubated in PBS (pH 7.4) supplemented with 10 mM glucose and containing the tested compounds at a concentration of 0.1 mg/mL for 60 min at 37 °C in a shaking incubator. The concentration of derivatives used in this study was selected according to our previous research [24]. Negative control samples consisted of RBCs incubated in PBS without tested compounds, whereas positive control samples consisted of RBCs incubated in ice-cold deionized water. After incubation, the RBCs suspensions were centrifuged (3000 rpm, 10 min, +4 °C), and the degree of hemolysis was estimated by measuring the absorbance (Ab) of the supernatant at λ = 540 nm using a BioMate™ 160 UV-Vis spectrophotometer. The percentage of hemolysis was calculated according to Equation (1):

Hemolytic activity (%) = (Ab_comp_/Ab_contr_) × 100, (1)

where Ab_comp_ is the absorbance of the supernatant the tested compound, and Ab_contr_ is the absorbance of the supernatant of the positive control.

A hemolysis degree below 10% was considered indicative no hemolytic activity. Each sample was prepared in triplicate, and the experiments were independently repeated with RBCs obtained from different donors. Results are presented as mean values ± standard deviation (SD) (n = 9).

#### 3.4.3. Inhibition of Oxidative Stress-Induced Hemolysis

The cytoprotective activity of the derivatives was evaluated using the previously described [24]. Briefly, RBCs (1.65 × 10^8^ cells/mL, 1.5% hematocrit) were preincubated in PBS (pH 7.4) supplemented with 10 mM glucose and containing either the tested compound or Trolox (used as the reference antioxidant) at a concentration of 0.1 mg/mL for 20 min at 37 °C in a shaking incubator. The concentration of derivatives was selected according to our previous studies [24]. After preincubation, 2,2′-Azobis(2-amidinopropane)dihydrochloride (AAPH) was added to a final concentration of 60 mM, and the samples were incubated for an additional four hours. RBCs incubated in PBS or in PBS with AAPH were used as the negative and positive controls, respectively. After incubation, the RBCs suspensions were centrifuged (3000 rpm, 5 min, +4 °C), and the degree of oxidative stress- induced hemolysis was determined by measuring the absorbance (Ab) of the supernatant at λ = 540 nm using a BioMate™ 160 UV-Vis spectrophotometer. The percentage of ROS-induced hemolysis inhibition was calculated according to Equation (2):

Inhibition of hemolysis (%) = 100 − [(Ab_comp+AAPH_ − Ab_PBS_/Ab_AAPH_ − Ab_PBS_) × 100], (2)

where Ab_comp+AAPH_ is the absorbance of the supernatant from samples incubated with the compound tested in the presence of AAPH, Ab_PBS_ is the absorbance of the negative control, and Ab_AAPH_ is the absorbance of the supernatant of the positive control. Each sample was prepared in triplicate, and the experiments were independently repeated with RBCs obtained from different donors. Results are presented as mean values ± standard deviation (SD) (n = 9).

#### 3.4.4. Statistical Analysis

For antioxidant and cytoprotective properties, data were plotted as the mean value ± standard deviation (SD) of the results of three independent experiments, with each sample analyzed in triplicate (n = 9). A paired t-Student test was used to compare the antioxidant activity of derivatives with the activity of the standard antioxidant, Trolox. Statistical significance was defined as *p* < 0.05. Inactive compounds were indicated as na. Non-statistically significant difference was indicated as n.s.

### 3.5. Molecular Docking—Experimental

The molecular docking process began by converting the SMILES representation of the given chemical structures into 3D structures. This conversion was achieved using the OpenBabel Python library (pybel) (Version 3.0.1) [60] and Biopandas [61]. Next, we employed the standard AutoDock 1.5.7 method [62] to generate protein domains that match PDB IDs 1DNU [63,64], 1N5X [65,66], and 4COX [67,68]. Subsequently, molecular dockings were performed using AutoDock Vina [69]. The specific parameters for each docking search were outlined in Table S2 and determined based on the native ligand coordinates: NAG C 620, TEI A, and IMN A for the 1DNU, 1N5X, and 4COX protein domains, respectively. The depictions were performed using UCSF Chimera 1.16 software [70], and the 2D diagrams and interaction maps were prepared using the ProteinsPlus algorithms [45,71], namely PoseView [45] and PoseEdit [47].

### 3.6. In Silico Study

The physicochemical properties of all compounds tested were evaluated using the SwissADME web server (www.swissadme.ch) (accessed on 14 November 2024).

## 4. Conclusions

A series of indole–pyrazole hybrids was successfully synthesized, and their structures were confirmed by spectroscopic techniques and X-ray analysis. The results indicate that the size, electronic properties, and position of substituents within the pyrazole ring, influence the hemolytic activity of the new compounds. However, size alone is not a decisive factor: crystals **3** (with the 4-methylpyrazole group) and **11** (with the 4-chloropyrazole group) are isostructural, yet they exhibit significantly different hemolytic properties. Our findings further suggest that lipophilicity, polarity, and solubility are key determinants of hemolytic activity of the tested derivatives. Most of the indole–pyrazole hybrids demonstrate notable cytoprotective activity against oxidative stress in human erythrocytes. In particular, the derivatives with an unsubstituted pyrazole ring displayed the most pronounced cytoprotective effect, comparable to that of the reference antioxidant Trolox. These results suggest that indole–pyrazole hybrids are promising candidates for further investigation in a wide range of biomedical applications.

## Figures and Tables

**Table 1:** Torsion angles (°) describe the rotation around the methylene C-C and C-N bonds in the molecules in crystals. Molecular diagrams and perspective views of the molecules are provided to relate the metrical values to a particular molecular shape. The views are consistently along the indole plane, with the N-H bond pointing towards the observer.

** 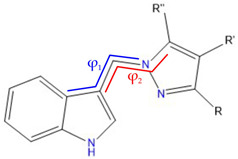 **			
Chemical formula	φ_1_	φ_2_	
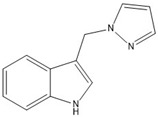 **2**	−53.5(4) 56.5(4)	124.5(3) −124.0(3)	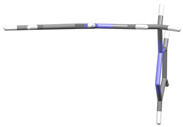 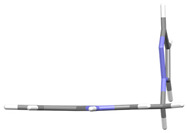
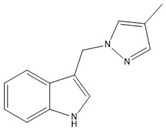 **3**	−75.46(18)	−55.5(2)	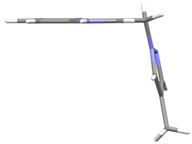
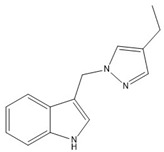 **4**	−67.77(13) −77.64(13)	121.04(11) −43.77(14)	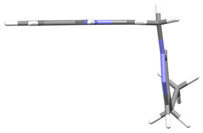 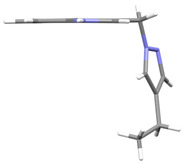
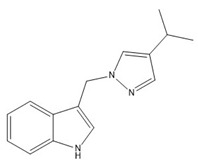 **5**	−74.5(3)	−60.3(3)	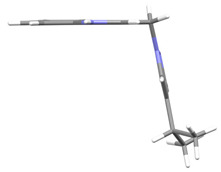
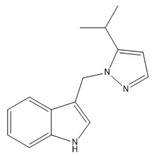 **6**	−68.4(6)	112.6(5)	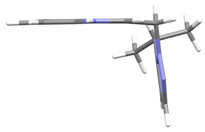
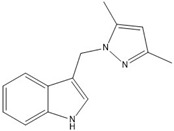 **7**	−60.3(2)	118.9(2)	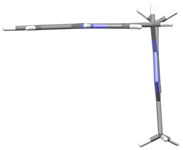
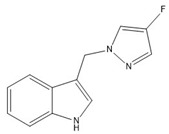 **10**	−72.93(15) −70.32(14)	−41.00(16) −66.78(15)	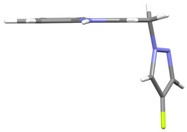 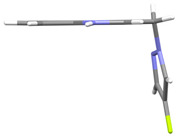
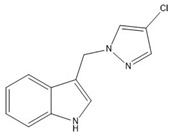 **11**	−75.36(16)	−56.63(19)	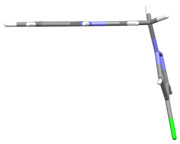
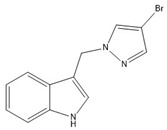 **12**	−70.8(3)	−24.5(4)	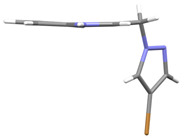
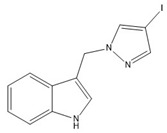 **13**	−73.3(4)	−54.1(4)	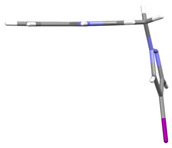
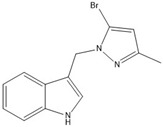 **16**	97.7(3)	97.7(3)	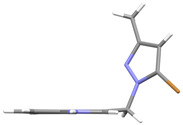
** 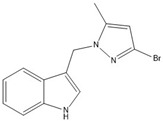 ** **17**	97.7(3)	97.7(3)	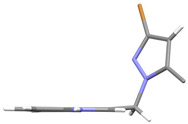

**Table 2:** The molecular descriptors of native ligands. MW—molecular weight [g/mol], RTB—rotatable bonds, HBA—hydrogen bond acceptors, HBD—hydrogen bond donors, TPSA—total polar surface area, logP—an octanol/water partition coefficient. * Solubility class—logS scale: insoluble < −10, poorly < −6, moderately < −4, soluble < −2, and very < 0.

Compound	MW [g/mol]	logP	HBD	HBA	RTB	TPSA [Å^2^]	GI Absorption	BBB Permeant	LogS	Solubility *
Melatonin	232.28	1.83	2	2	5	54.12	High	Yes	−3.10	Soluble
Febuxostan	316.37	3.28	1	5	5	11.45	High	No	−4.95	Moderately
Indomethacin	357.79	3.63	1	4	5	68.53	High	Yes	−5.36	Moderately

**Table 3:** The results of molecular docking to the 1DNU, 1N5X, and 4COX protein domains for all analyzed compounds. Melatonin, febuxostat, and indomethacin were used as reference molecules. The standard deviation of binding energy is calculated based on the nine best poses.

PDB ID	Compound	Average Binding Energy [kcal/mol]	Standard Deviation of Binding Energy [kcal/mol]	Ligand Efficiency [kcal/mol]
1DNU	Melatonin	−5.5	0.09	−0.32
	**2**	−5.6	0.15	−0.37
	**3**	−6.0	0.19	−0.38
	**10**	−5.8	0.18	−0.36
1N5X	Febuxostat	−7.7	0.55	−0.35
	**2**	−8.6	0.76	−0.57
	**3**	−8.9	0.70	−0.56
	**10**	−8.8	0.70	−0.55
4COX	Indomethacin	−9.0	0.27	−0.36
	**2**	−7.7	0.24	−0.51
	**3**	−8.4	0.19	−0.53
	**10**	−8.2	0.16	−0.51

**Table 4:** The predicted hydrogen bonds between the investigated ligands and protein domains.

Compound	Protein Domain	Amino Acid Residues	Distance [Å]	Is it a Compound Donor or Acceptor?
**2**	1N5X	THR 1010 A SER 876 A	2.23 3.21	Donor Acceptor
**3**	1N5X	THR 1010 A SER 876 A	2.25 3.24	Donor Acceptor
**10**	1N5X	THR 1010 A SER 876 A	2.18 3.30	Donor Acceptor
**2**	4COX	TYR 385 A	2.53	Donor
**10**	4COX	TYR 385 A	2.56	Donor

**Table 5:** Physicochemical properties of indole derivatives. MW—molecular weight [g/mol], RTB—rotatable bonds, HBA—hydrogen bond acceptors, HBD—hydrogen bond donors, TPSA—total polar surface area, log P—an octanol/water partition coefficient, GI—gastrointestinal, BBB—blood–brain barrier. LogS is the average value of the logS calculated using three different methods. * Solubility class—logS scale: insoluble < −10, poorly < −6, moderately < −4, soluble < −2, and very < 0.

Compound	MW [g/mol]	logP	HBD	HBA	RTB	TPSA [Å^2^]	GI Absorption	BBB Permeant	LogS	Solubility *
**2**	197.24	1.97	1	1	2	33.61	High	Yes	−3.06	Soluble
**3**	211.26	2.34	1	1	2	33.61	High	Yes	−3.47	Soluble
**4**	225.29	2.64	1	1	3	33.61	High	Yes	−3.84	Soluble
**5**	239.32	2.95	1	1	3	33.61	High	Yes	−4.05	Moderately
**6**	239.32	2.94	1	1	3	33.61	High	Yes	−4.07	Moderately
**7**	225.29	2.67	1	1	2	33.61	High	Yes	−3.85	Soluble
**8**	221.26	2.37	1	1	2	33.61	High	Yes	−3.31	Soluble
**9**	227.26	2.14	1	1	3	42.84	High	Yes	−3.41	Soluble
**10**	215.23	2.32	1	1	2	33.61	High	Yes	−3.29	Soluble
**11**	231.68	2.57	1	1	2	33.61	High	Yes	−3.73	Soluble
**12**	276.13	2.64	1	1	2	33.61	High	Yes	−3.94	Soluble
**13**	323.13	2.67	1	1	2	33.61	High	Yes	−4.03	Moderately
**14**	290.16	2.96	1	1	2	33.61	High	Yes	−4.30	Moderately
**15**	290.16	2.99	1	1	2	33.61	High	Yes	−4.30	Moderately
**16**	290.16	3.02	1	1	2	33.61	High	Yes	−4.49	Moderately
**17**	290.16	3.02	1	1	2	33.61	High	Yes	−4.49	Moderately
**18**	292.29	2.40	1	3	3	79.43	High	No	−4.59	Moderately

## Data Availability

The original contributions presented in this study are included in the article/supplementary material. Further inquiries can be directed to the corresponding authors.

## Supplementary Materials

The following supporting information can be downloaded at: https://www.mdpi.com/article/10.3390/ijms26189018/s1.
