# Supplementary material for: Indole–Pyrazole Hybrids: Synthesis, Structure, and Assessment of Their Hemolytic and Cytoprotective Properties
Source: Int J Mol Sci. 2025 Sep 16;26(18):9018. doi: 10.3390/ijms26189018 (PMC12470152; doi:10.3390/ijms26189018)
Supplement: Supplementary file 1 [file ijms-26-09018-s001.zip › ijms-3821331-supplementary/Supporting information_Indole-pyrazole_rev.pdf]

Supporting Information

Indole-pyrazole hybrids: synthesis, structure, and assessment of their hemolytic and cytoprotective properties

Karolina Babijczuk[1], Klaudia Wawrzyniak[1], Beata Warżajtis[2], Urszula Rychlewska[2], Damian Nowak[3], Yunna da Victoria Banda[4], Lucyna Mrówczyńska*[5] and Beata Jasiewicz*[1]

[1] Department of Bioactive Products, Faculty of Chemistry, Adam Mickiewicz University, Uniwersytetu Poznańskiego 8, 61-614 Poznań, Poland

[2] Department of Crystallography, Faculty of Chemistry, Adam Mickiewicz University, Uniwersytetu Poznańskiego 8, 61-614 Poznań, Poland

[3] Department of Quantum Chemistry, Faculty of Chemistry, Adam Mickiewicz University, Uniwersytetu Poznańskiego 8, 61-614 Poznań, Poland

[4] Department of Biological Science, Faculty of Science, Eduardo Mondlane University, Praça 25 de Junho, 257 C.P. 257 Maputo, Mozambique

[5] Department of Cell Biology, Faculty of Biology, Adam Mickiewicz University, Uniwersytetu Poznańskiego 6, 61-614 Poznań, Poland

* Correspondence: beatakoz@amu.edu.pl (B.J.), lumro@amu.edu.pl (L.M.)

TABLE OF CONTENTS

NMR, MS, and FT-IR spectra of compound **2** S3
NMR, MS, and FT-IR spectra of compound **3** S5
NMR, MS, and FT-IR spectra of compound **4** S7
NMR, MS, and FT-IR spectra of compound **5** S9
NMR, MS, and FT-IR spectra of compound **6** S11
NMR, MS, and FT-IR spectra of compound **7** S13
NMR, MS, and FT-IR spectra of compound **8** S15
NMR, MS, and FT-IR spectra of compound **9** S17
NMR, MS, and FT-IR spectra of compound **10** S19
NMR, MS, and FT-IR spectra of compound **11** S21
NMR, MS, and FT-IR spectra of compound **12** S23
NMR, MS, and FT-IR spectra of compound **13** S25

NMR, MS, and FT-IR spectra of compound **14** S27
NMR, MS, and FT-IR spectra of compound **15** S29
NMR, MS, and FT-IR spectra of compound **16** S31
NMR, MS, and FT-IR spectra of compound **18** S33
Interactions of new derivatives with the 1DNU protein domain.................................S35
Interactions of new derivatives with the 4COX protein domain.................................S36
Table S1..S37
Table S2..S39

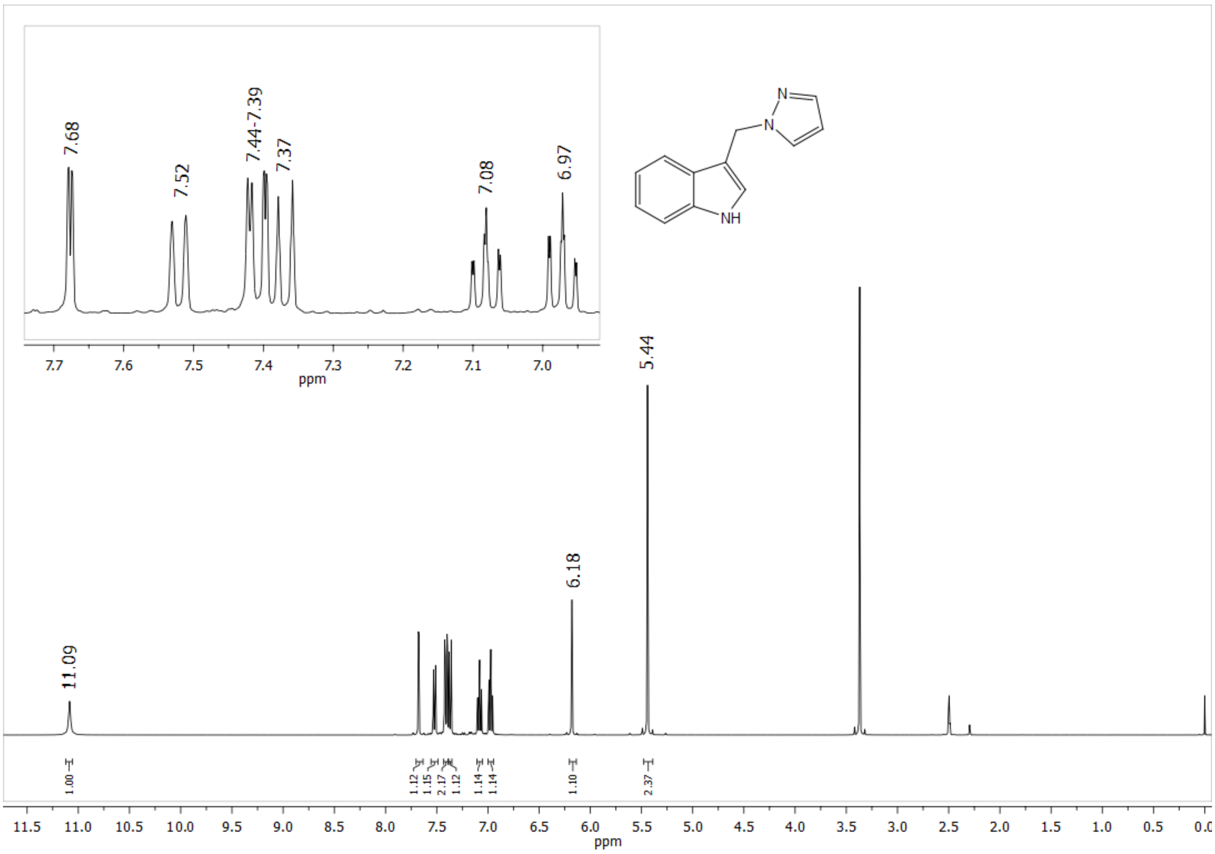

Figure S1a. ^{1}H NMR spectrum of compound **2**

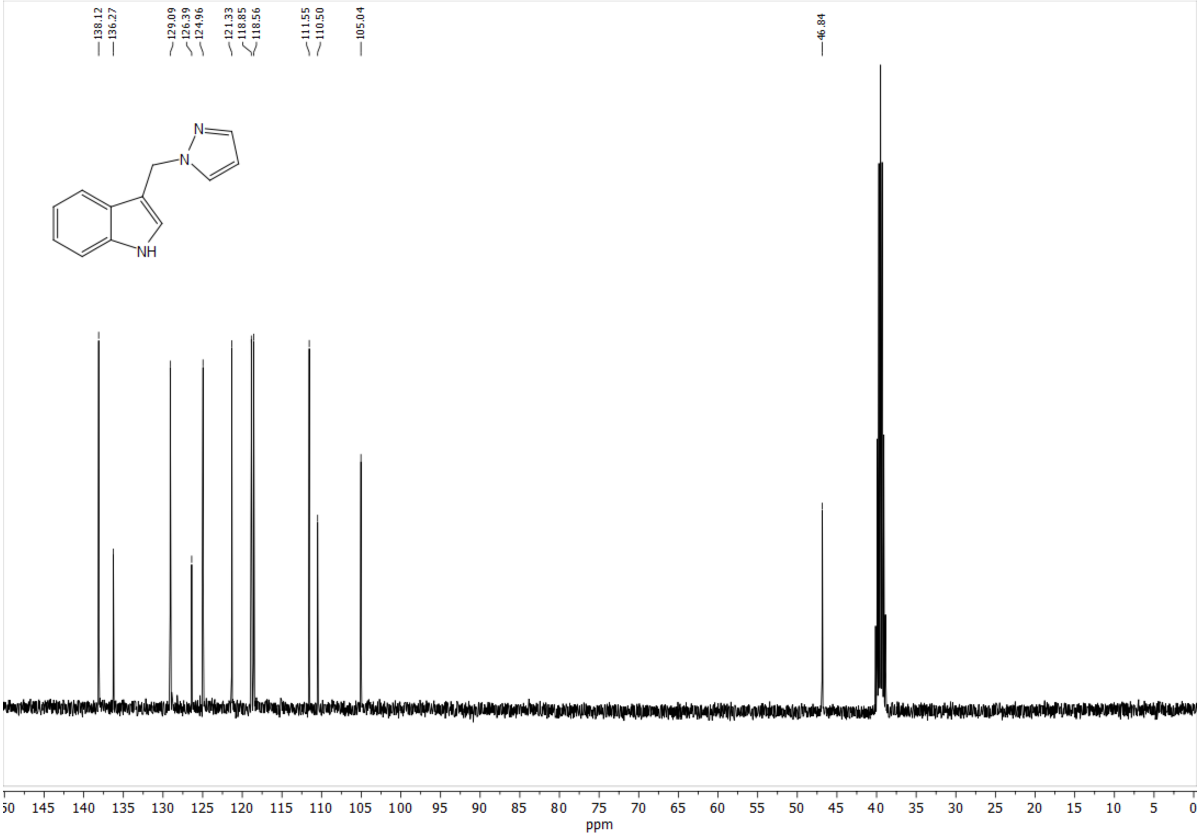

Figure S1b. ^{13}C NMR spectrum of compound **2**

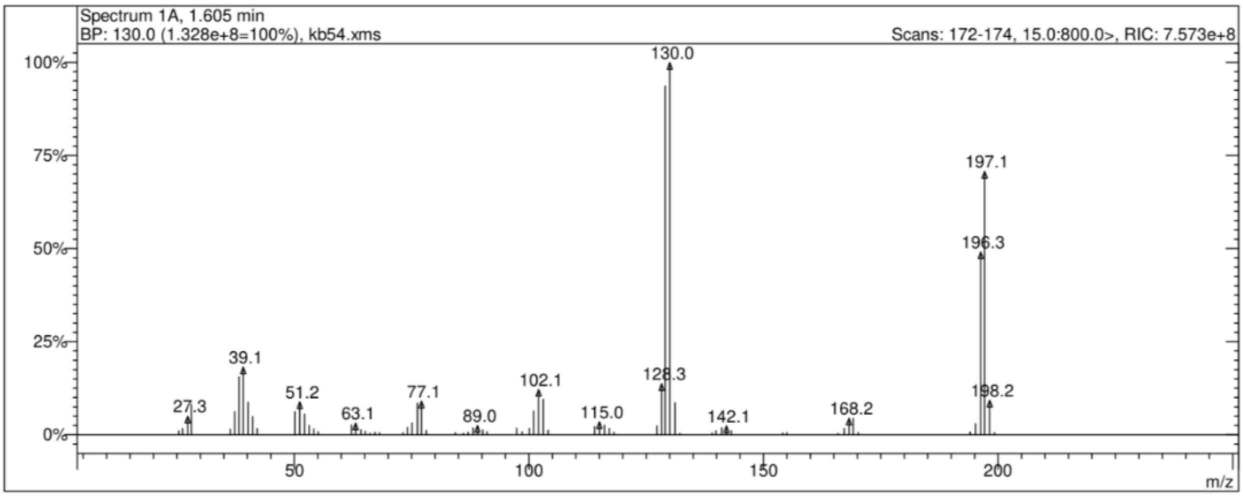

Figure S1c. EI-MS spectrum of compound **2**

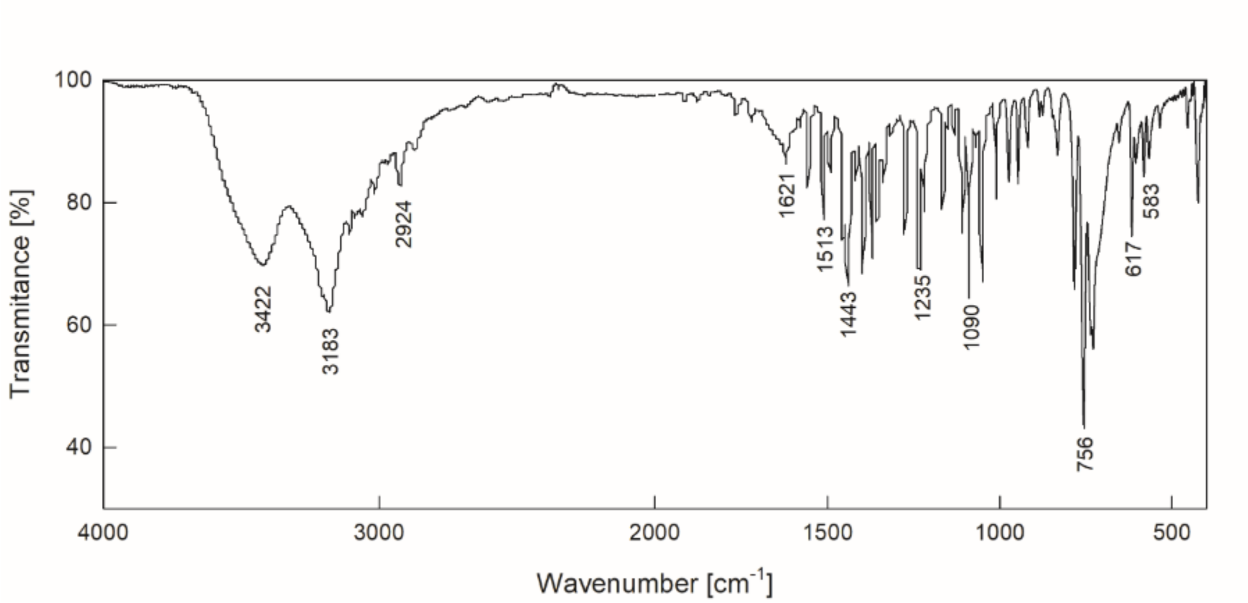

Figure S1d. FT-IR spectrum of compound **2**

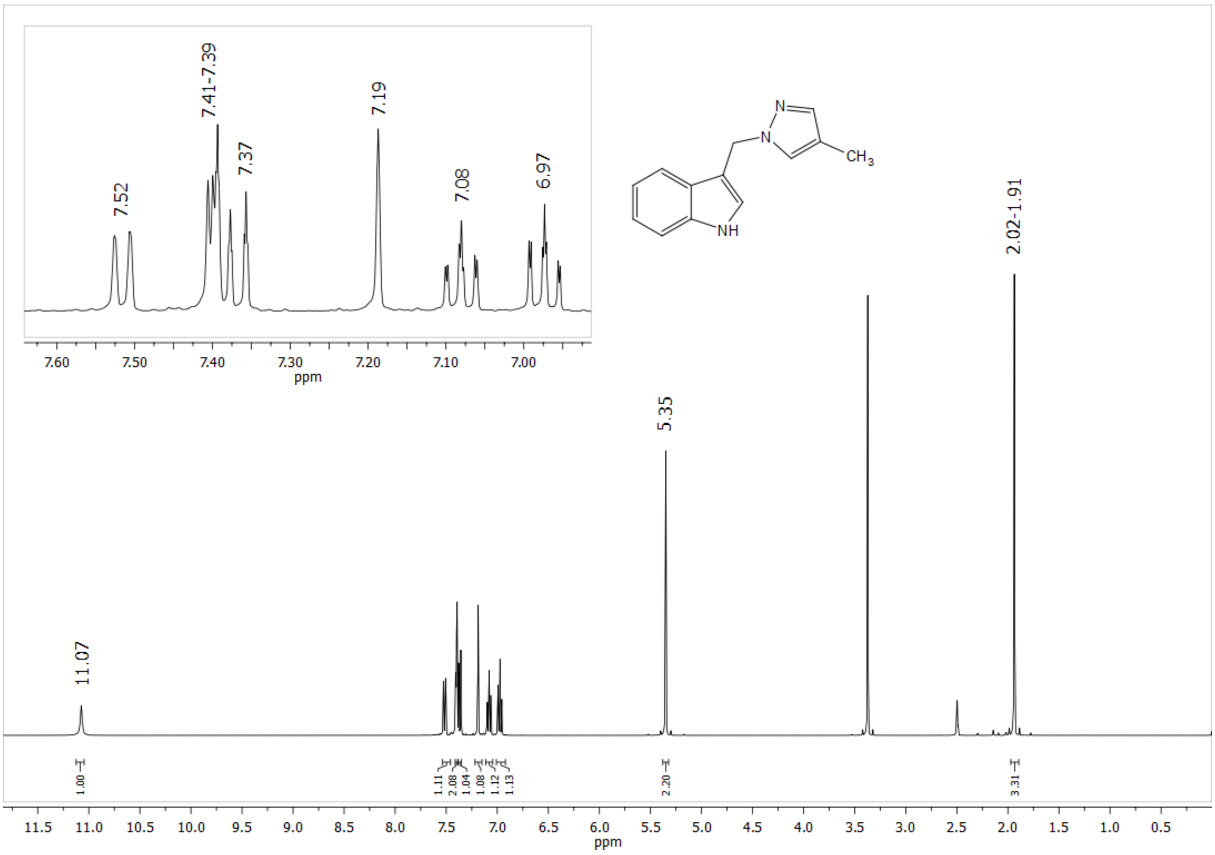

Figure S2a. ^{1}H NMR spectrum of compound **3**

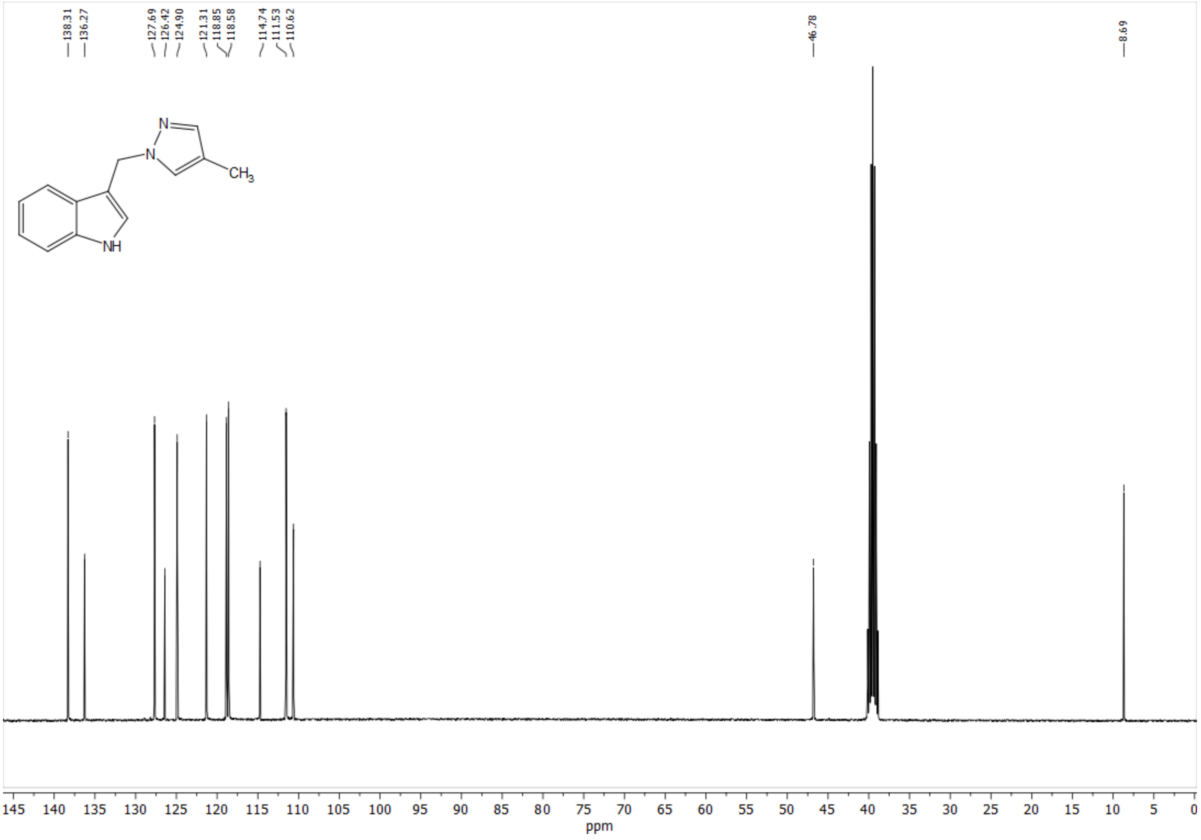

Figure S2b. ^{13}C NMR spectrum of compound **3**

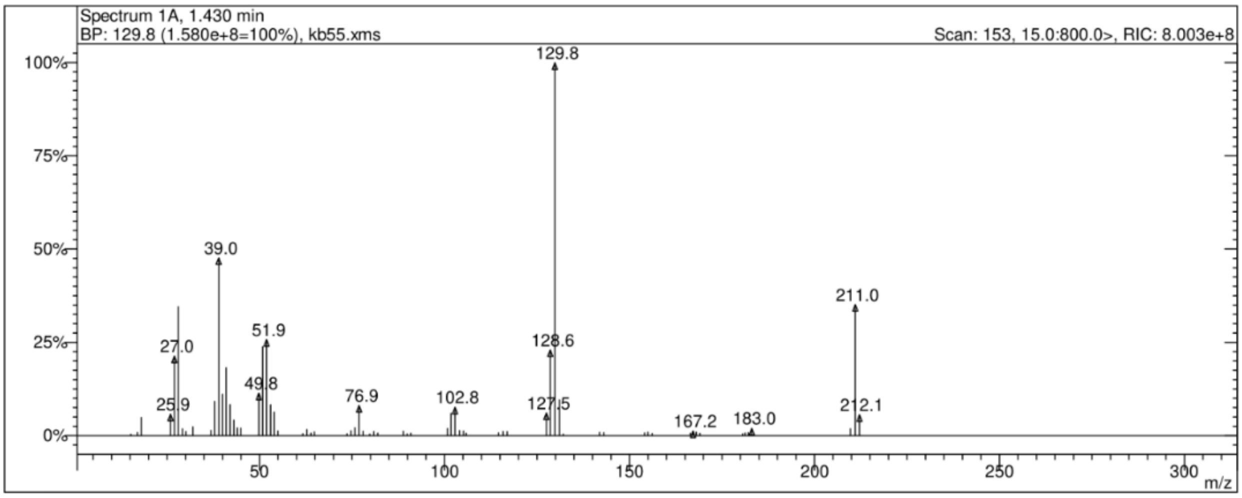

Figure S2c. EI-MS spectrum of compound **3**

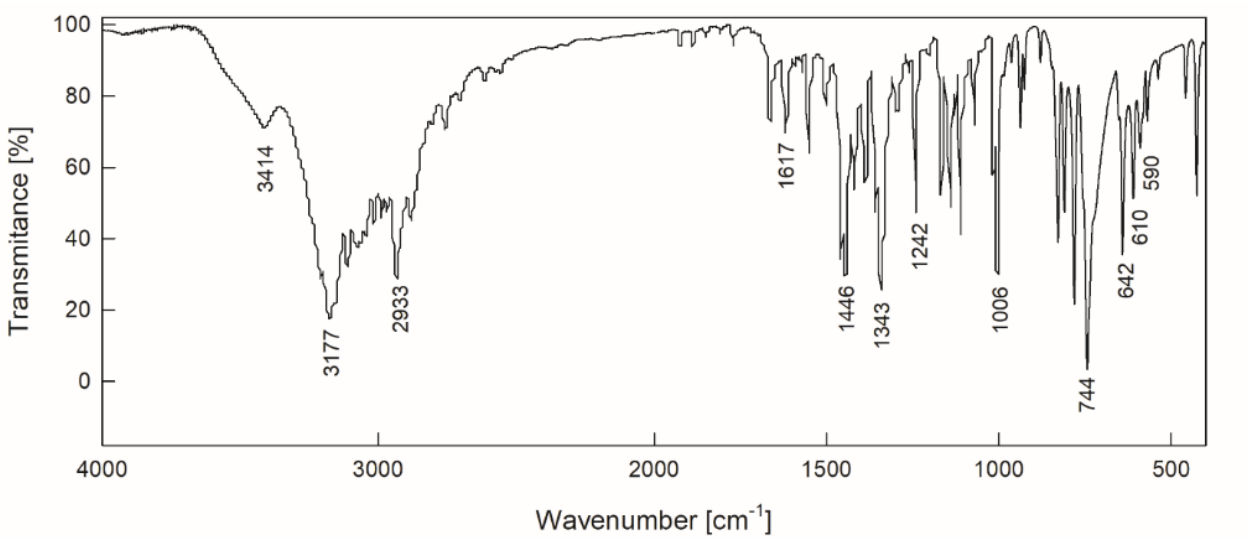

Figure S2d. FT-IR spectrum of compound **3**

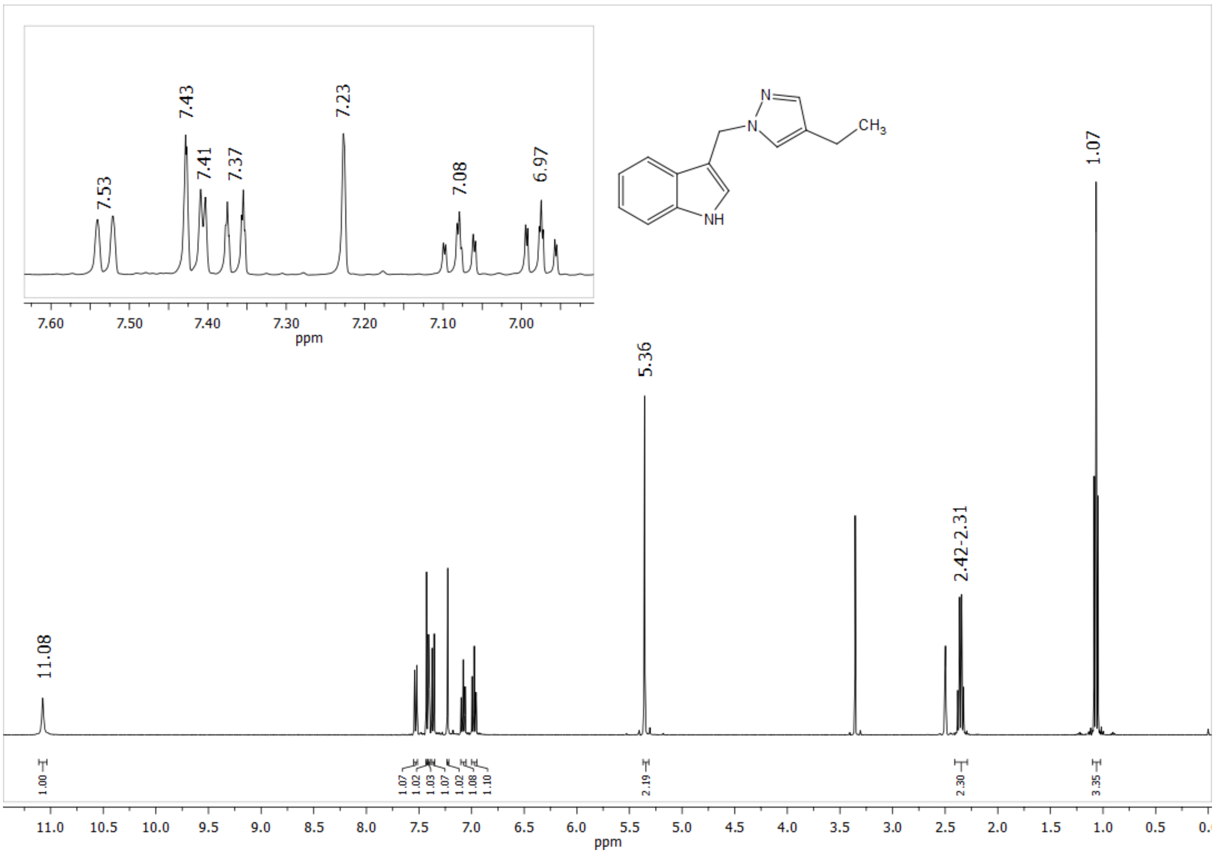

Figure S3a. ^{1}H NMR spectrum of compound **4**

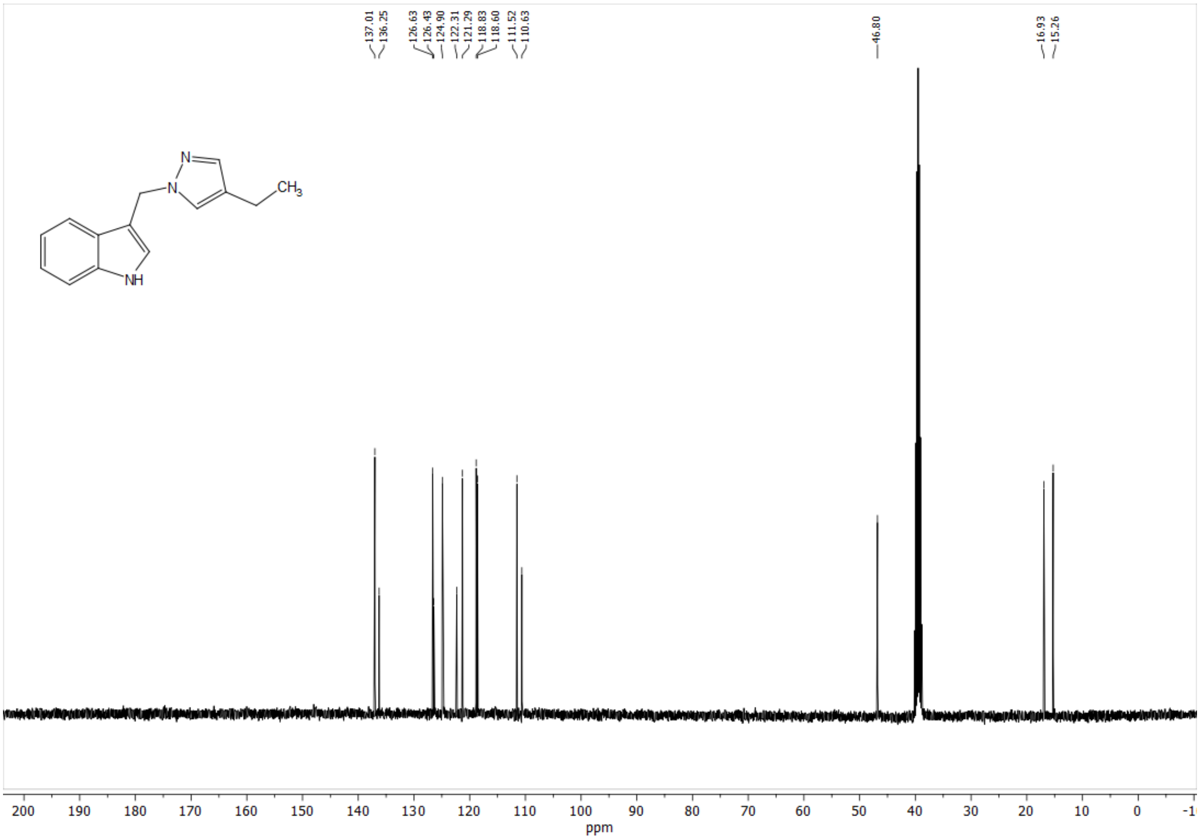

Figure S3b. ^{13}C NMR spectrum of compound **4**

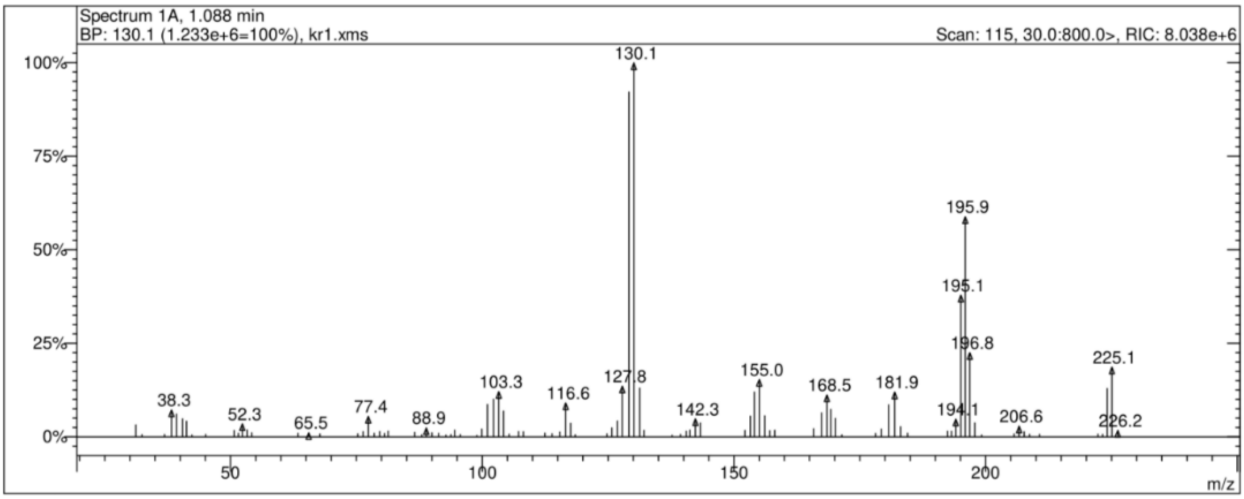

Figure S3c. EI-MS spectrum of compound **4**

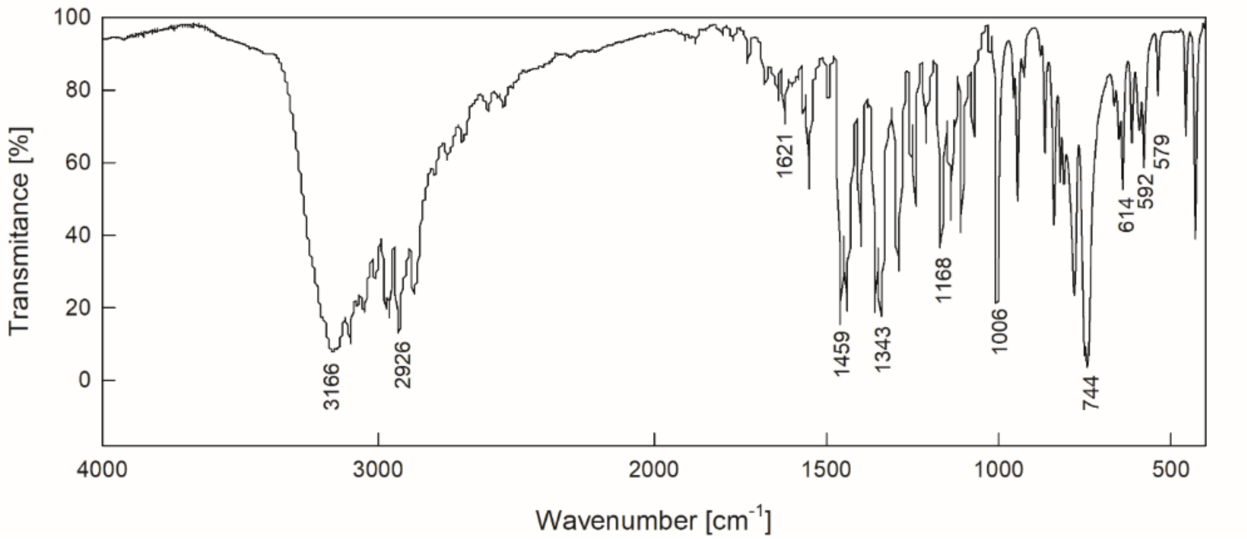

Figure S3d. FT-IR spectrum of compound **4**

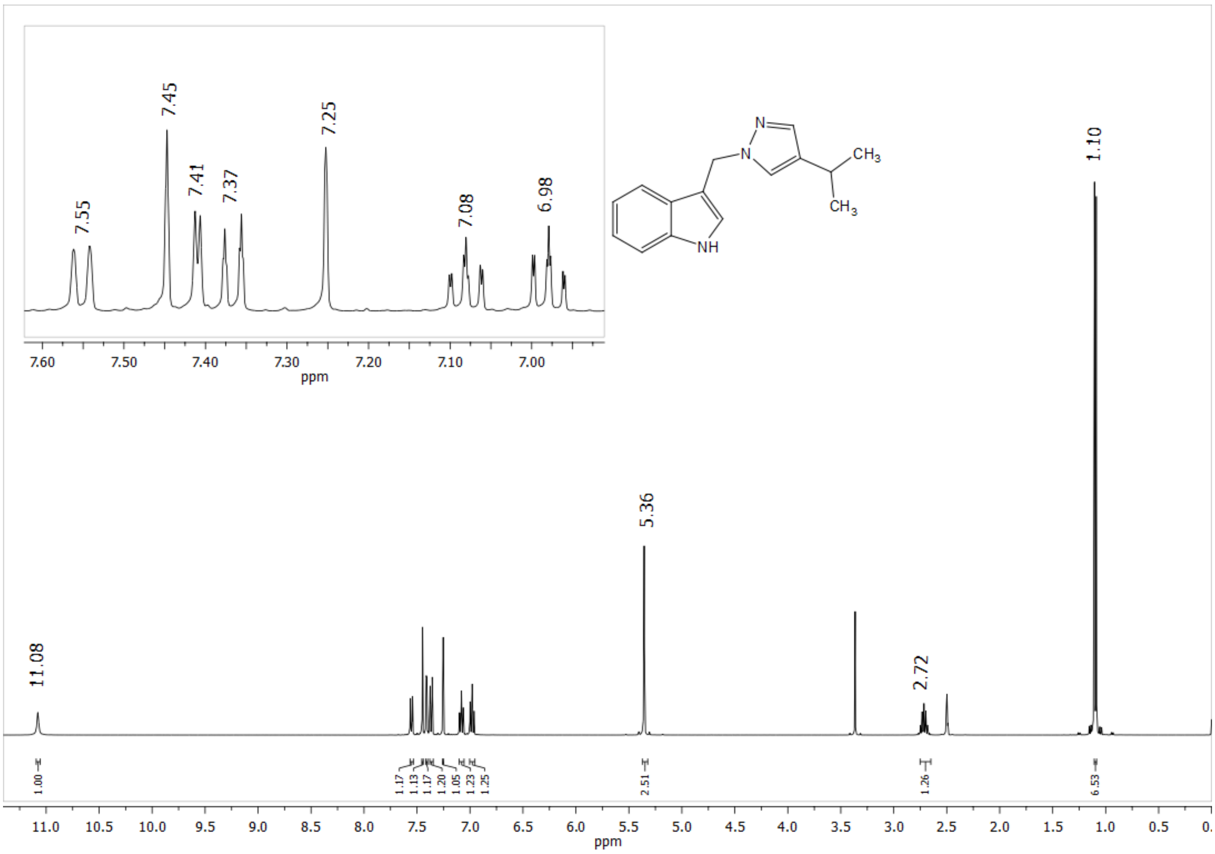

Figure S4a. ^{1}H NMR spectrum of compound **5**

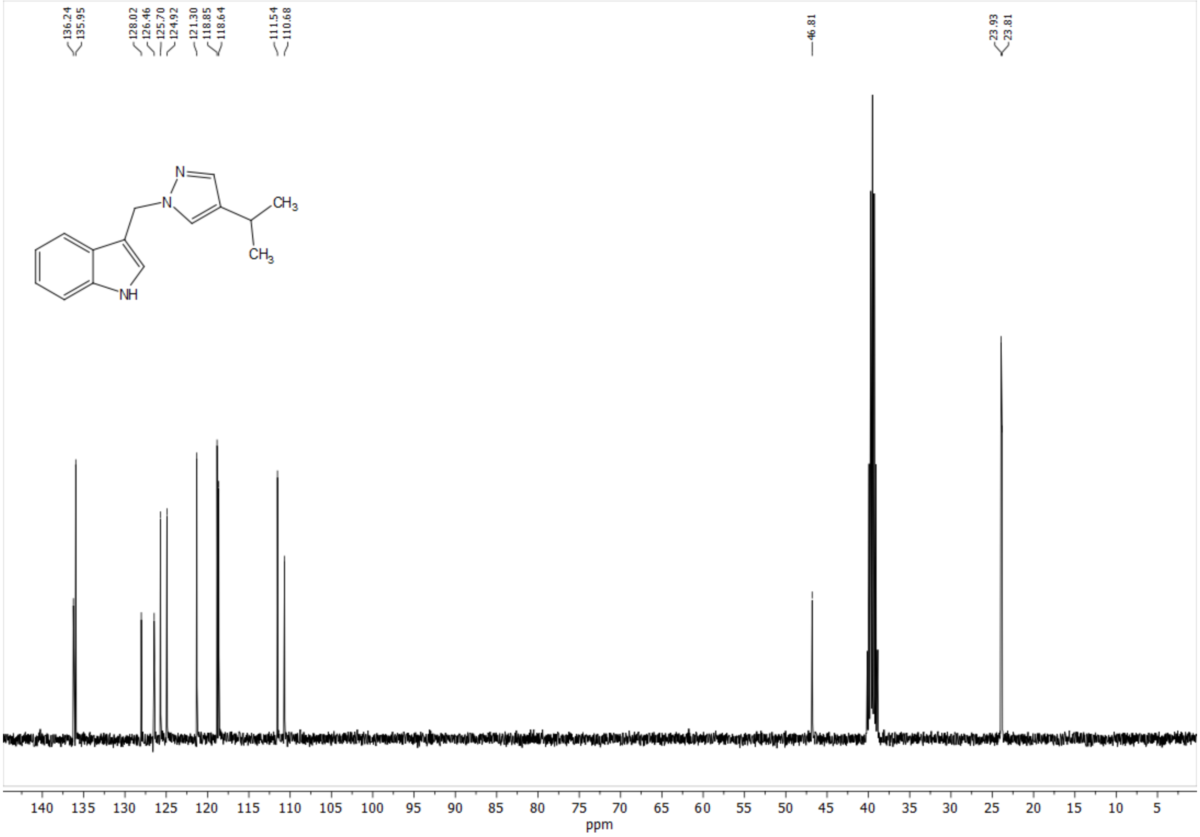

Figure S4b. ^{13}C NMR spectrum of compound **5**

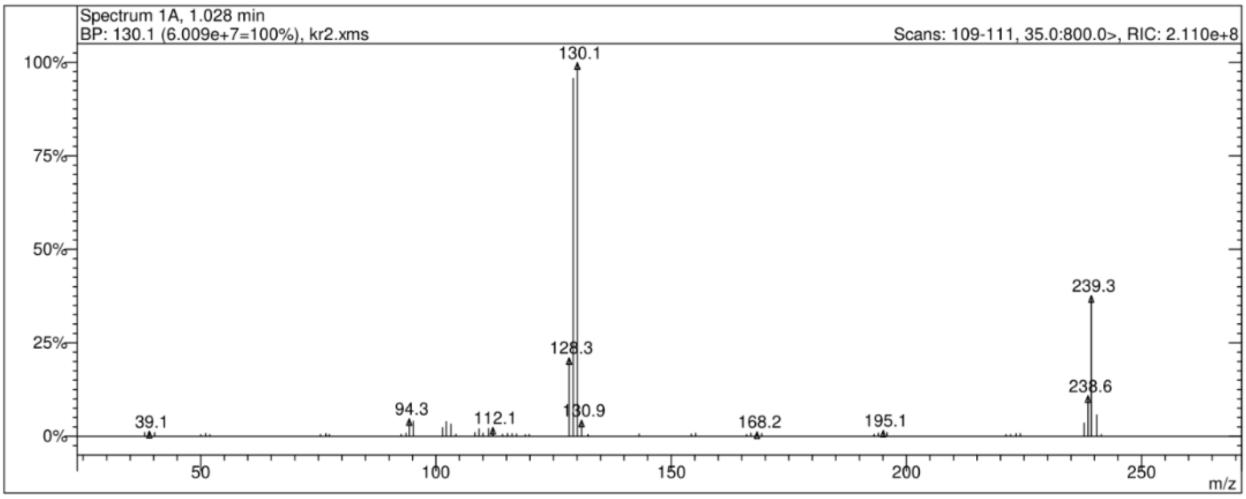

Figure S4c. EI-MS spectrum of compound **5**

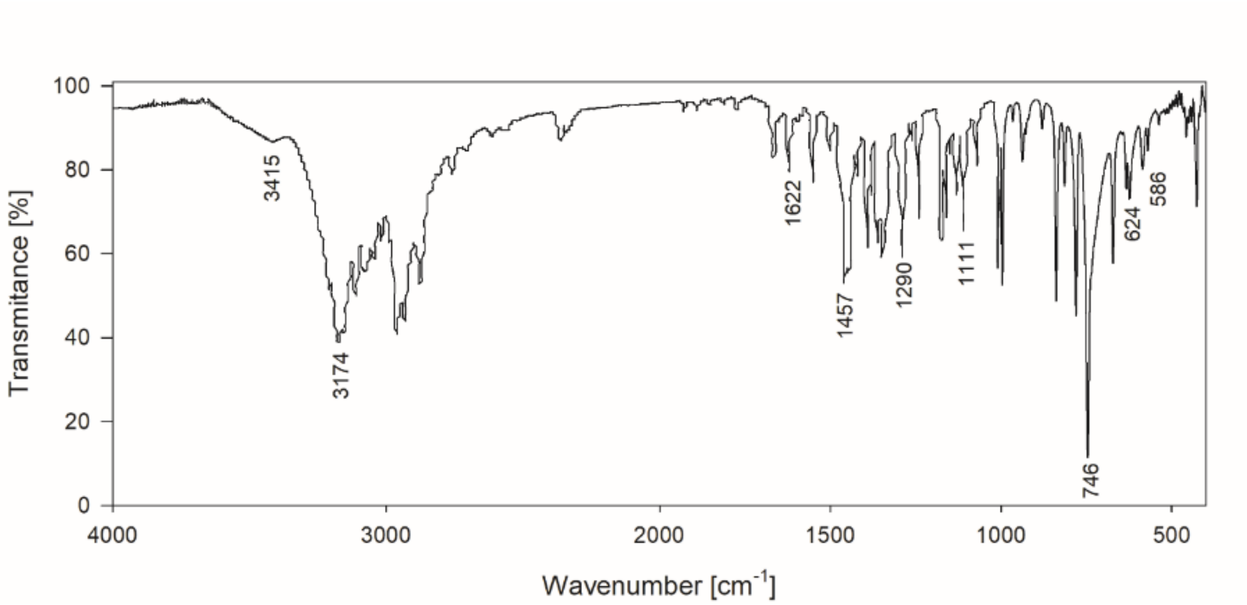

Figure S4d. FT-IR spectrum of compound **5**

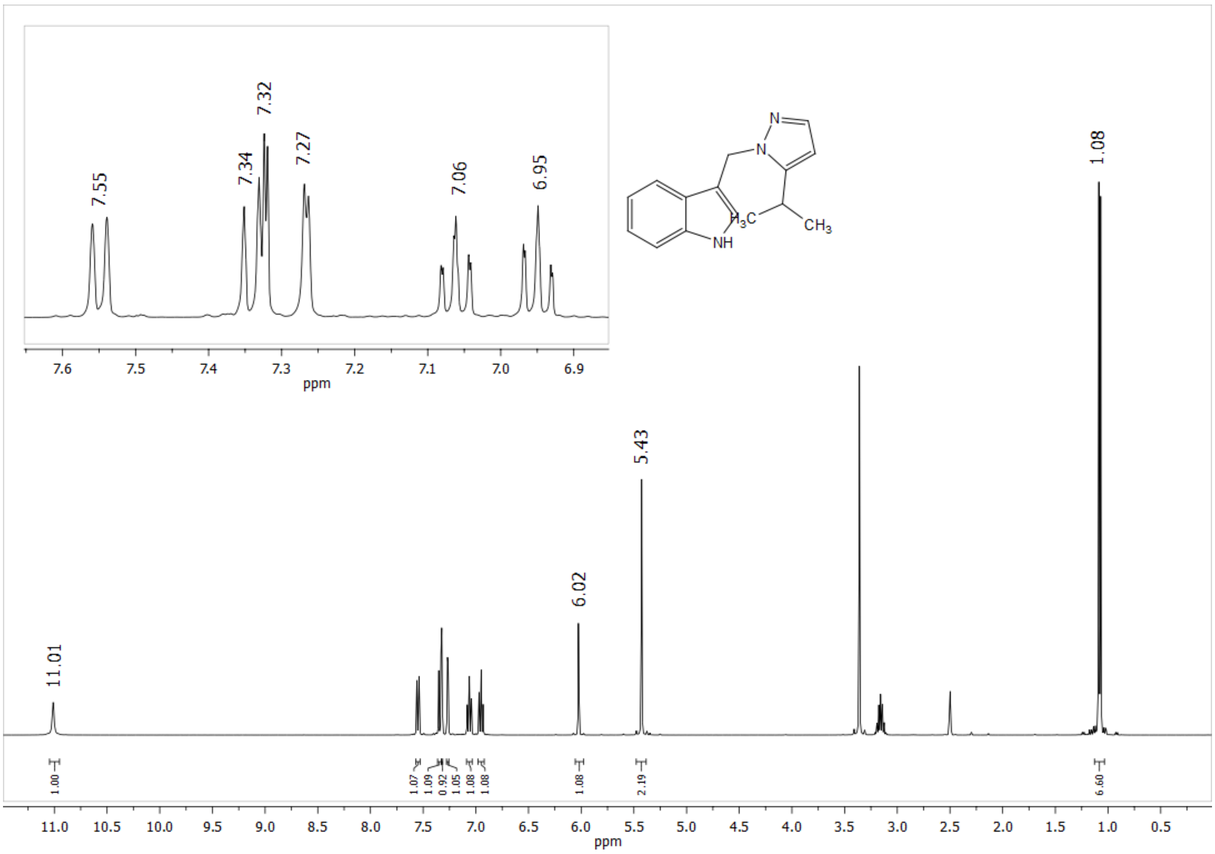

Figure S5a. ^{1}H NMR spectrum of compound **6**

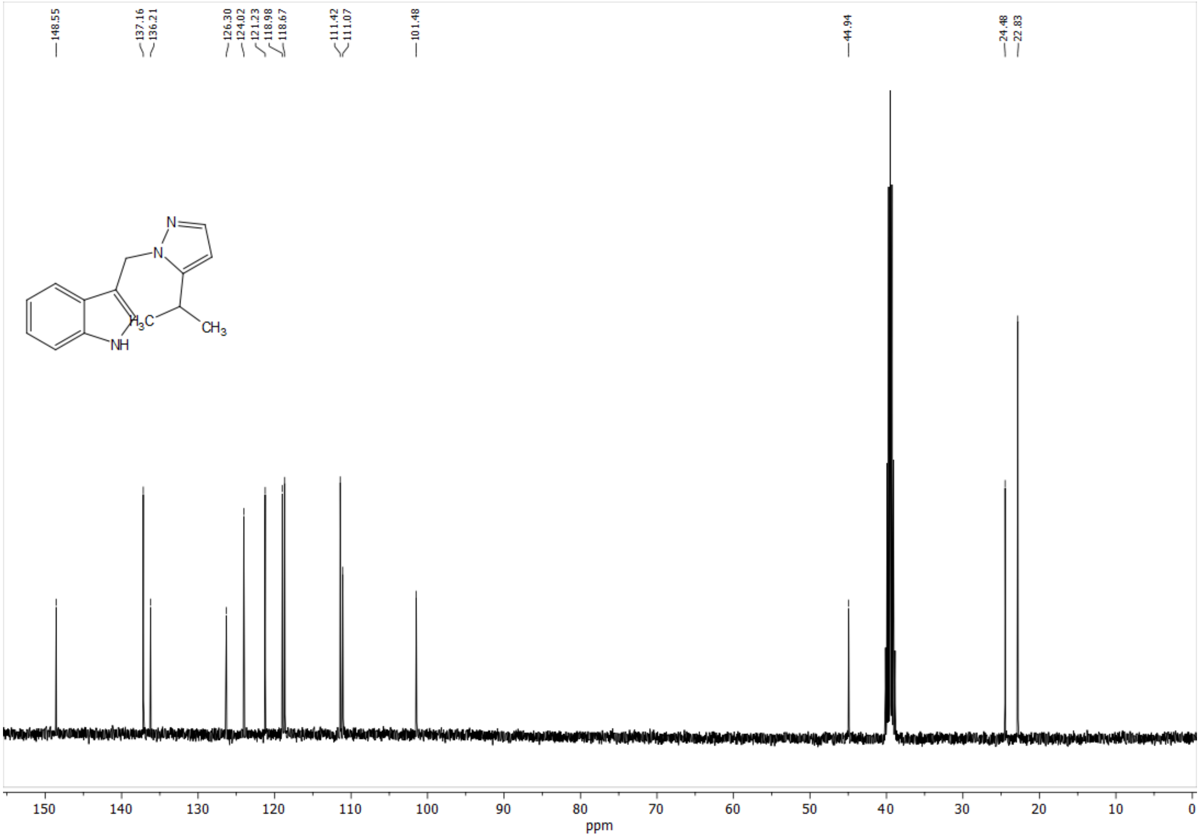

Figure S5b. ^{13}C NMR spectrum of compound **6**

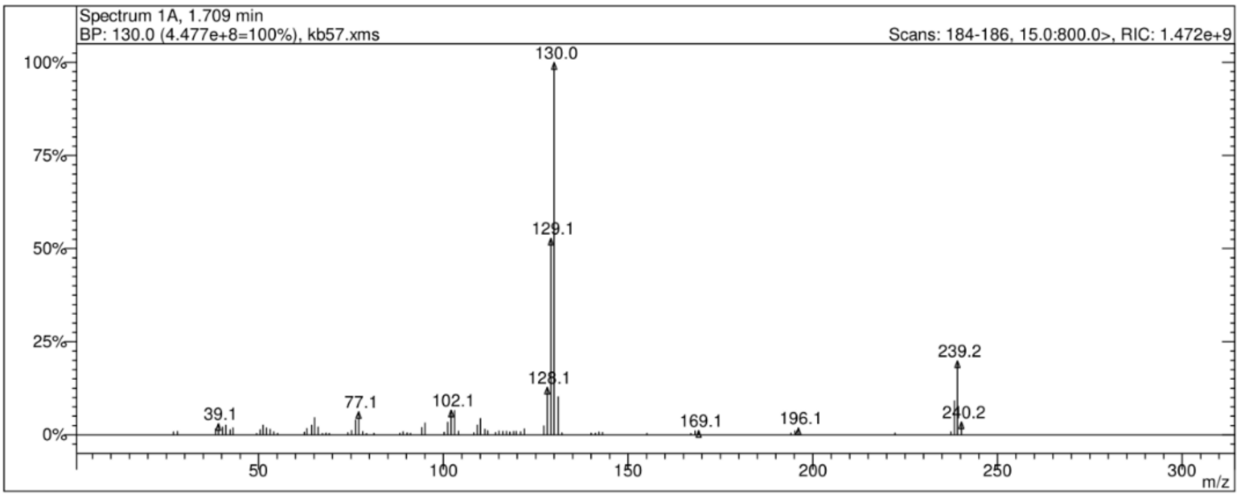

Figure S5c. EI-MS spectrum of compound **6**

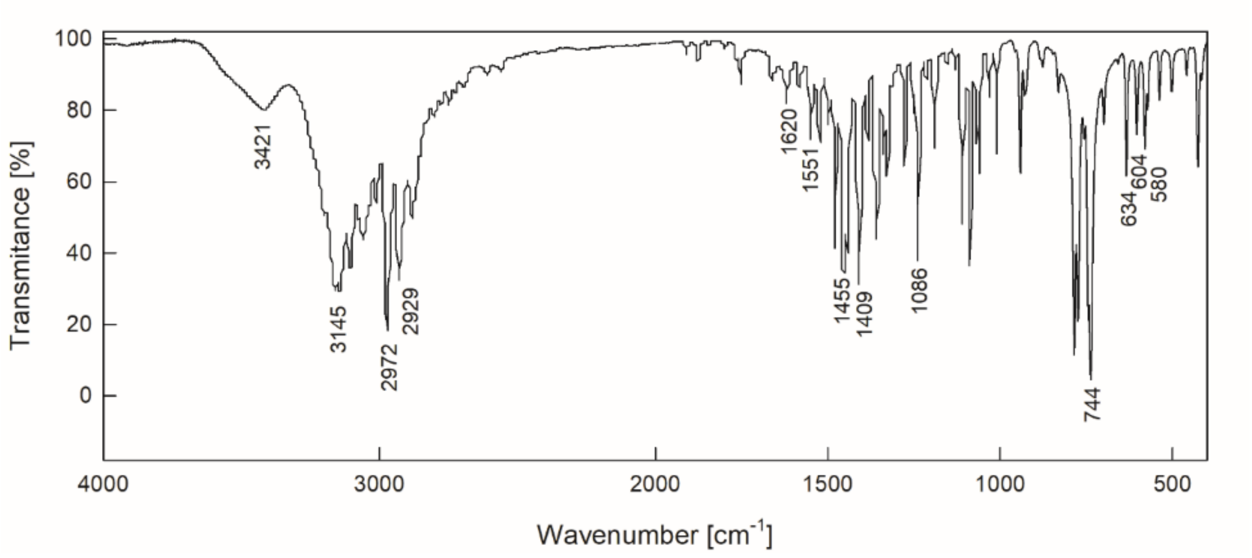

Figure S5d. FT-IR spectrum of compound **6**

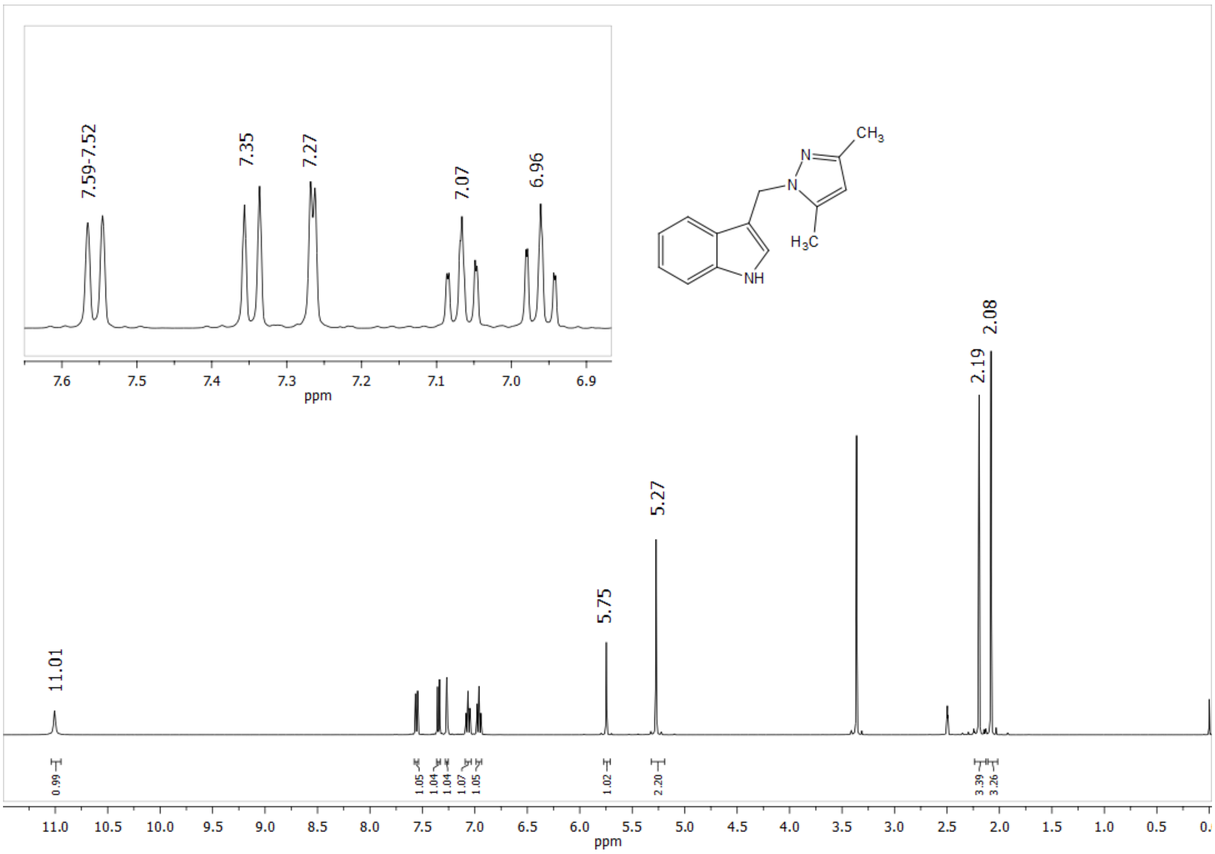

Figure S6a. ^{1}H NMR spectrum of compound **7**

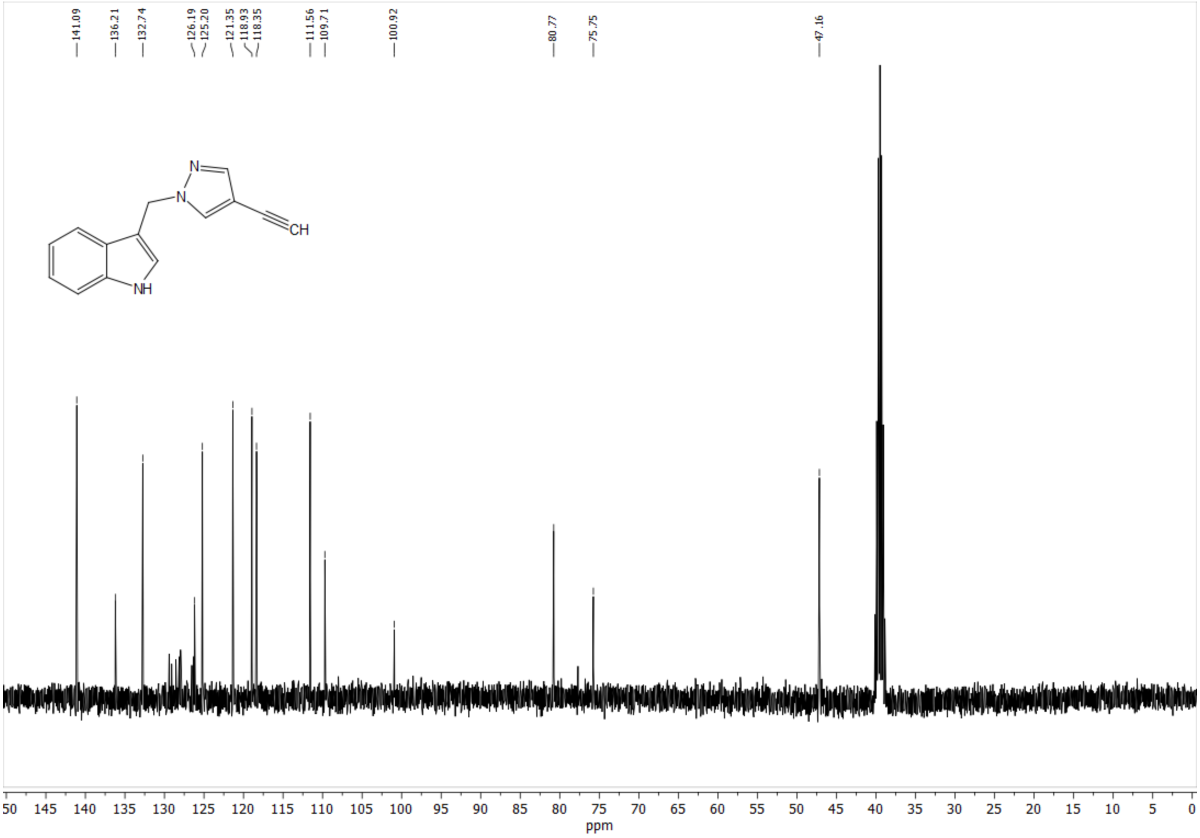

Figure S6b. ^{13}C NMR spectrum of compound **7**

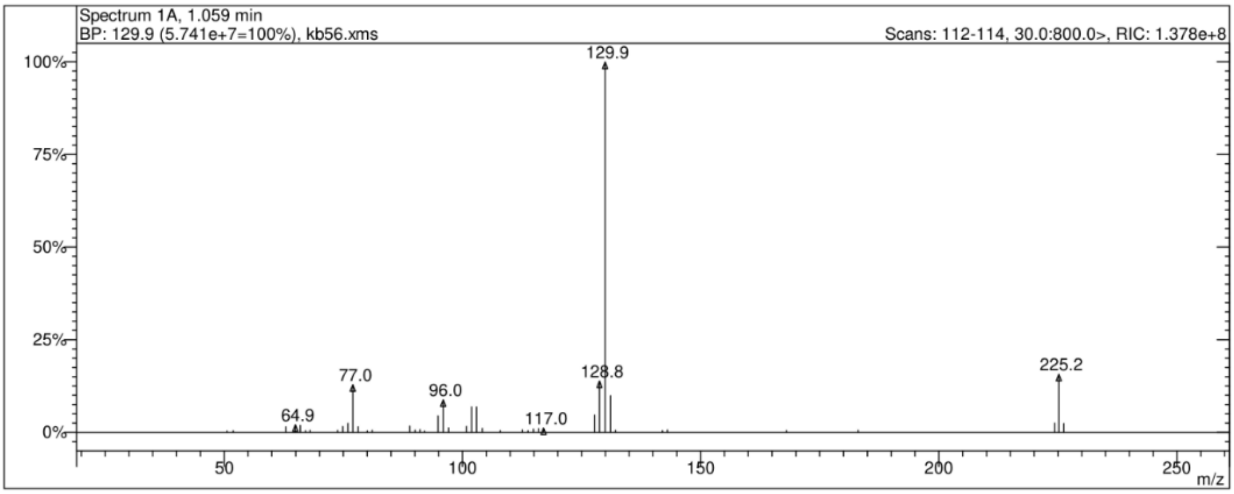

Figure S6c. EI-MS spectrum of compound **7**

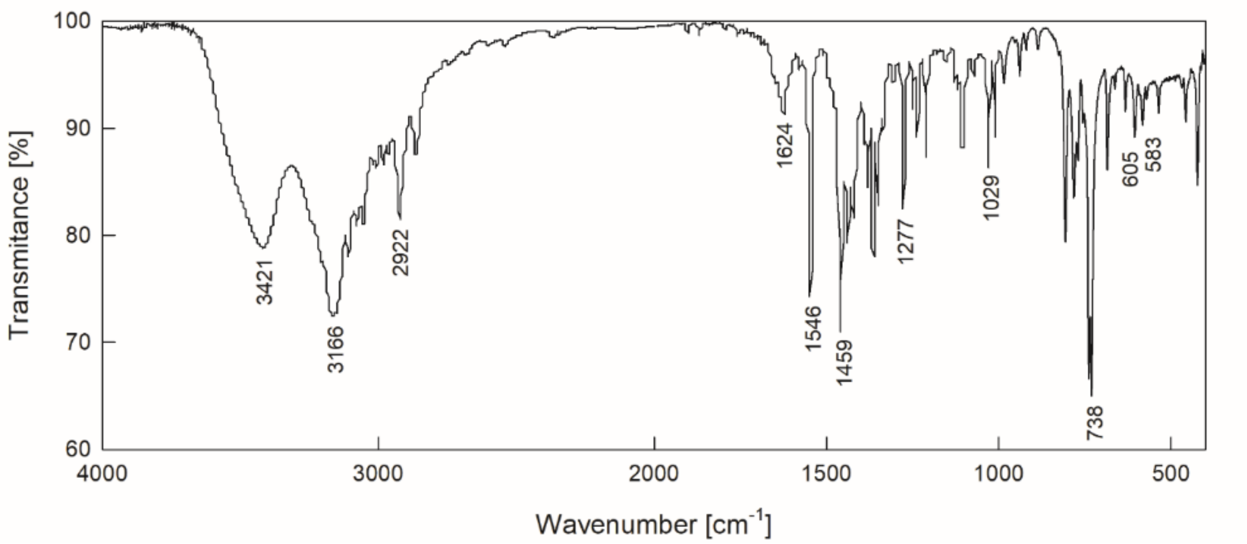

Figure S6d. FT-IR spectrum of compound **7**

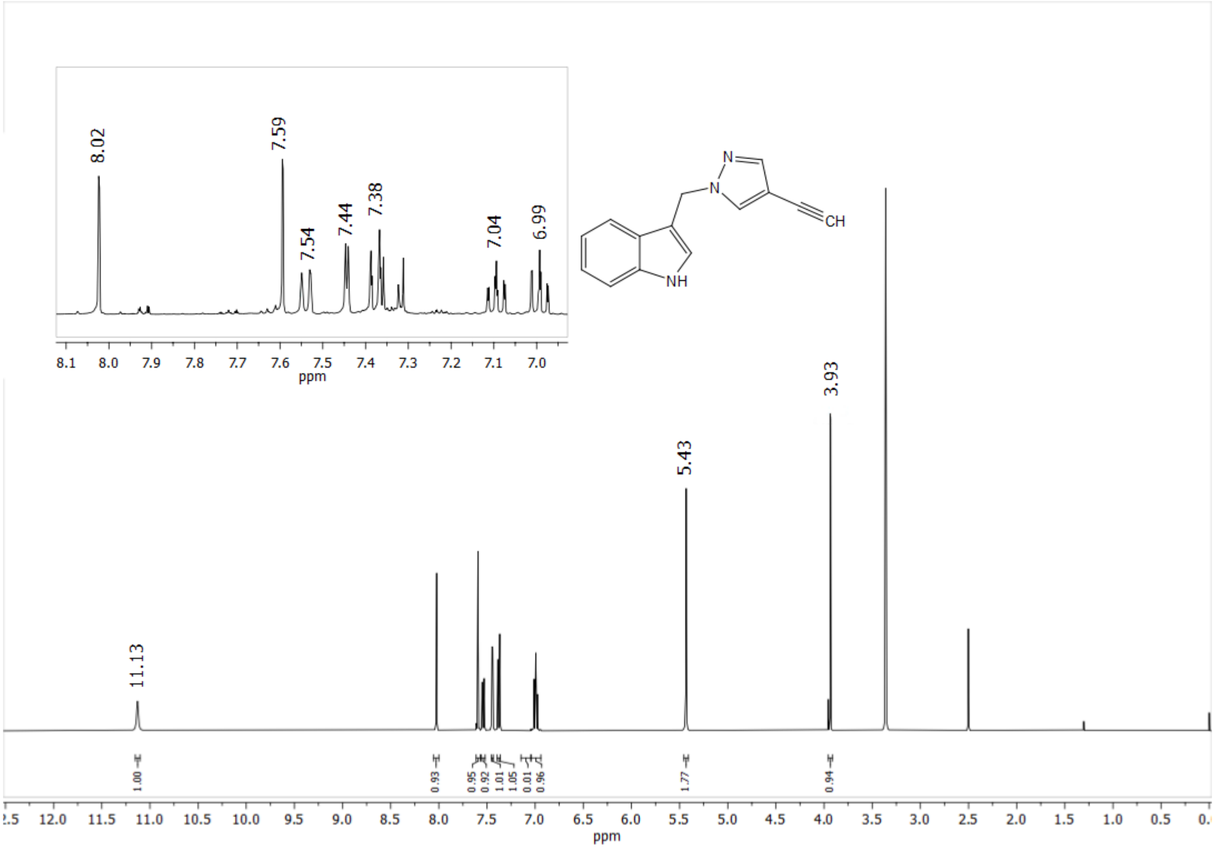

Figure S7a. ^{1}H NMR spectrum of compound **8**

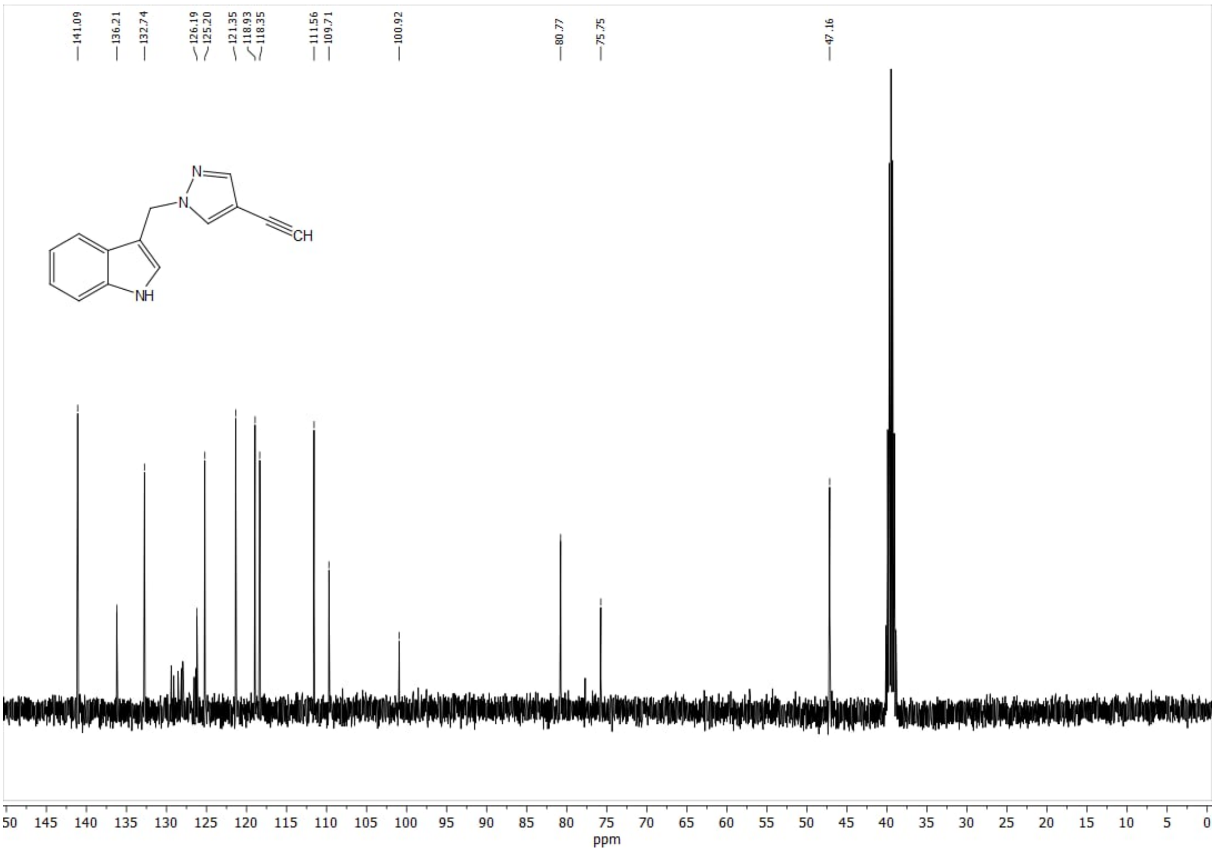

Figure S7b. ^{13}C NMR spectrum of compound **8**

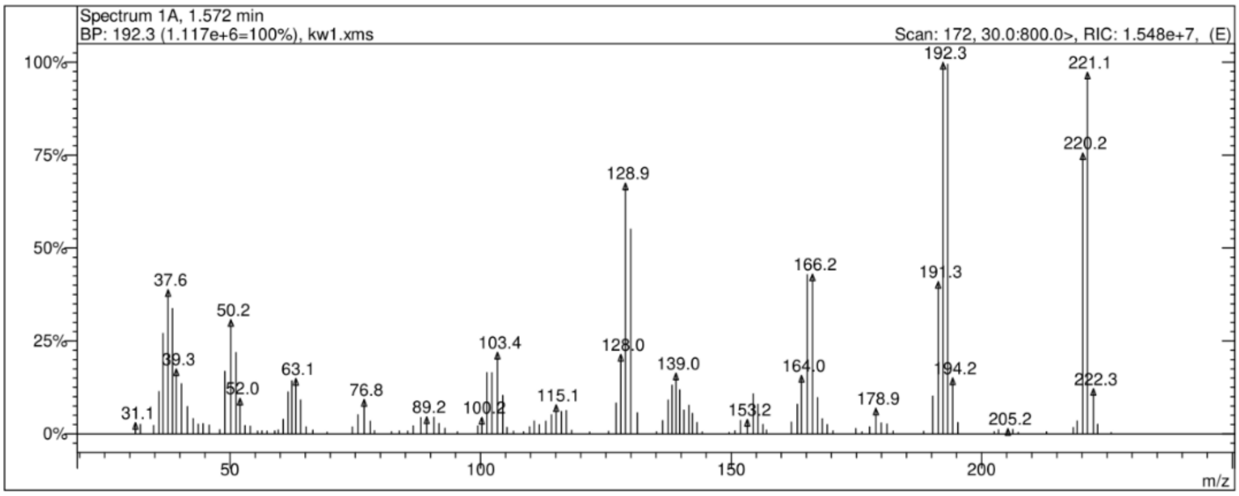

Figure S7c. EI-MS spectrum of compound **8**

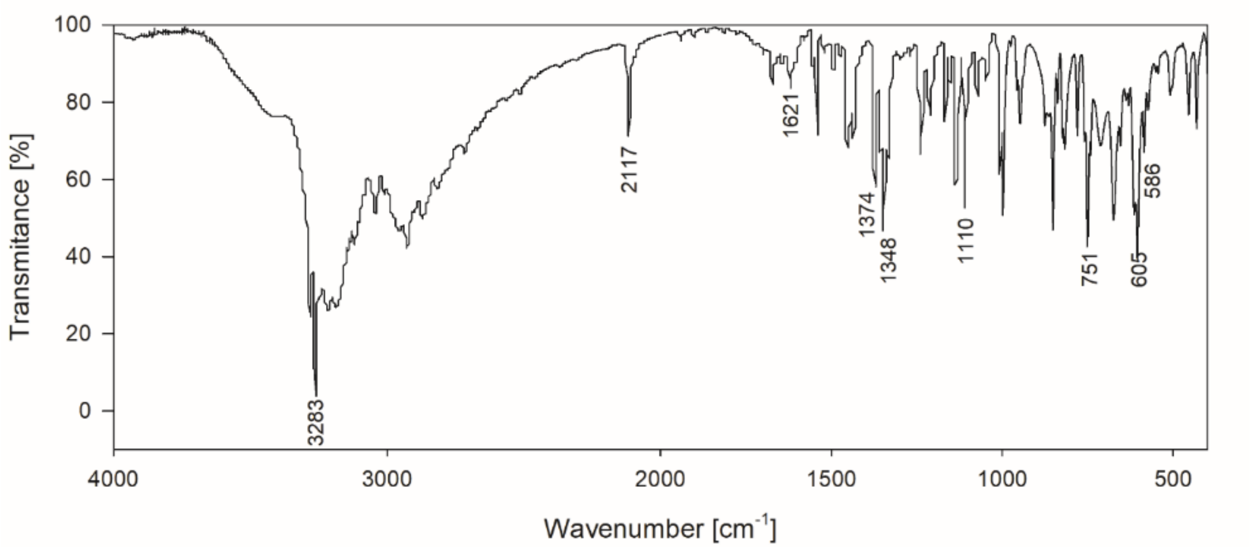

Figure S7d. FT-IR spectrum of compound **8**

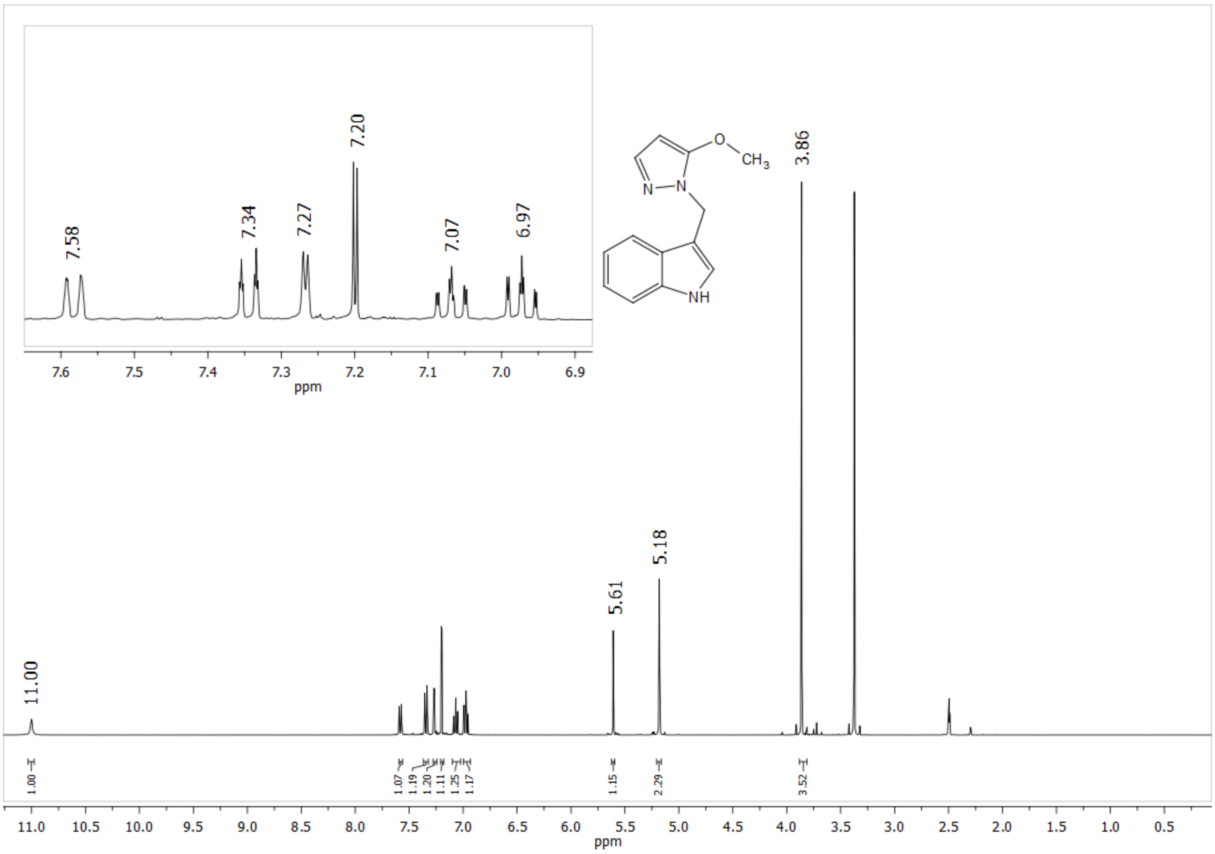

Figure S8a. ^{1}H NMR spectrum of compound **9**

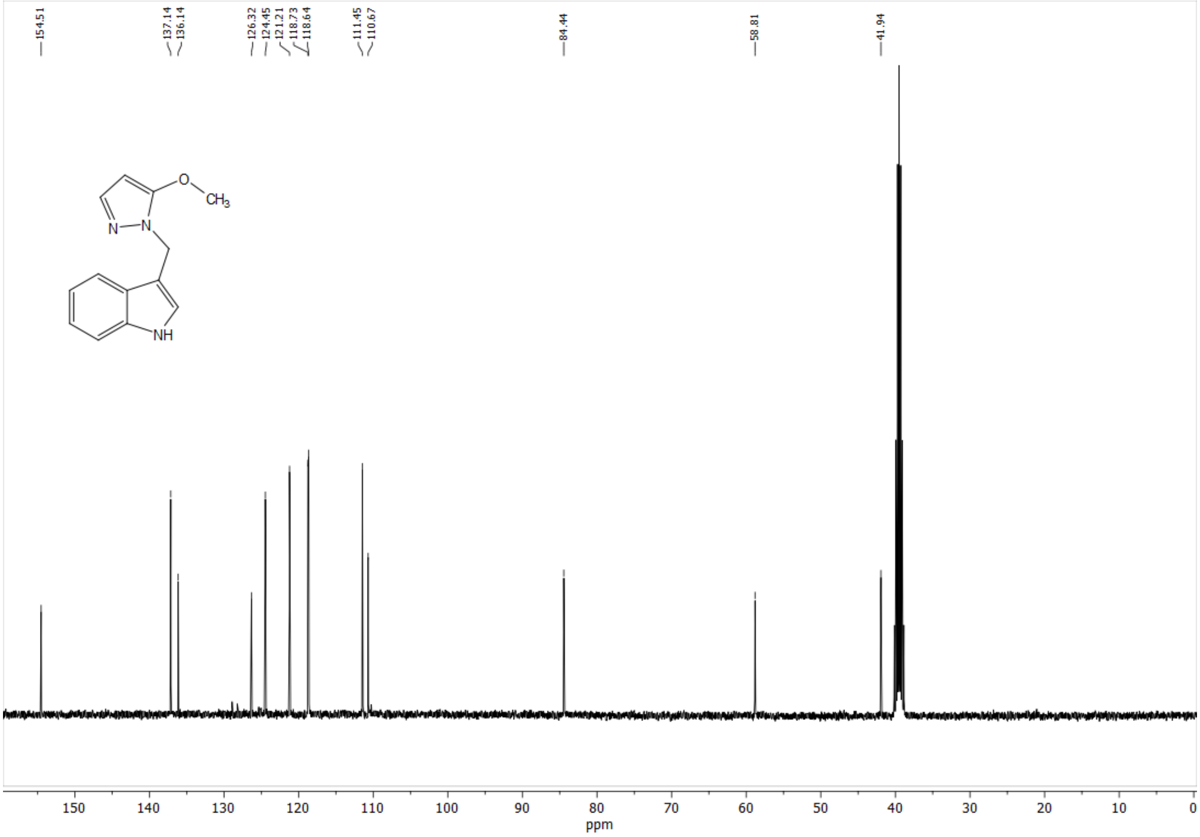

Figure S8b. ^{13}C NMR spectrum of compound **9**

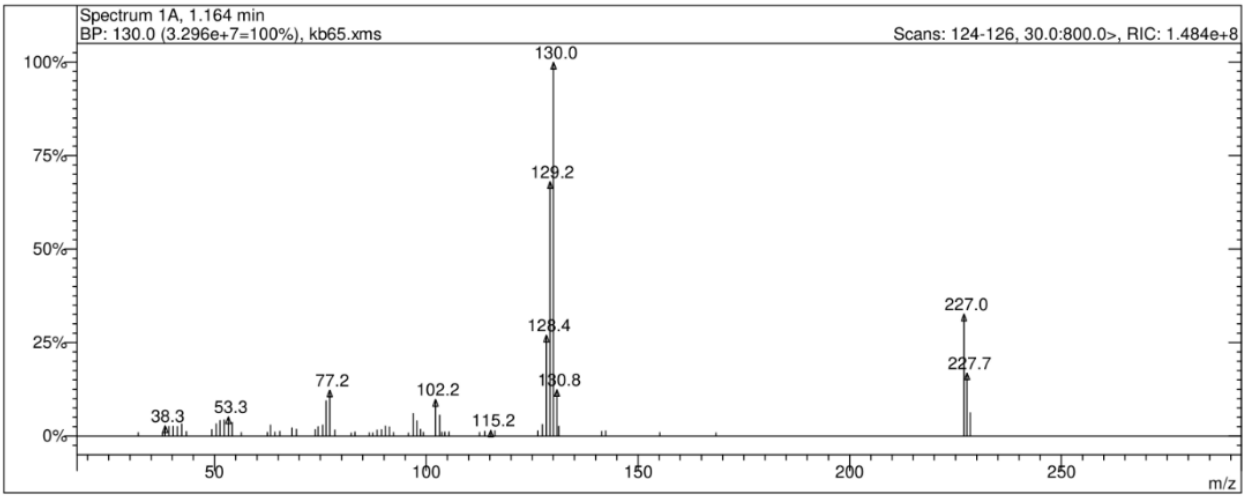

Figure S8c. EI-MS spectrum of compound **9**

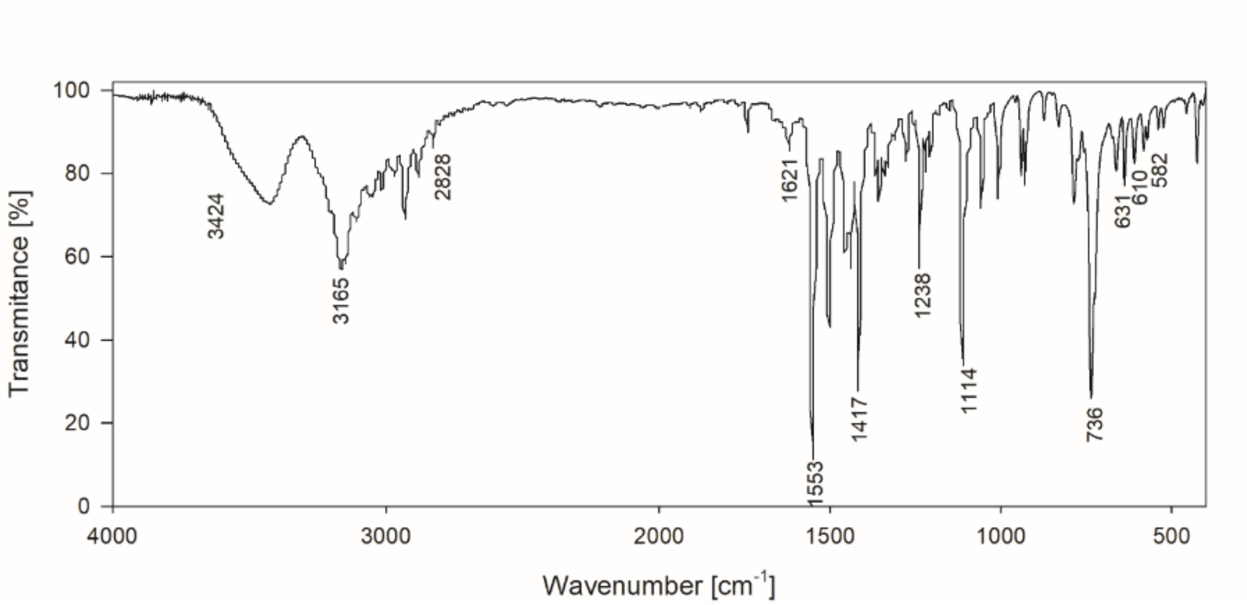

Figure S8d. FT-IR spectrum of compound **9**

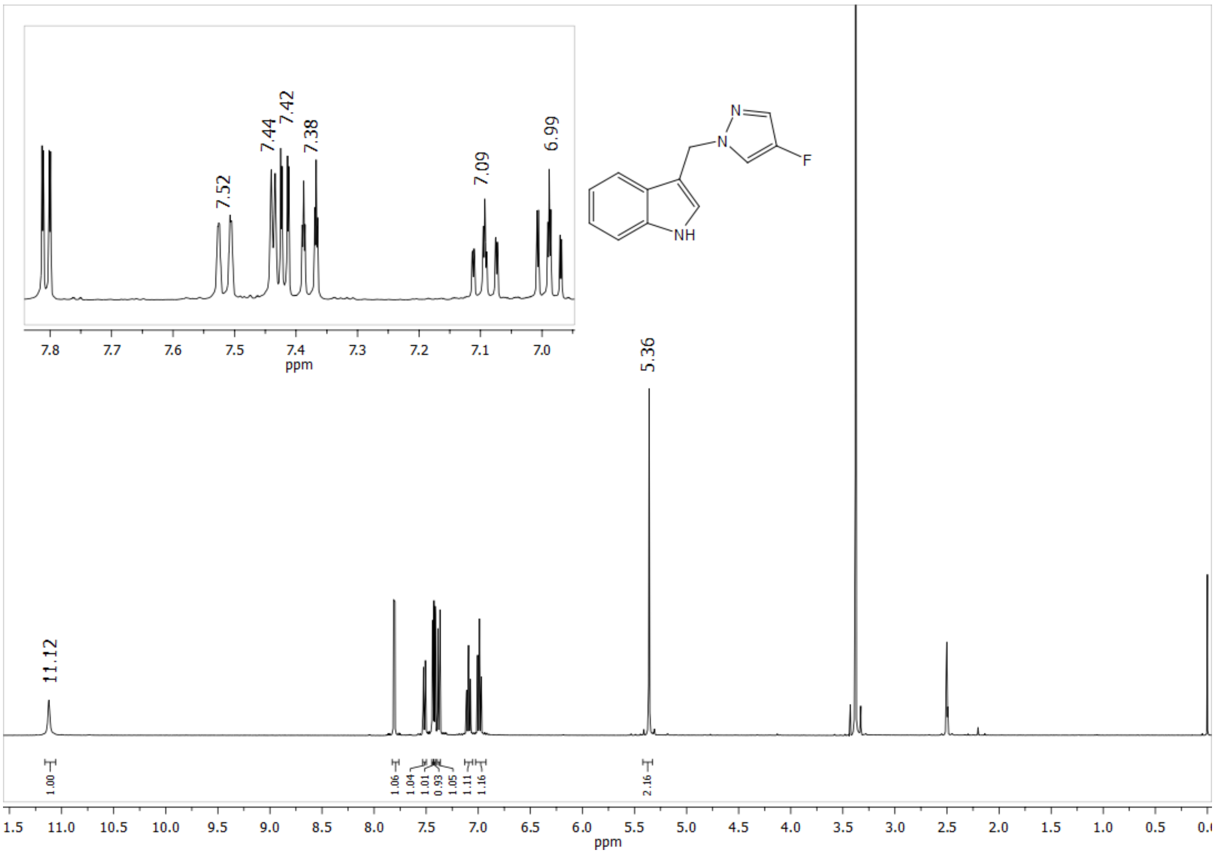

Figure S9a. ^{1}H NMR spectrum of compound **10**

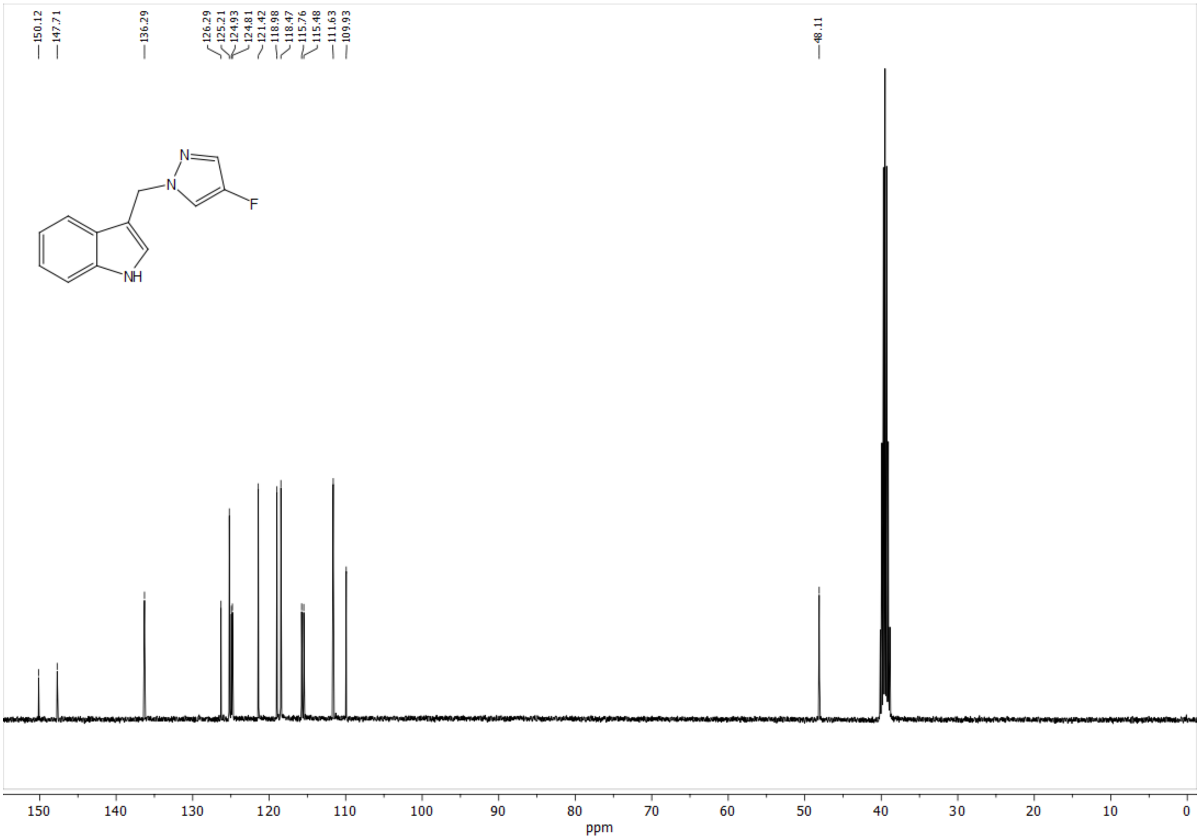

Figure S9b. ^{13}C NMR spectrum of compound **10**

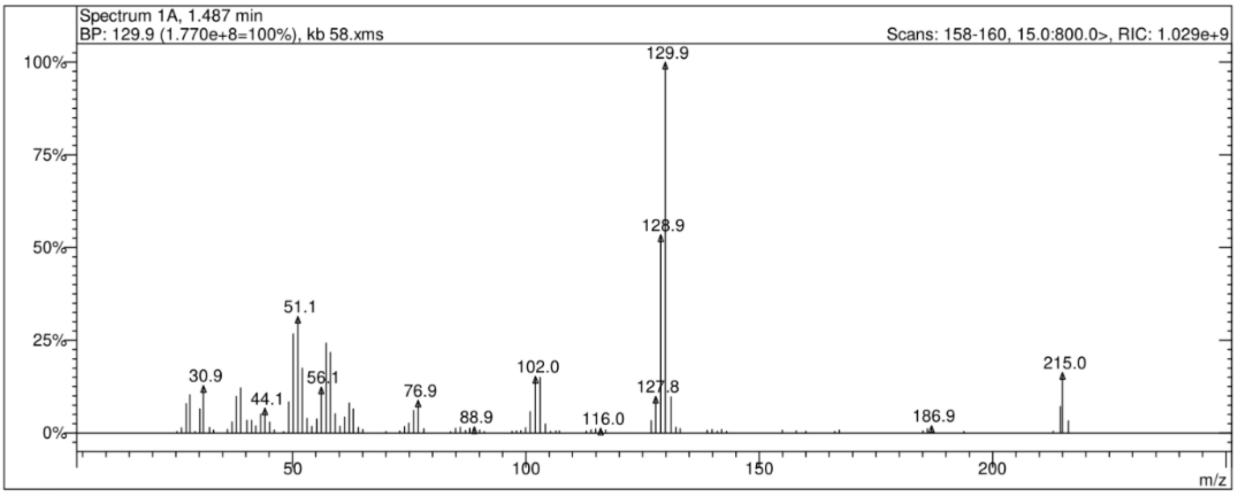

Figure S9c. EI-MS spectrum of compound **10**

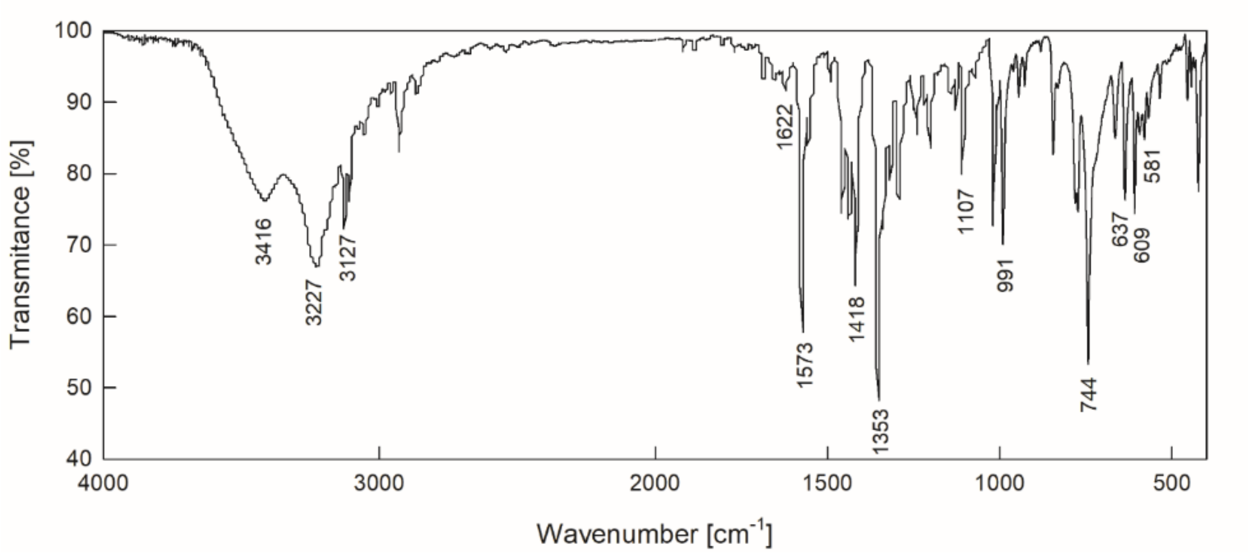

Figure S9d. FT-IR spectrum of compound **10**

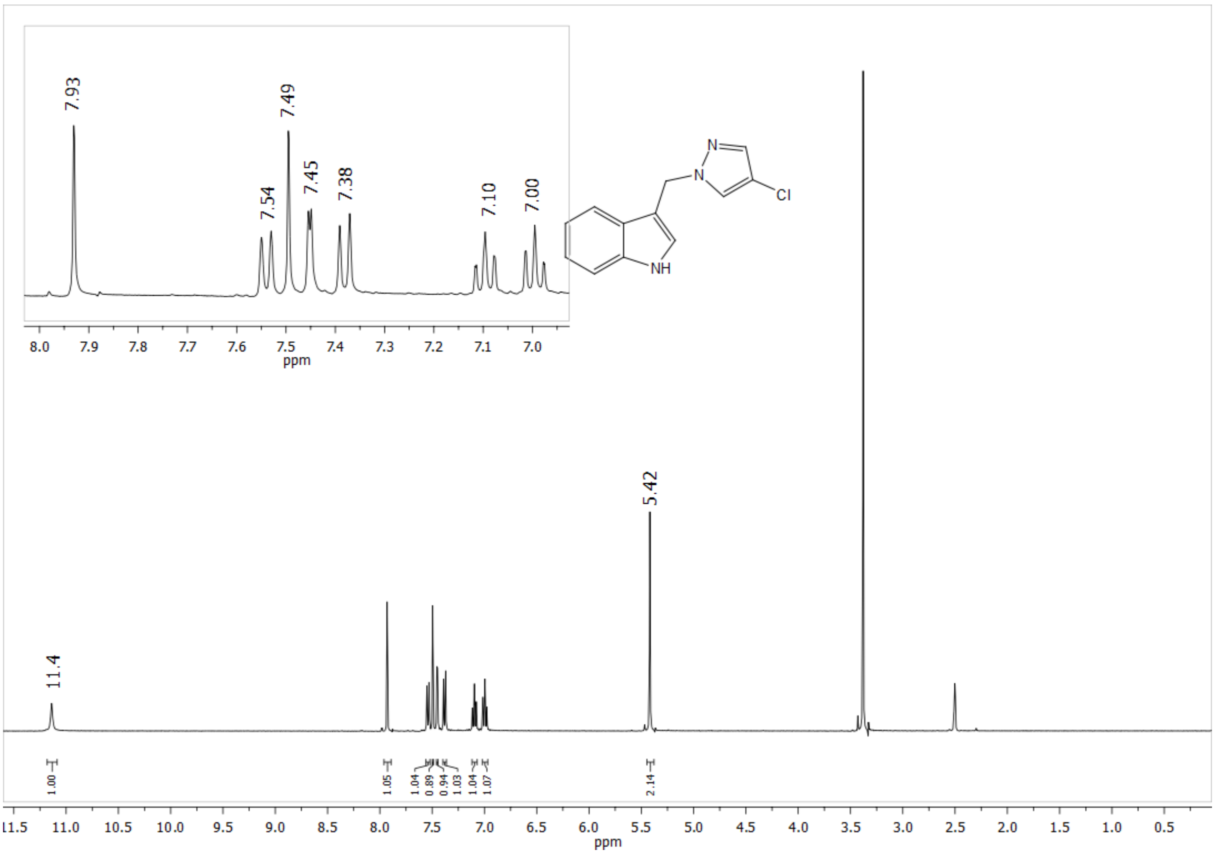

Figure S10a. ^{1}H NMR spectrum of compound **11**

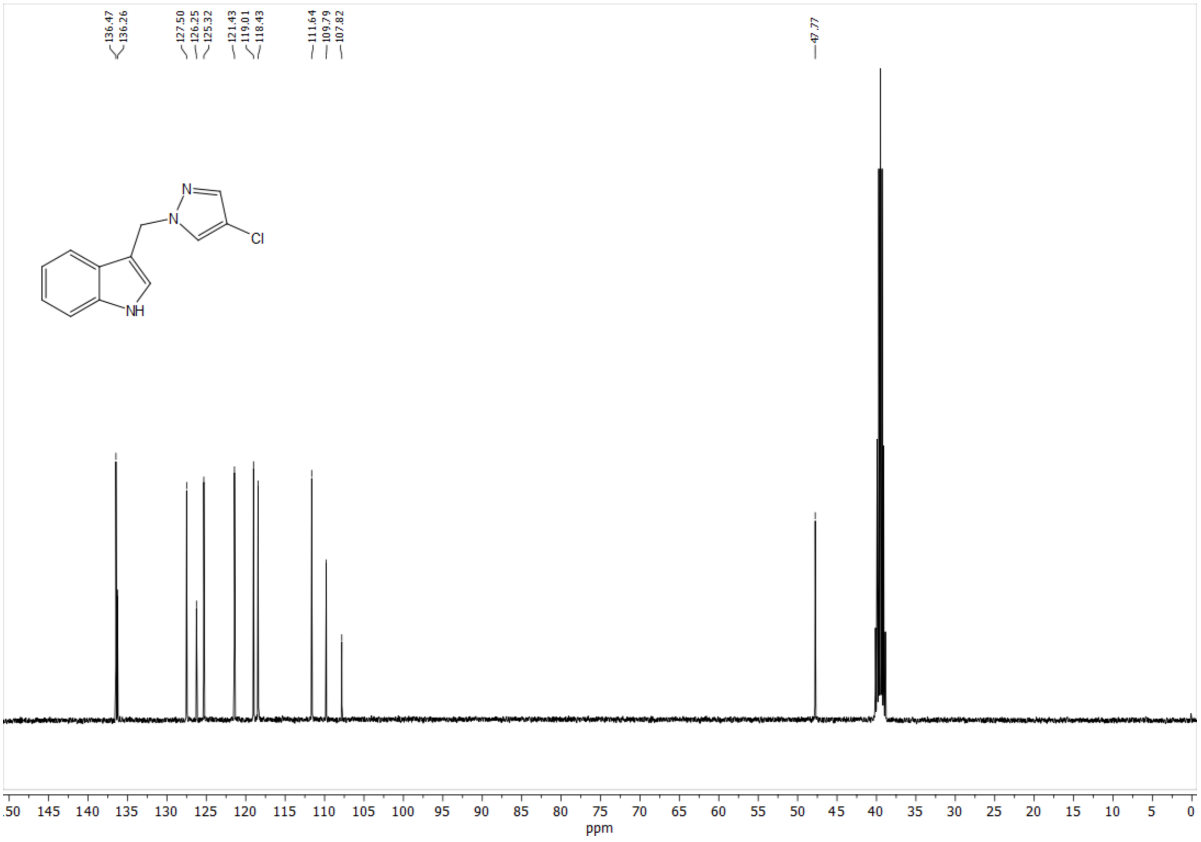

Figure S10b. ^{13}C NMR spectrum of compound **11**

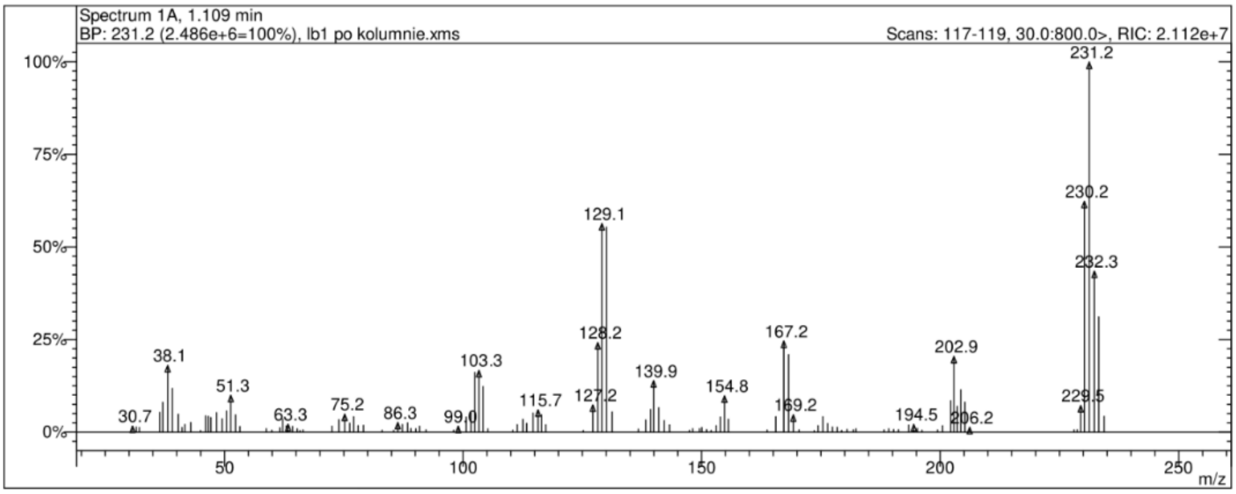

Figure S10c. EI-MS spectrum of compound **11**

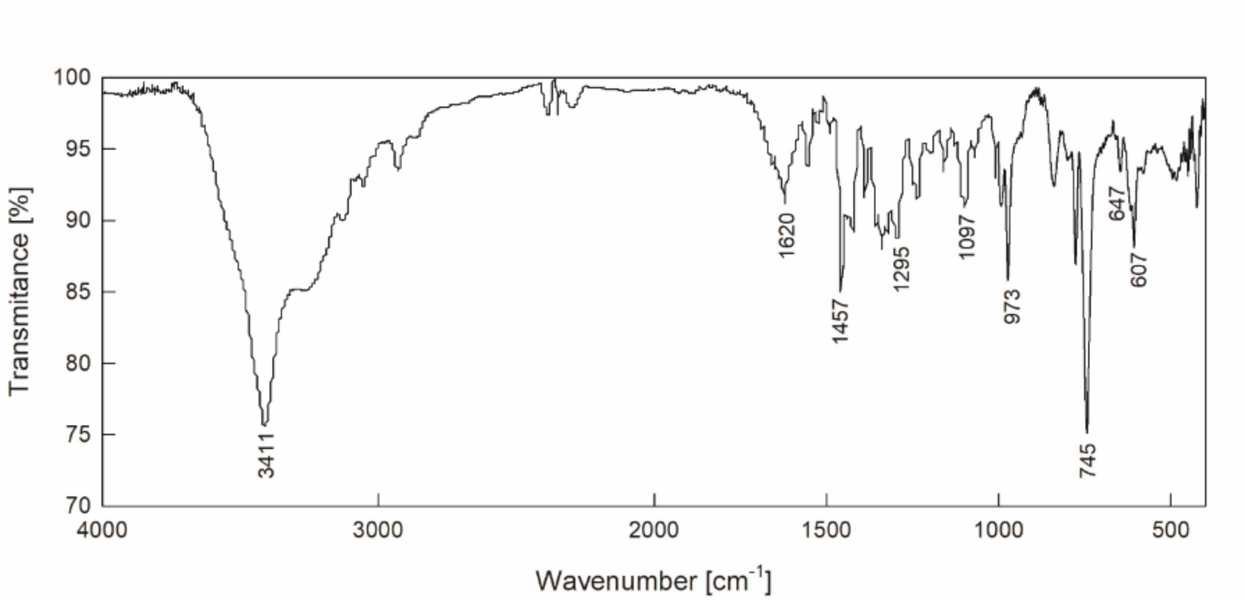

Figure S10d. FT-IR spectrum of compound **11**

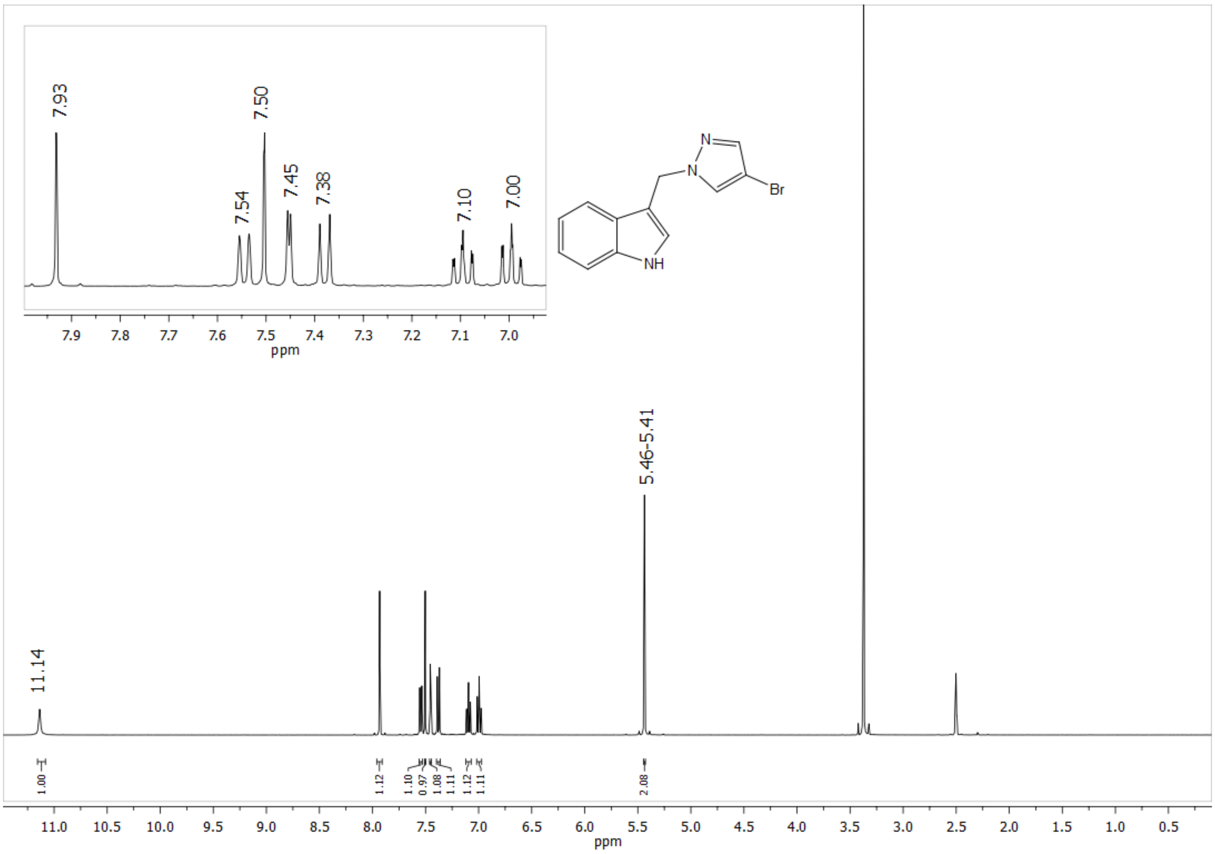

Figure S11a. ^{1}H NMR spectrum of compound **12**

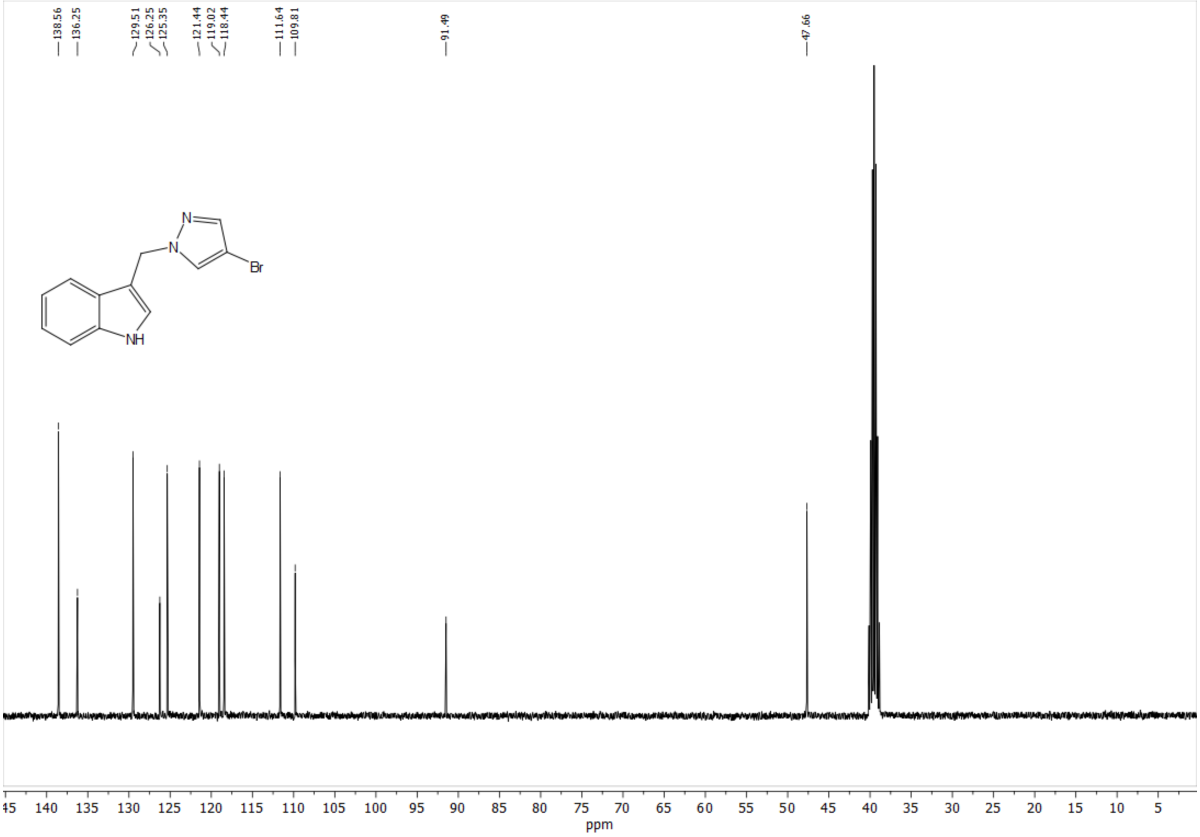

Figure S11b. ^{13}C NMR spectrum of compound **12**

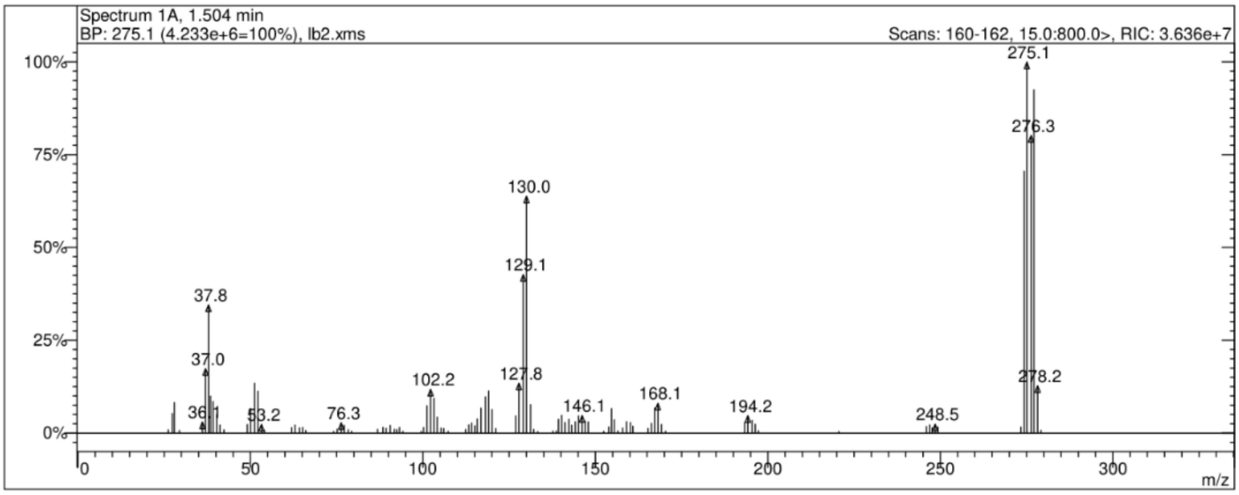

Figure S11c. EI-MS spectrum of compound **12**

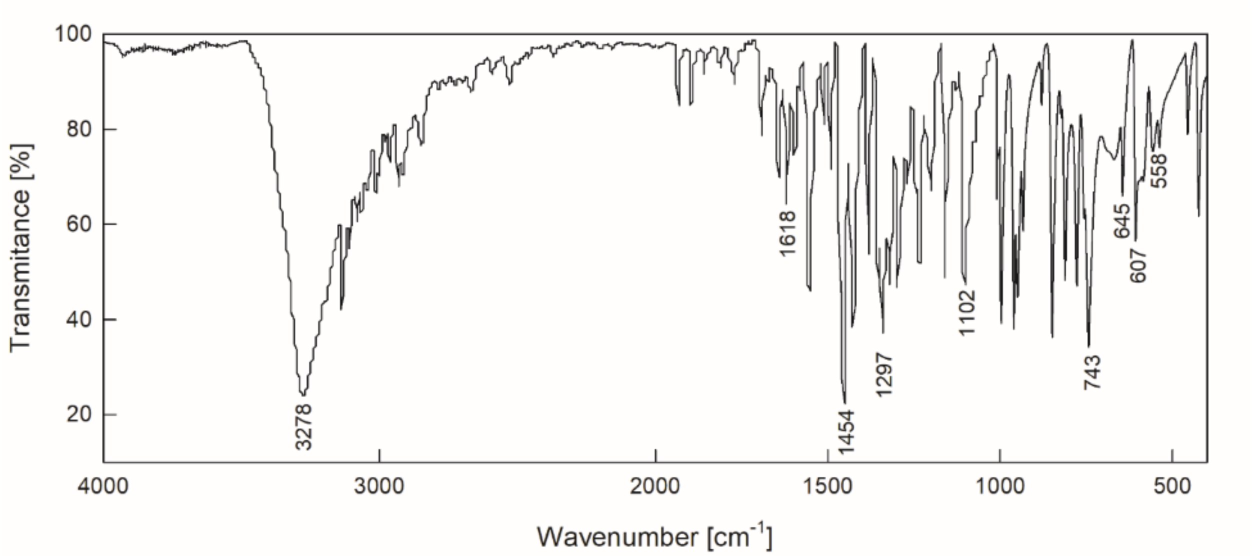

Figure S11d. FT-IR spectrum of compound **12**

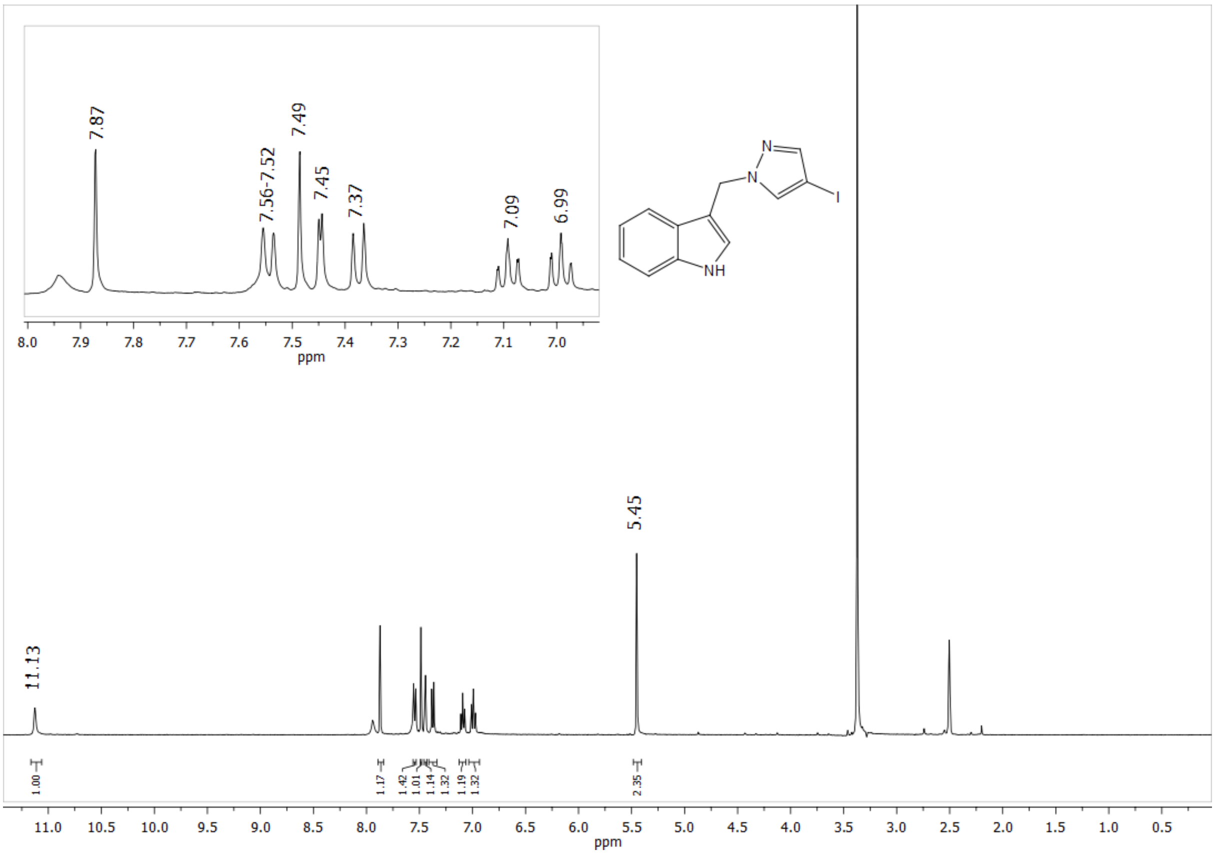

Figure S12a. ^{1}H NMR spectrum of compound **13**

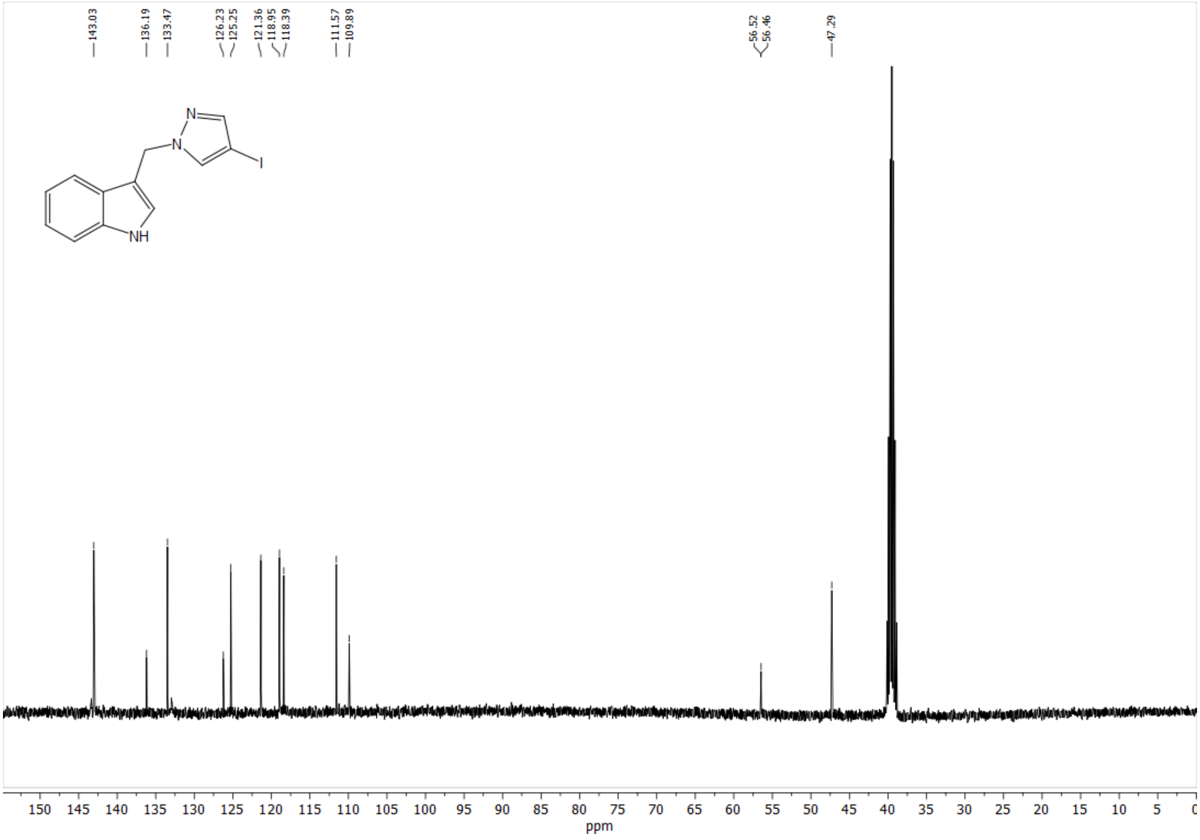

Figure S12b. ^{13}C NMR spectrum of compound **13**

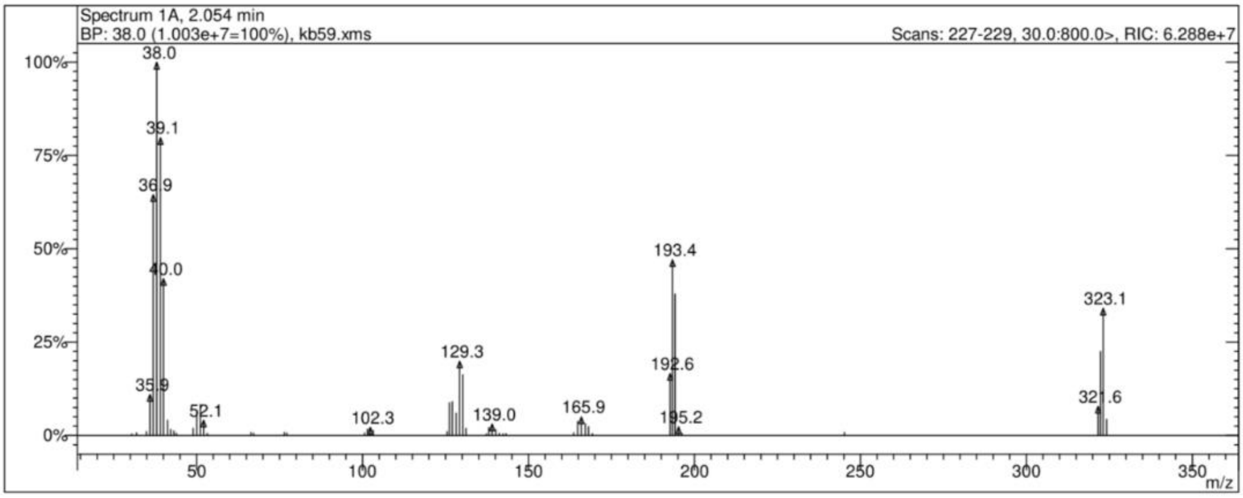

Figure S12c. EI-MS spectrum of compound **13**

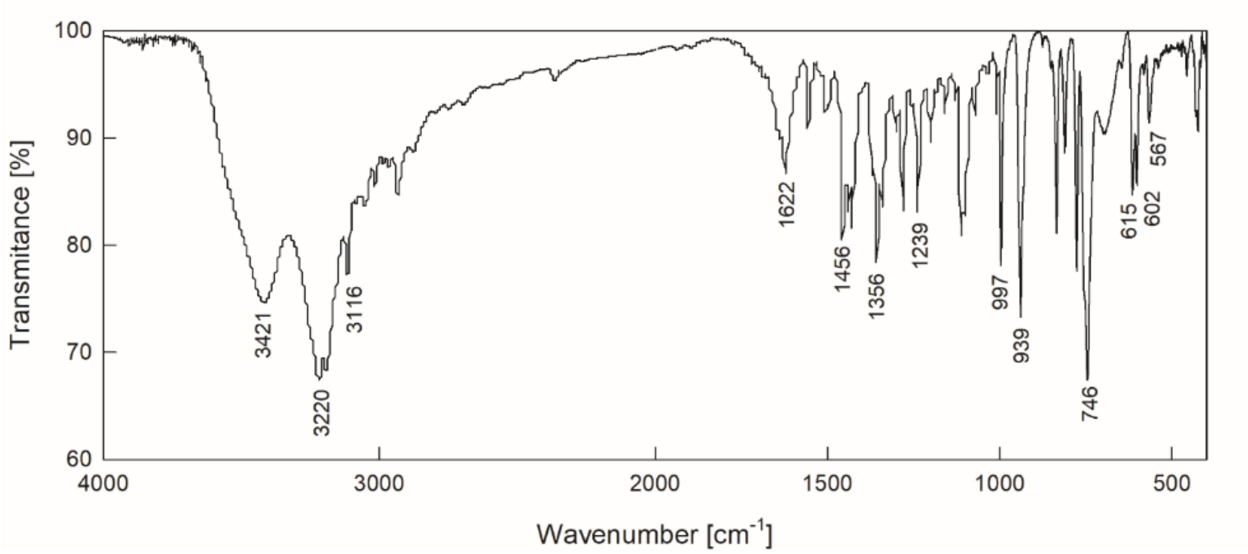

Figure S12d. FT-IR spectrum of compound **13**

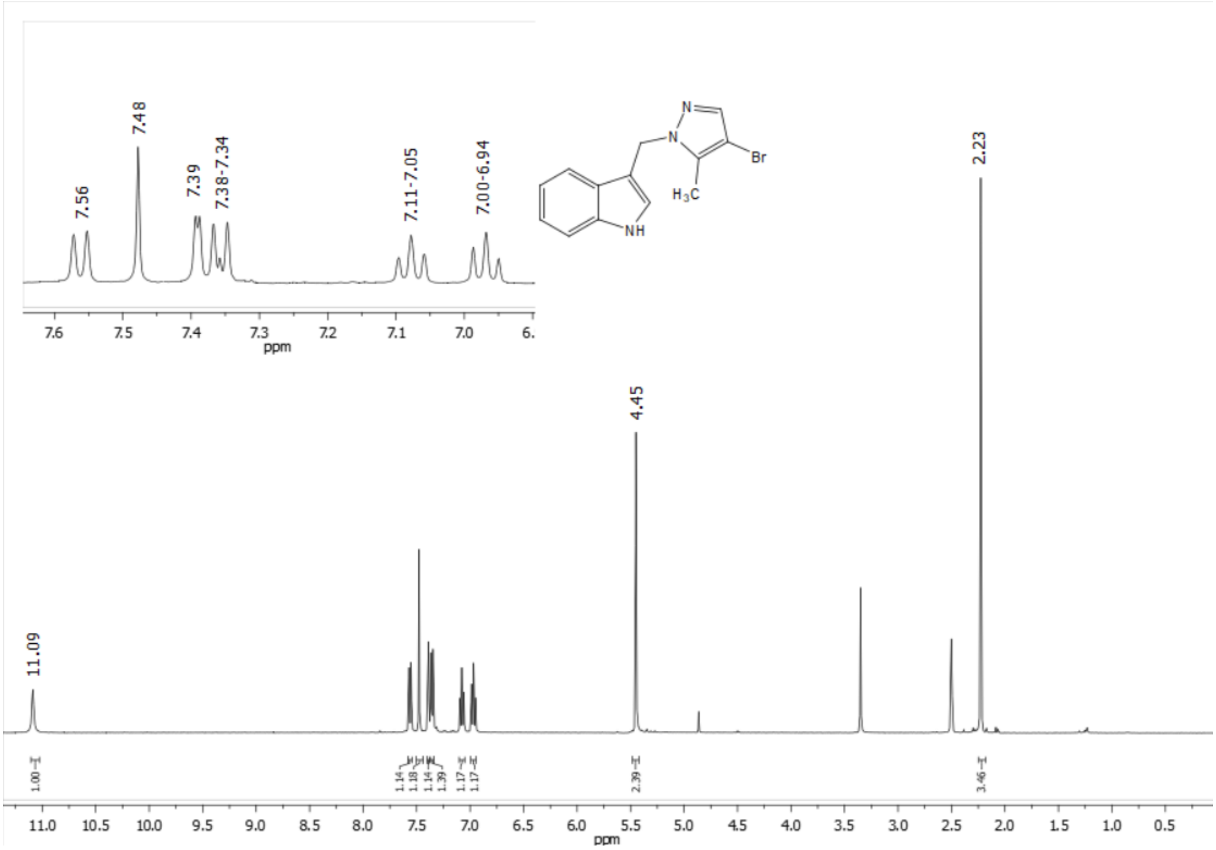

Figure S13a. ^{1}H NMR spectrum of compound **14**

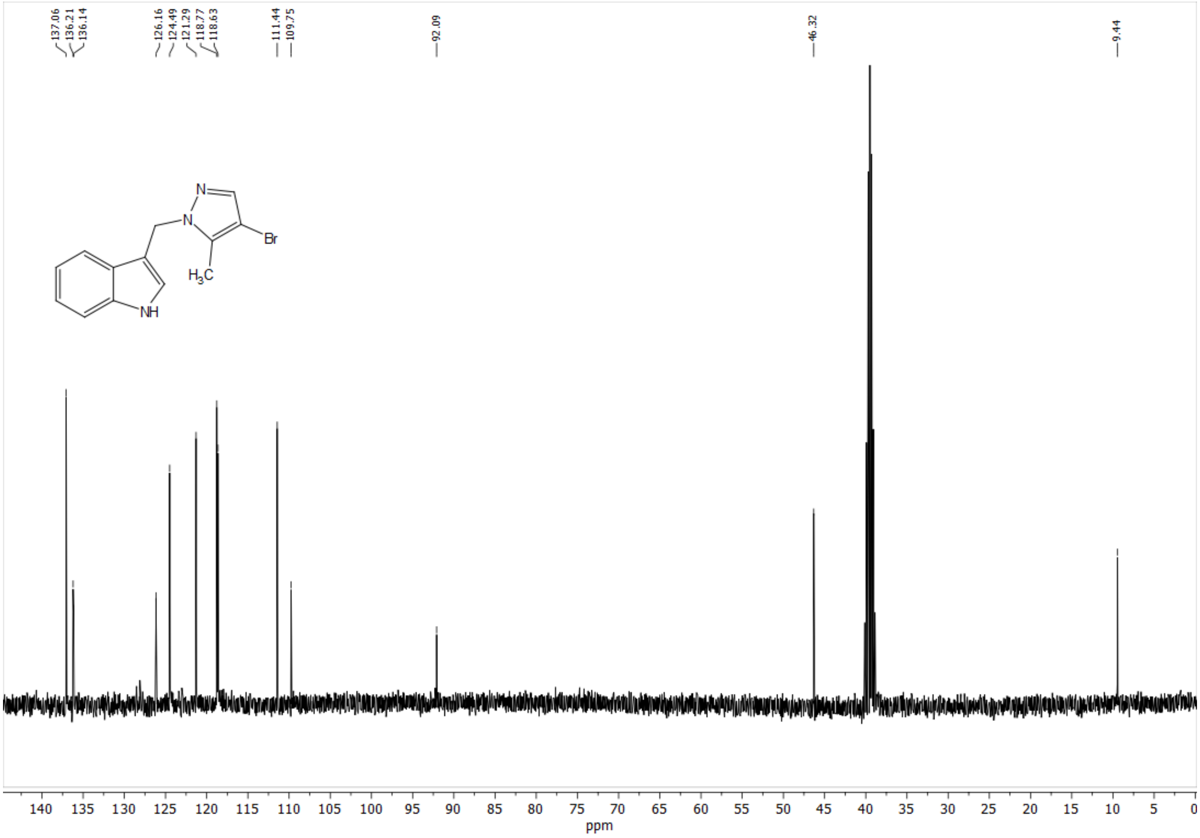

Figure S13b. ^{13}C NMR spectrum of compound **14**

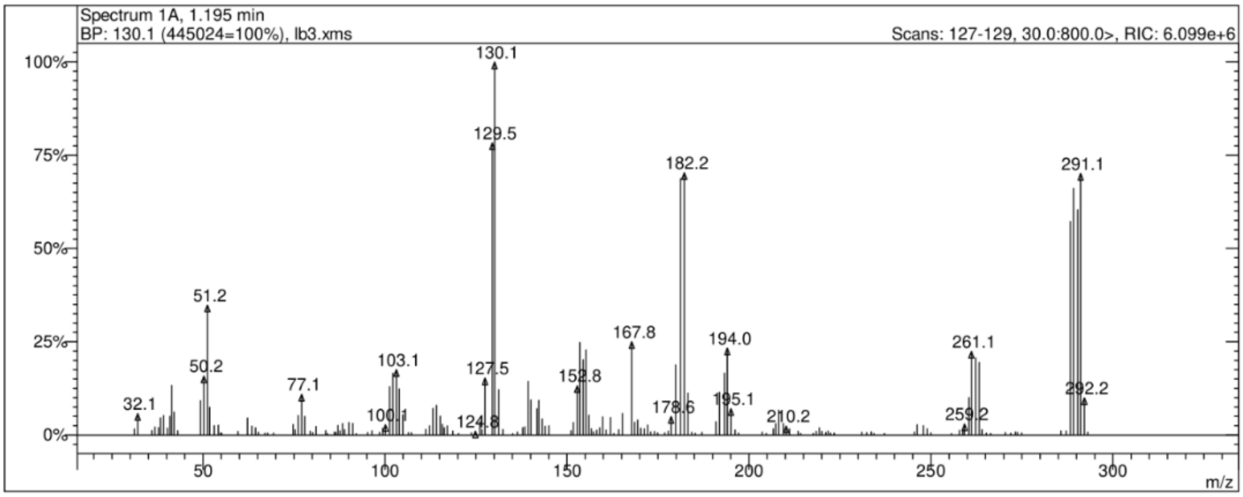

Figure S13c. EI-MS spectrum of compound **14**

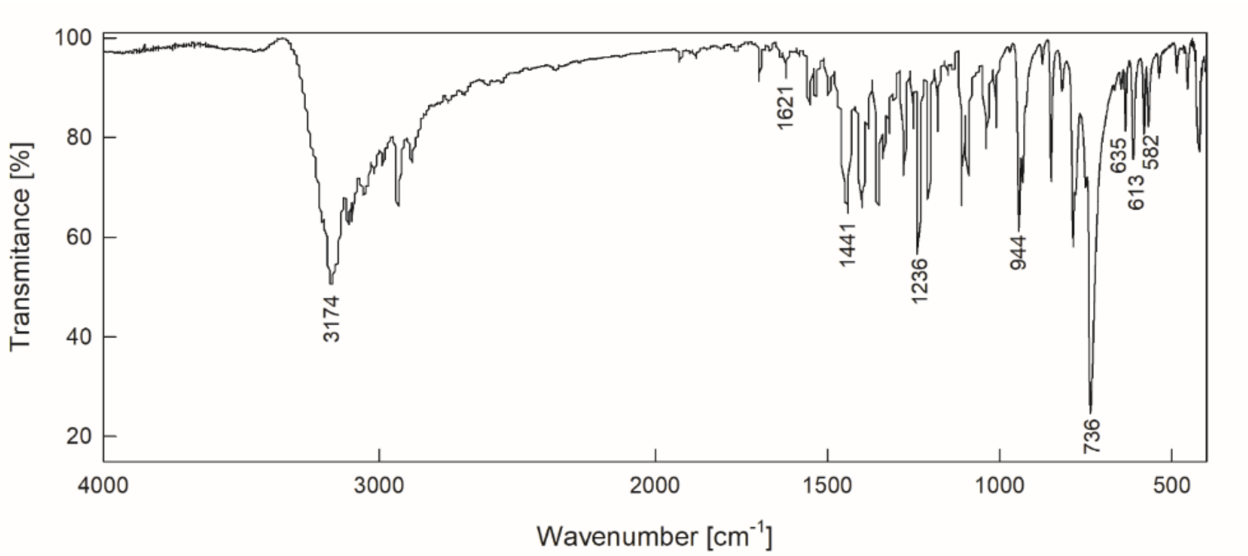

Figure S13d. FT-IR spectrum of compound **14**

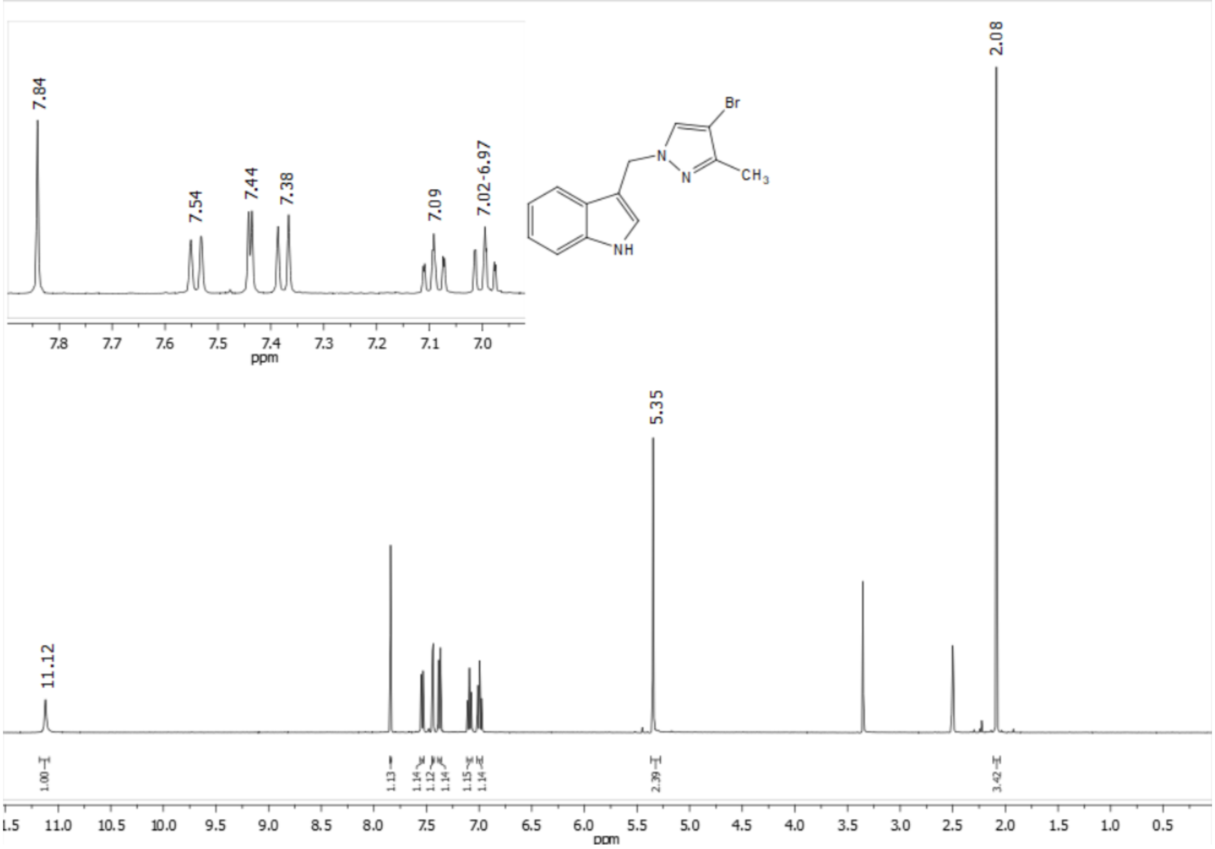

Figure S14a. ^{1}H NMR spectrum of compound **15**

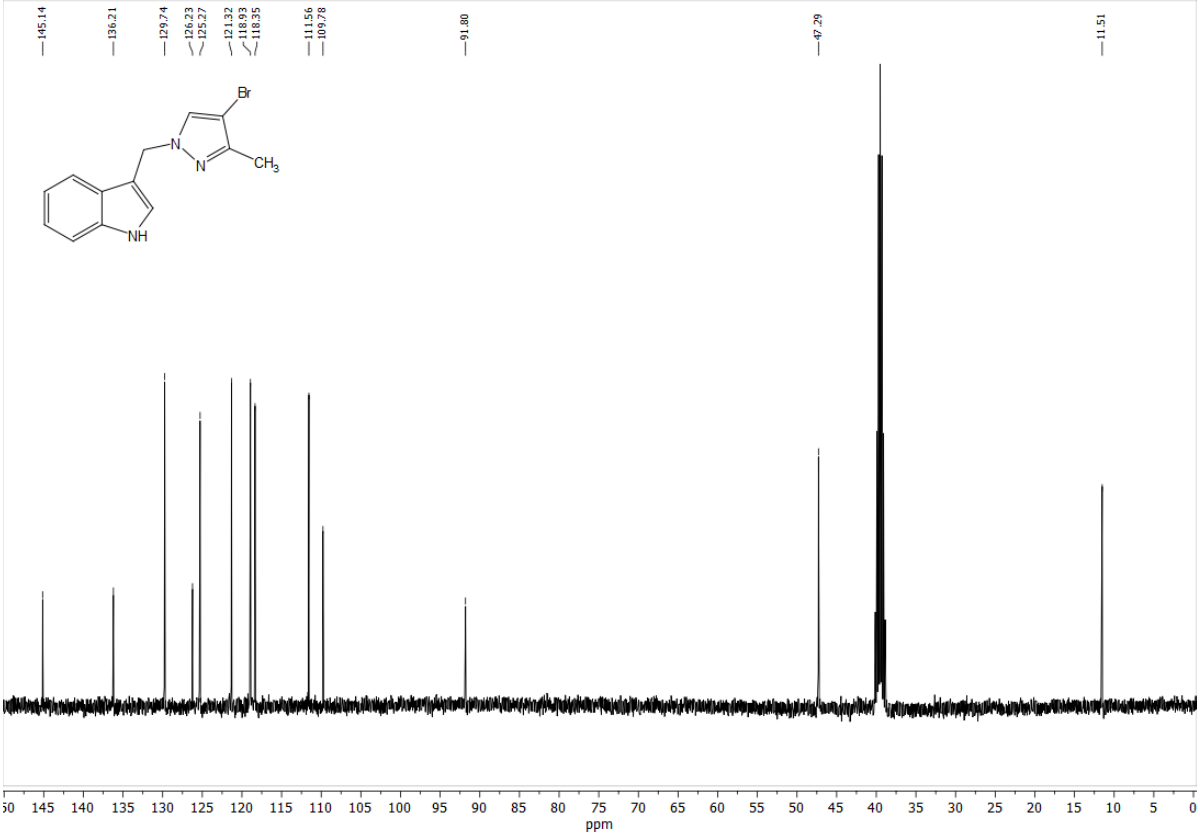

Figure S14b. ^{13}C NMR spectrum of compound **15**

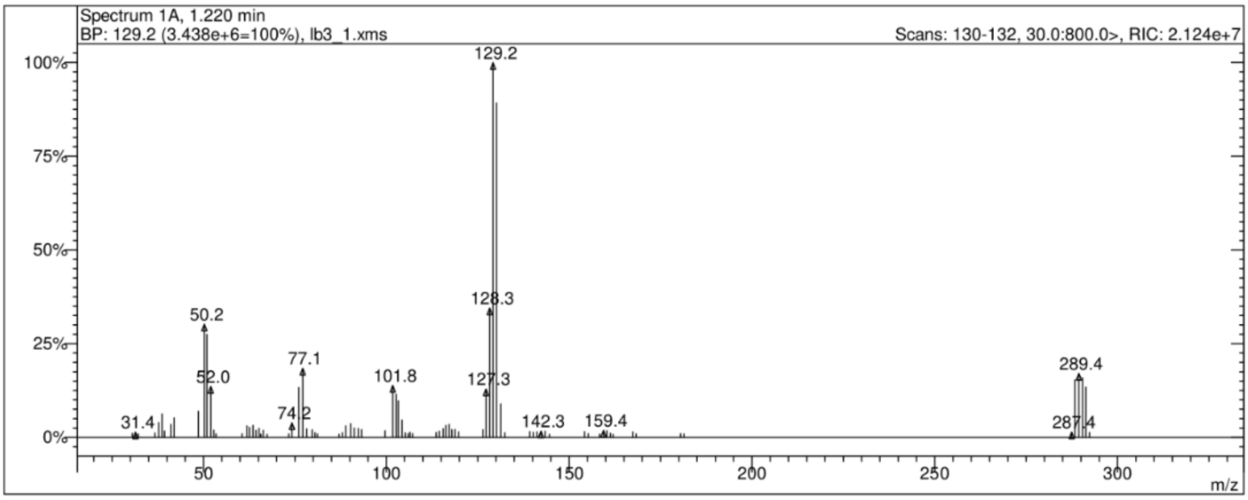

Figure S14c. EI-MS spectrum of compound **15**

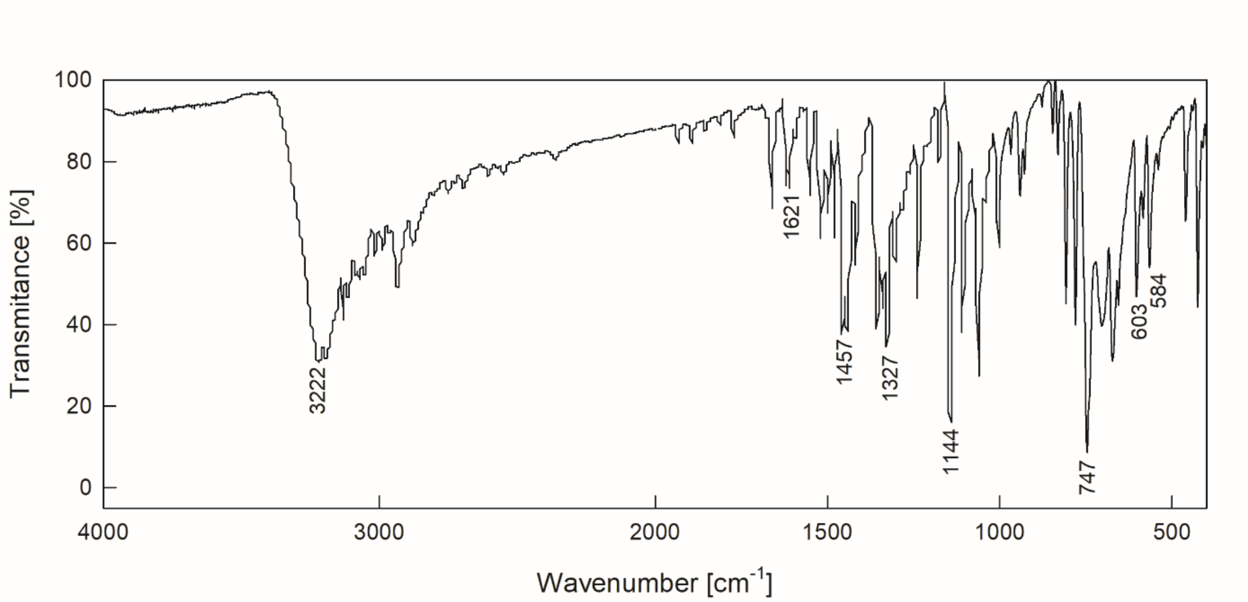

Figure S14d. FT-IR spectrum of compound **15**

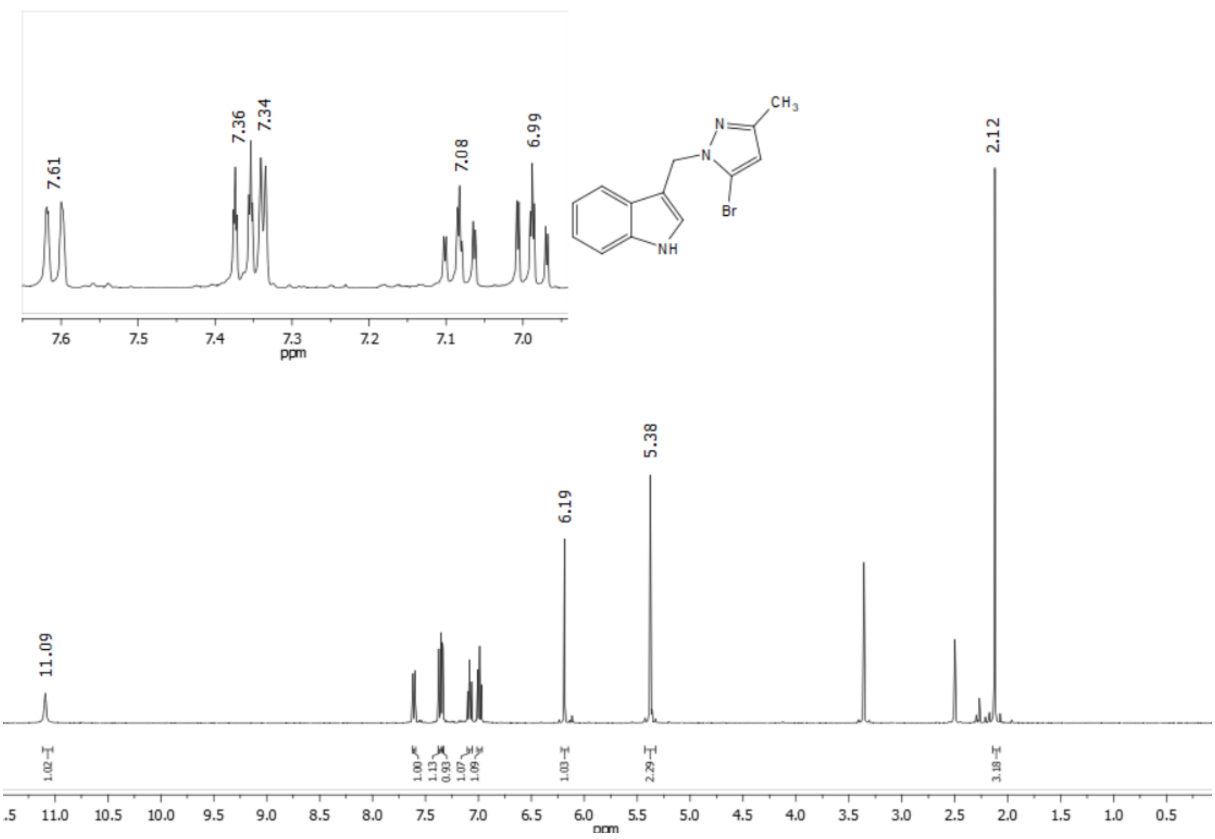

Figure S15a. ^{1}H NMR spectrum of compound **16**

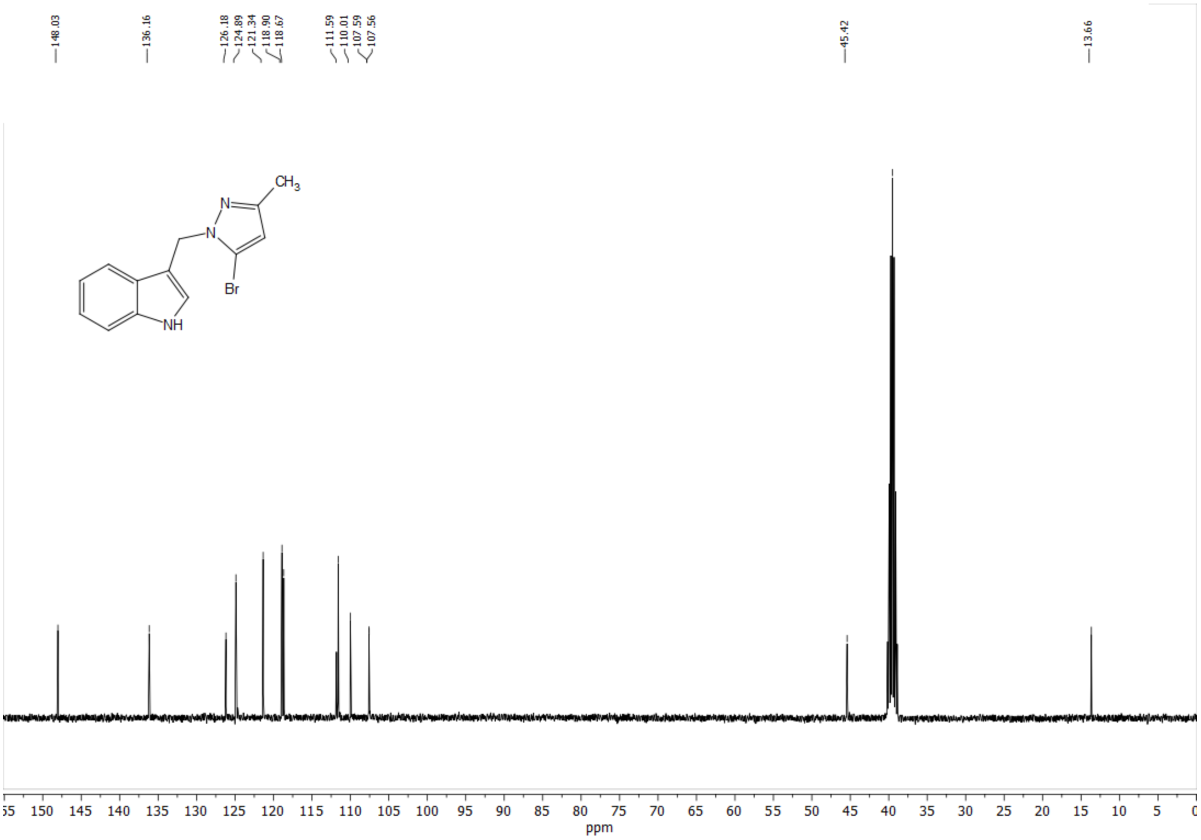

Figure S15b. ^{13}C NMR spectrum of compound **16**

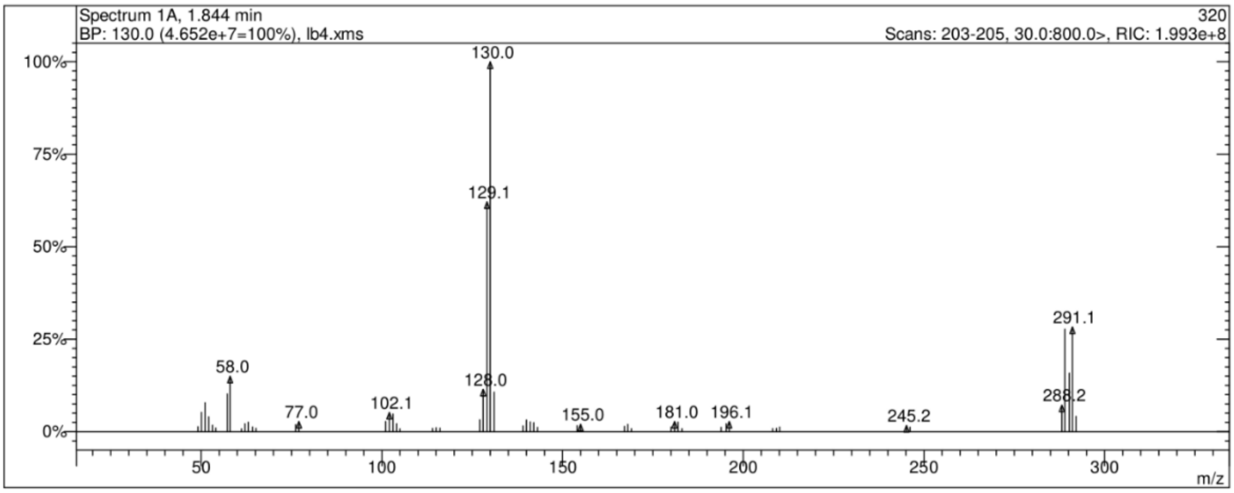

Figure S15c. EI-MS spectrum of compound **16**

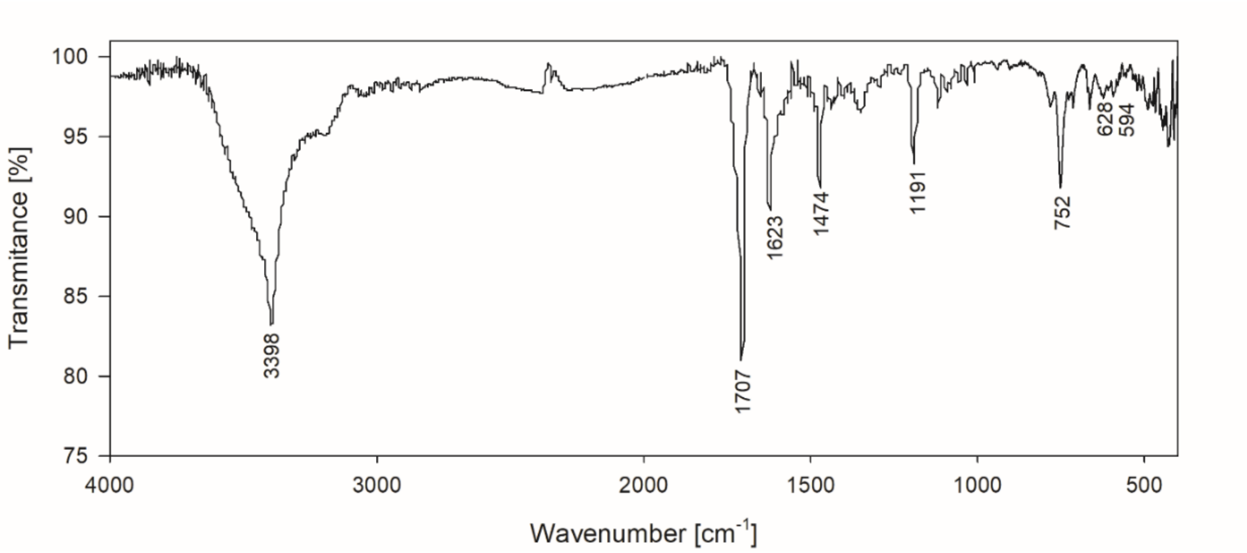

Figure S15d. FT-IR spectrum of compound **16**

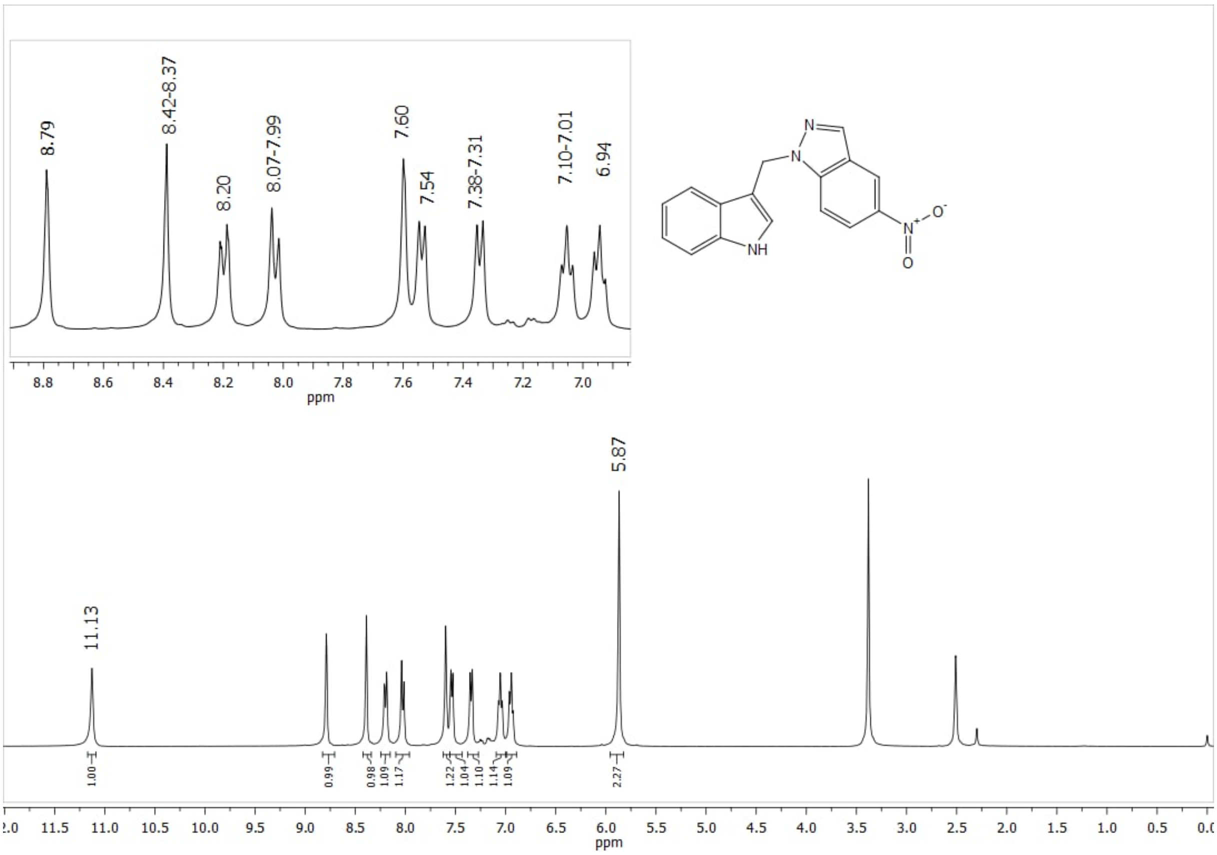

Figure S16a. ^{1}H NMR spectrum of compound **18**

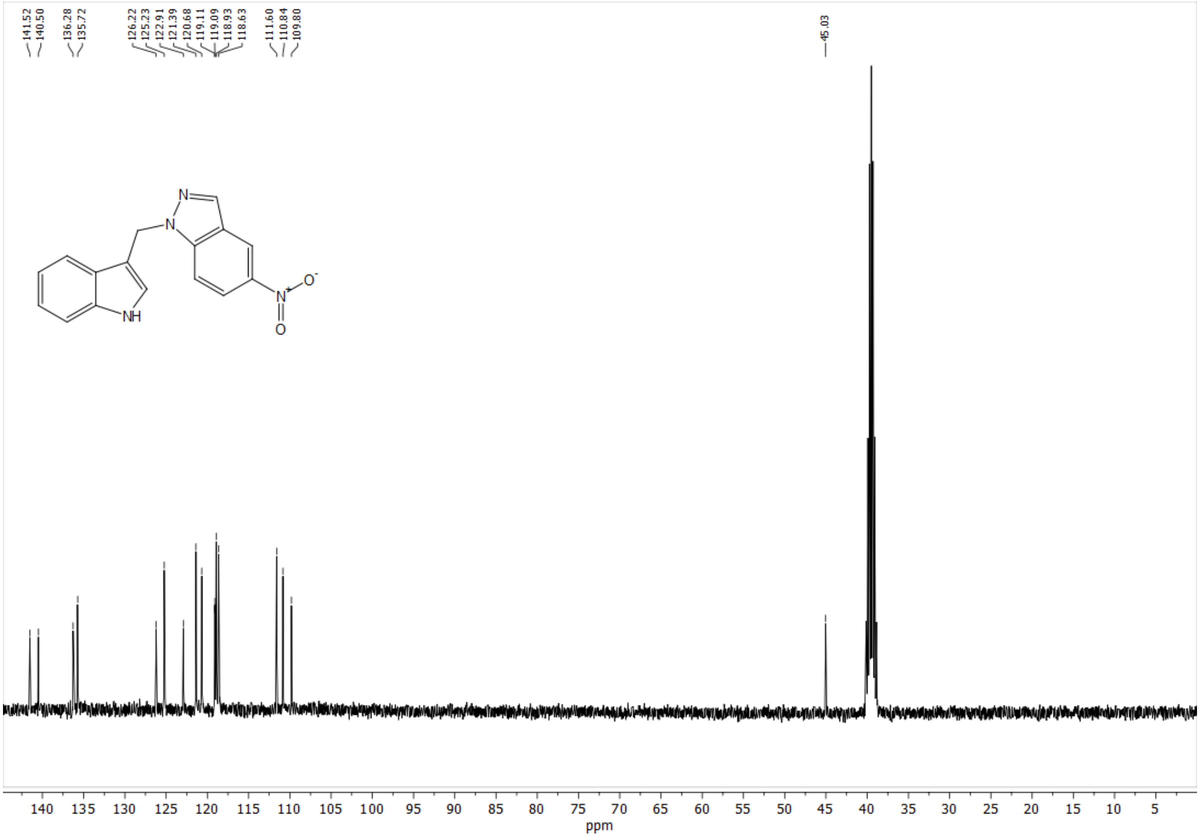

Figure S16b. ^{13}C NMR spectrum of compound **18**

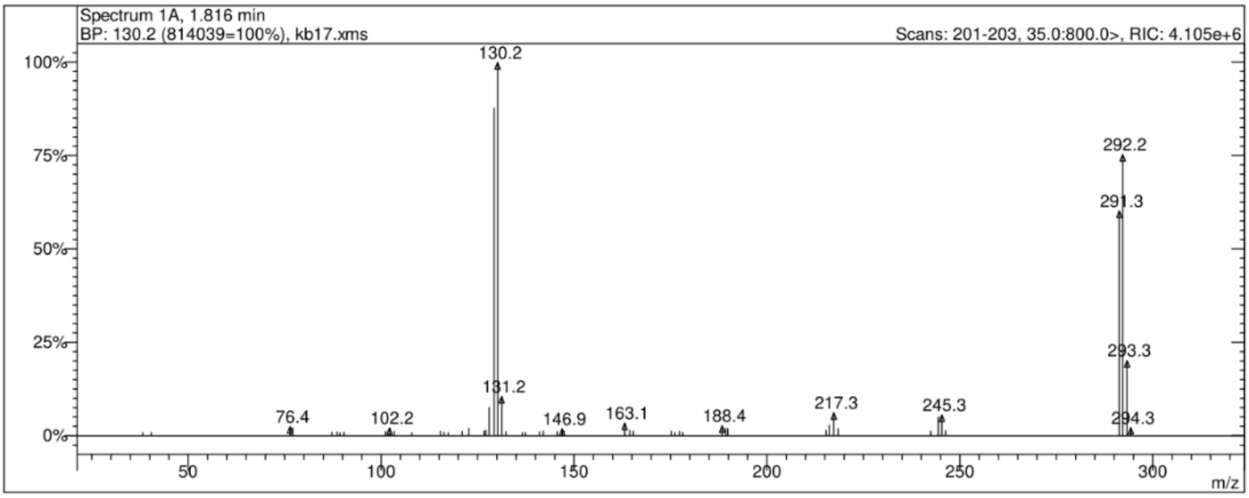

Figure S16c. EI-MS spectrum of compound **18**

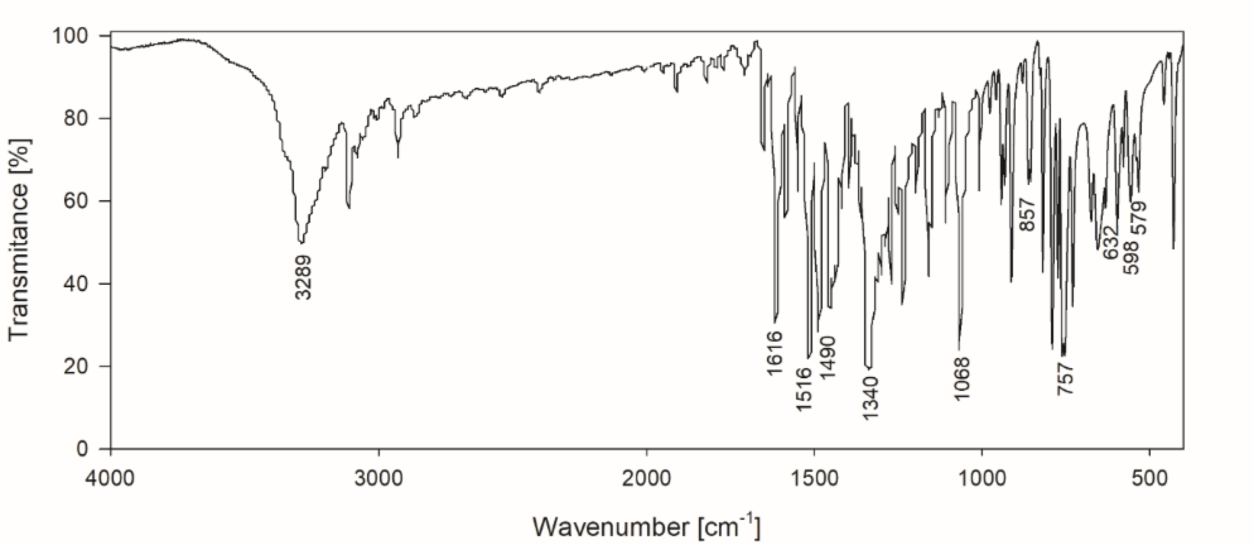

Figure S16d. FT-IR spectrum of compound **18**

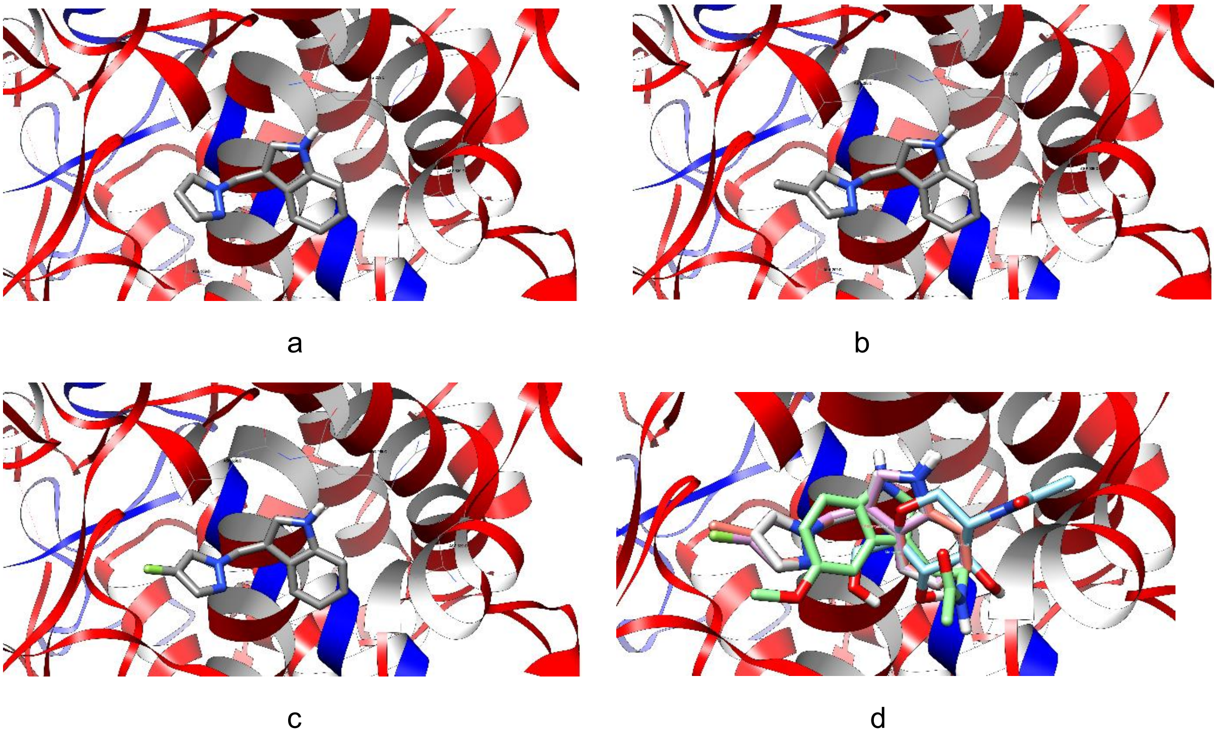

Figure S17. The depiction of the neighborhood inside the 1DNU protein domain of compounds **2** (**17a**), **3** (**17b**), and **10** (**17c**). Native ligands (NAG), reference ligands (melatonin), and new indole—based ligands at once in the binding site of the 1DNU protein domain (**17d**).

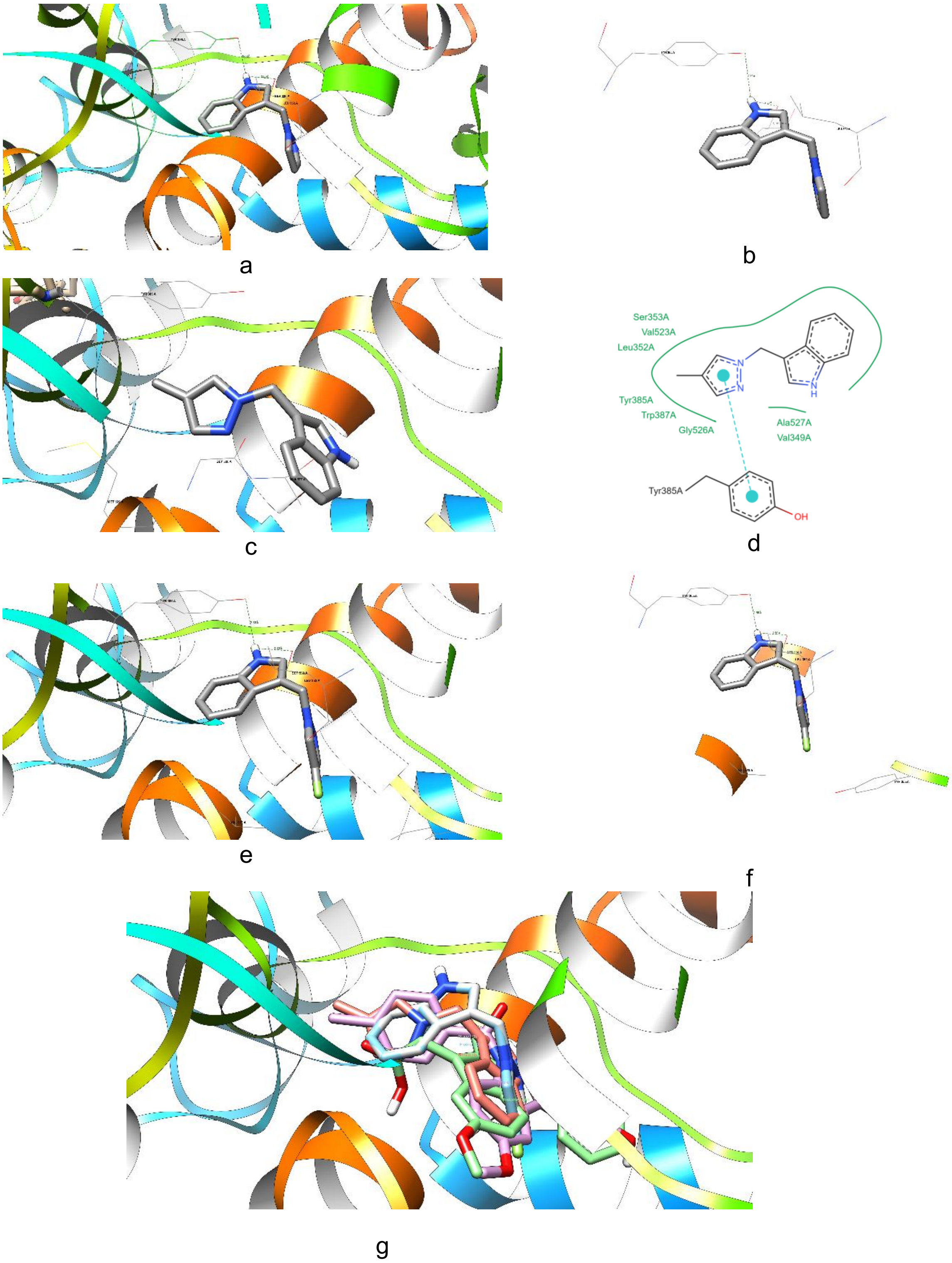

Figure S18. The depiction of possible hydrogen bond formation between the 4COX protein domain and compounds **2** (**18a**) and **10** (**18e**). The very close surrounding of compounds **2** (**18b**) and **10** (**18f**) inside the 4COX protein domain. The depiction of compound **3** neighborhood inside the 4COX protein domain (**18c**) and the 2D depiction of interactions between compound **3** and 4COX protein domain (**18d**). Cyan lines with big dots — pi-pi interactions, green solid lines — hydrophobic contacts. Native ligands (IMN), reference ligand (indometacin), and new ligands at once in the binding site of a 4COX protein domain (**18g**).

Table S1. X-ray experimental details.

	**2**	**3**	**4**	**5**
Crystal data				
Chemical formula	$C_{12}H_{11}N_3$	$C_{13}H_{13}N_3$	$C_{14}H_{15}N_3$	$C_{15}H_{17}N_3$
M_r	197.24	211.26	225.29	239.31
Crystal system, space group	Monoclinic, $P2_1$	Orthorhombic, *Pbcn*	Monoclinic, $P2_1/c$	Orthorhombic, *Pbca*
Temperature (K)	295	295	100	100
a, *b*, *c* (Å)	5.4098 (5),<br>8.9487 (10),<br>21.681 (2)	21.8500 (6),<br>12.0582 (4),<br>8.9245 (3)	11.4734 (1),<br>13.8648 (2),<br>16.0690 (2)	11.9012 (17),<br>8.8429 (8),<br>25.251 (3)
a, b, g (°)	90, 90.101 (4), 90	90, 90, 90	90, 109.4528 (4), 90	90, 90, 90
V (Å^3)	1049.59 (19)	2351.35 (13)	2410.28 (5)	2657.5 (5)
Z	4	8	8	8
D_x (Mg m^{-3})	1.248	1.194	1.242	1.196
Radiation type	Mo *K*a	Cu *K*a	Cu *K*a	Cu *K*a
□ (mm^{-1})	0.08	0.58	0.59	0.57
Crystal size (mm)	0.50 × 0.25 × 0.12	0.6 × 0.2 × 0.08	0.30 × 0.25 × 0.25	0.16 × 0.09 × 0.08
Data collection				
No. of measured, independent and observed [*I* > 2□(*I*)] reflections	47642, 3862, 3134	42986, 2329, 1945	39243, 4723, 4512	19138, 2332, 1643
R_{int}	0.073	0.046	0.029	0.112
(sin q/l)$_{max}$ (Å^{-1})	0.605	0.619	0.617	0.596
Refinement				
$R[F^2 > 2\sigma(F^2)]$, $wR(F^2)$, S	0.035, 0.093, 1.06	0.048, 0.140, 1.05	0.037, 0.095, 1.06	0.051, 0.129, 1.02
No. of reflections	3862	2329	4723	2332
No. of parameters	271	146	310	166
No. of restraints	1	0	0	0
Dr$_{max}$, Dr$_{min}$ (e Å^{-3})	0.12, -0.15	0.16, -0.17	0.31, -0.20	0.28, -0.20

	**6**	**7**	**10**	**11**
Crystal data				
Chemical formula	$C_{15}H_{17}N_3$	$C_{14}H_{15}N_3$	$C_{12}H_{10}FN_3$	$C_{12}H_{10}ClN_3$
M_r	239.31	225.29	215.23	231.68
Crystal system, space group	Orthorhombic, $P2_12_12_1$	Monoclinic, $P2_1/n$	Monoclinic, $P2_1/c$	Orthorhombic, *Pbcn*
Temperature (K)	100	295	100	295
a, *b*, *c* (Å)	6.4823 (6),<br>8.2204 (7),<br>24.000 (2)	9.0321 (2),<br>8.4870 (2),<br>16.6037 (5)	11.7911 (3),<br>8.9705 (2),<br>19.7070 (4)	21.6807 (5), 12.0519 (3),<br>8.8505 (2)
a, b, g (°)	90, 90, 90	90, 104.440 (2), 90	90, 97.908 (1), 90	90, 90, 90
V (Å^3)	1278.9 (2)	1232.56 (6)	2064.63 (8)	2312.58 (9)
Z	4	4	8	8
D_x (Mg m^{-3})	1.243	1.214	1.385	1.331

Radiation type	Cu *K*□	Cu *K*□	Cu *K*□	Cu *K*□
□ (mm^{-1})	0.59	0.58	0.82	2.71
Crystal size (mm)	0.79 × 0.39 × 0.11	0.4 × 0.03 × 0.03	0.3 × 0.3 × 0.01	0.35 × 0.25 × 0.15
Data collection				
No. of measured, independent and observed [$I > 2$□(I)] reflections	29235, 2221, 2191	17635, 2182, 1745	38628, 4081, 3720	23028, 2297, 2057
R_{int}	0.046	0.049	0.029	0.029
(sin q/l)$_{max}$ ($Å^{-1}$)	0.598	0.596	0.618	0.618
Refinement				
$R[F^2 > 2\sigma(F^2)]$, $wR(F^2)$, S	0.057, 0.148, 1.37	0.045, 0.128, 1.05	0.034, 0.085, 1.03	0.038, 0.108, 1.04
No. of reflections	2221	2182	4081	2297
No. of parameters	165	157	289	145
No. of restraints	0	0	0	0
Dr$_{max}$, Dr$_{min}$ (e $Å^{-3}$)	0.31, -0.34	0.19, -0.21	0.21, -0.26	0.13, -0.32

	**12**	**13**	**16**
Crystal data			
Chemical formula	$C_{12}H_{10}BrN_3$	$C_{12}H_{10}IN_3$	$C_{13}H_{11.76}BrN_3$
M_r	276.14	323.13	290.17
Crystal system, space group	Orthorhombic, *Pbca*	Monoclinic, $P2_1/c$	Orthorhombic, $P2_12_12_1$
Temperature (K)	295	295	295
a, *b*, *c* (Å)	15.7615 (3), 8.1983 (1), 17.6219 (2)	8.7915 (4), 11.7759 (5), 11.5805 (5)	9.5365 (2), 10.7056 (3), 11.9665 (3)
a, b, g (°)	90, 90, 90	90, 101.821 (1), 90	90, 90, 90
V ($Å^3$)	2277.06 (6)	1173.48 (9)	1221.71 (5)
Z	8	4	4
D_x (Mg m^{-3})	1.611	1.829	1.578
Radiation type	Mo *K*□	Mo *K*□	Mo *K*□
□ (mm^{-1})	3.59	2.70	3.35
Crystal size (mm)	0.4 × 0.4 × 0.1	0.40 × 0.30 × 0.12	0.25 × 0.17 × 0.12
Data collection			
No. of measured, independent and observed [$I > 2$□(I)] reflections	93278, 2002, 1758	37851, 2066, 1936	46629, 2489, 2373
R_{int}	0.048	0.045	0.057
(sin q/l)$_{max}$ ($Å^{-1}$)	0.595	0.595	0.625
Refinement			
$R[F^2 > 2\sigma(F^2)]$, $wR(F^2)$, S	0.035, 0.083, 1.08	0.024, 0.063, 1.05	0.020, 0.048, 1.03
No. of reflections	2002	2066	2489
No. of parameters	145	145	162
No. of restraints	0	0	14
Dr$_{max}$, Dr$_{min}$ (e $Å^{-3}$)	0.60, -0.69	0.87, -0.97	0.15, -0.23

Table S2. The search spaces of the analyzed binding sites of the protein domains.

PDB ID	Search Space Center (x,y,z)	Size of the Search Space (x,y,z)
1DNU	40, -38, -5	27, 24, 25
1N5X	97, 55, 39	25, 27, 33
4COX	25, 22, 15	28, 27, 30
